# Supplementary material for: Macrophage-Derived Extracellular Succinate Licenses Neural Stem Cells to Suppress Chronic Neuroinflammation
Source: Cell Stem Cell. 2018 Mar 1;22(3):355–368.e13. doi: 10.1016/j.stem.2018.01.020 (PMC5842147; doi:10.1016/j.stem.2018.01.020)
Supplement: Document S1. Figures S1–S6 [file mmc1.pdf]

Cell Stem Cell, Volume *22*

Supplemental Information

Macrophage-Derived Extracellular Succinate Licenses Neural Stem Cells to Suppress Chronic Neuroinflammation

Luca Peruzzotti-Jametti, Joshua D. Bernstock, Nunzio Vicario, Ana S.H. Costa, Chee Keong Kwok, Tommaso Leonardi, Lee M. Booty, Iacopo Bicci, Beatrice Balzarotti, Giulio Volpe, Giulia Mallucci, Giulia Manferrari, Matteo Donegà, Nunzio Iraci, Alice Braga, John M. Hallenbeck, Michael P. Murphy, Frank Edenhofer, Christian Frezza, and Stefano Pluchino

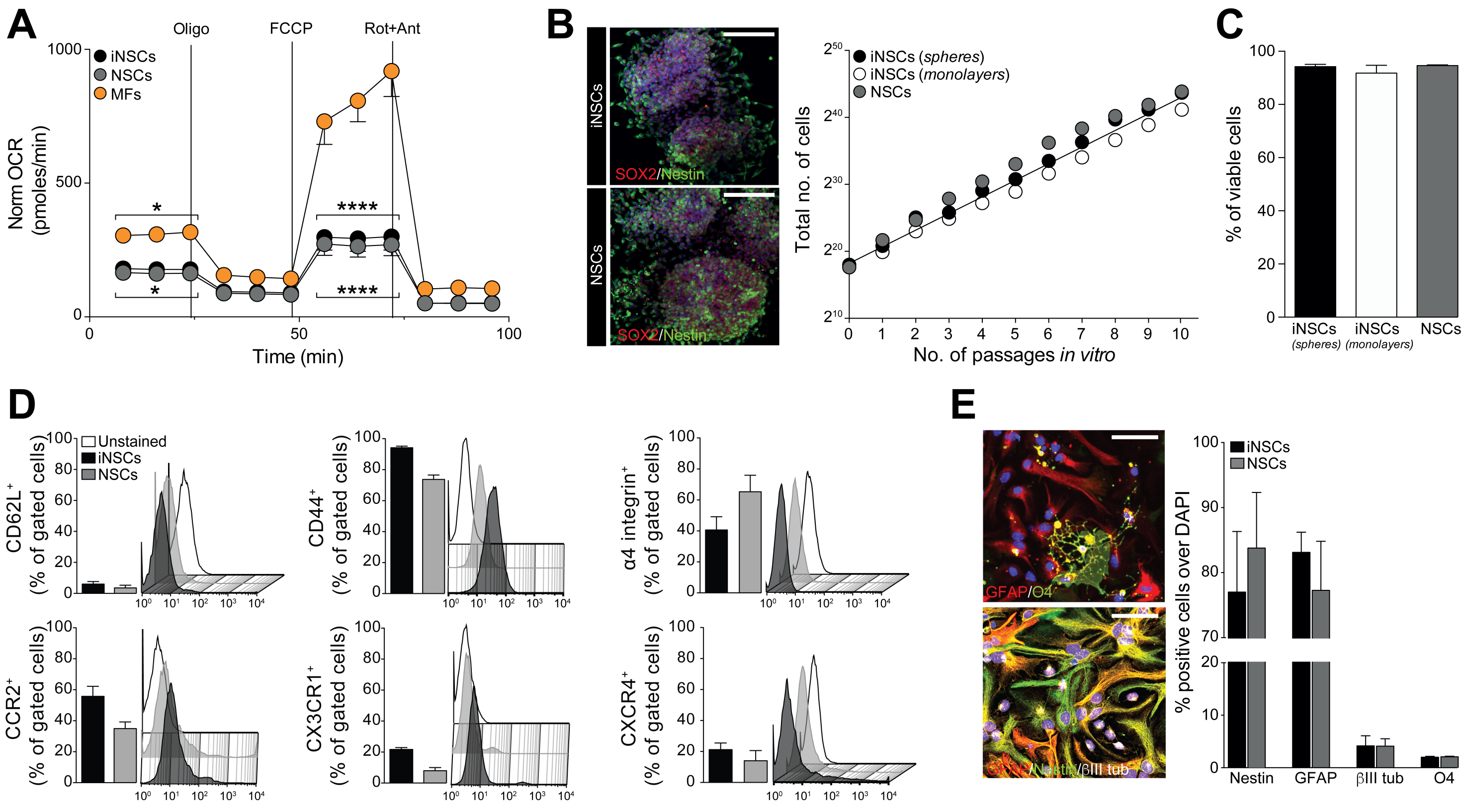

Peruzzotti-Jametti et al. Figure S1

Figure S1. Related to Figure 1. Mouse iNSCs and NSCs *in vitro* features

(A) Extracellular Flux (XF) assay of the Oxygen Consumption Rate (OCR) during a mitochondrial stress protocol. Data are normalized on total protein content and expressed as mean values (± SEM) from n≥ 3 independent experiments.

(B) Representative confocal microscopy images of iNSCs and NSCs growing *in vitro* as SOX2$^+$/Nestin$^+$ neurospheres. Nuclei are stained with DAPI. Linear growth curves of iNSCs and NSCs over 10 passages *in vitro*. Data are mean numbers (± SEM) from n≥ 3 independent experiments per condition.

(C) Cell viability as in B. Data are mean percentages of viable cells (± SEM) from n≥ 3 independent experiments per condition.

(D) Fluorescence-activated cell sorting (FACS) analysis. Data are positive cells over gated cells (mean percentages ± SEM) from n≥ 3 independent experiments per condition.

(E) *In vitro* differentiation properties of iNSCs and NSCs. Representative confocal microscopy images of differentiated iNSCs stained for GFAP, O4, Nestin and βIII tubulin are shown. Nuclei were stained with DAPI. Data are mean percentages of positive cells (± SEM) from n≥ 3 independent experiments per condition.

Scale bars: 100 µm (B), 40 µm (E). *p≤ 0.05 and ****p≤ 0.0001, *vs.* Mouse Fibroblasts (MFs).

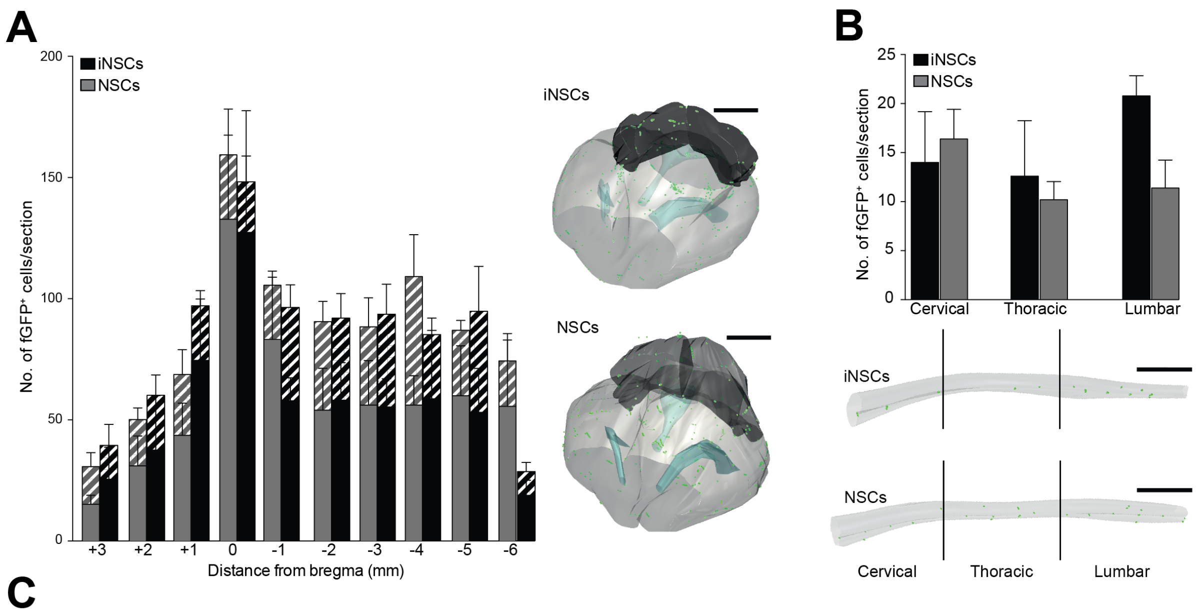

C

iNSCs			NSCs		
Mean ± SEM	Brain	Spinal Cord	Mean ± SEM	Brain	Spinal Cord
No. of fGFP+ cells	33,344 ± 7,505	1,896 ± 338	No. of fGFP+ cells	29,780 ± 4,551	1,520 ± 237
% Nestin+/fGFP+	1.03 ± 1.03	ND	% Nestin+/fGFP+	0.90 ± 0.90	1.54 ± 0.81
% Ki67+/fGFP+	2.11 ± 0.87	ND	% Ki67+/fGFP+	1.73 ± 0.11	ND
% NeuN+/fGFP+	2.28 ± 0.65	ND	% NeuN+/fGFP+	2.03 ± 0.32	ND
% GFAP+/fGFP+	15.04 ± 2.67	23.81 ± 12.40	% GFAP+/fGFP+	12.48 ± 2.40	31.34 ± 3.96
% OLIG2+/fGFP+	5.12 ± 2.03	ND	% OLIG2+/fGFP+	4.89 ± 0.71	ND
% DCX+/fGFP+	1.32 ± 0.30	ND	% DCX+/fGFP+	1.24 ± 0.20	ND

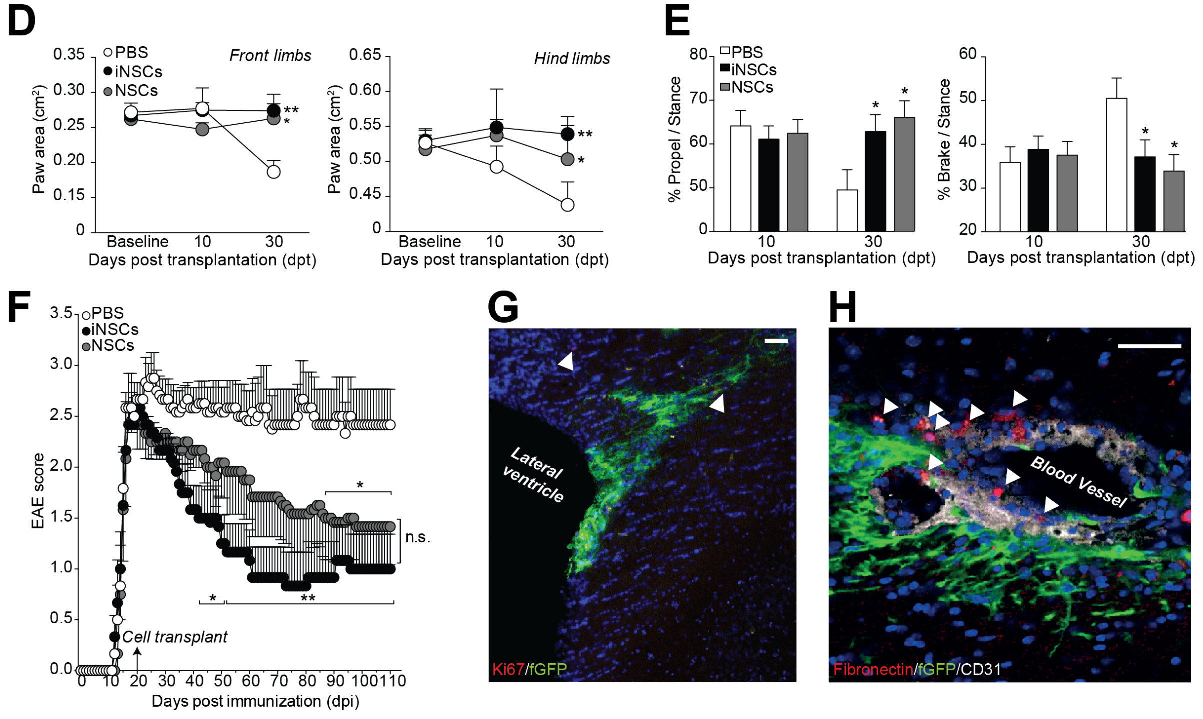

Peruzzotti-Jametti et al. Figure S2

Figure S2. Related to Figure 1. Distribution, efficacy and safety of icv-injected iNSCs and NSCs

(A-B) Distribution in the brain and spinal cord of $fGFP^+$ iNSCs or $fGFP^+$ NSCs injected icv in EAE mice at peak of disease (PD). Stereology-based quantification of whole brain (A) and spinal cord (B) with representative 3D reconstructions (green dots indicate individual $fGFP^+$ cells, contours of the ventricles are in light blue, those of the forebrain or spinal cord are in light grey, and those of the cerebellum are in dark grey). Solid bars in A are data from the left-brain hemisphere, while wide upward diagonal bars are from data of the right brain hemisphere. Data are mean numbers (± SEM) from n≥ 5 mice/group over n= 2 independent experiments. Scale bars: 2 mm.

(C) *In vivo* differentiation profile of transplanted $fGFP^+$ iNSCs or NSCs as in A and B. Data are mean numbers (± SEM) from n≥ 5 mice/group over n= 2 independent experiments.

(D-E) DigiGaitTM ventral plane treadmill videography of EAE mice. (D) Changes in the areas of the paws (front limbs and hind limbs) in iNSC- and NSC-transplanted EAE mice. Data are mean areas (± SEM) from n≥ 5 mice/group. Baseline was assessed before EAE onset. (E) Gait kinematics as in A. Data are percentages of time in which the paw is in the propulsion (propel) or breaking (brake) phase during the stance (± SEM) from n≥ 5 mice/group.

(F) Behavioural outcome of $fGFP^+$ iNSC transplants up to 110 dpi. Data are mean EAE score (± SEM) from n= 6 mice/group.

(G-H) Representative confocal microscopy image of transplanted $fGFP^+$ iNSCs in the brain of a EAE mouse at 110 dpi. Expression of Ki67 by $fGFP^+$ iNSCs is indicated by arrowheads (G). Transplanted $fGFP^+$ iNSCs are negative for fibronectin (arrowheads in H). Scale bars: 50 μm.

*p≤ 0.05 and **p≤ 0.01, *vs.* PBS; n.s.: not significant, ND: not detected.

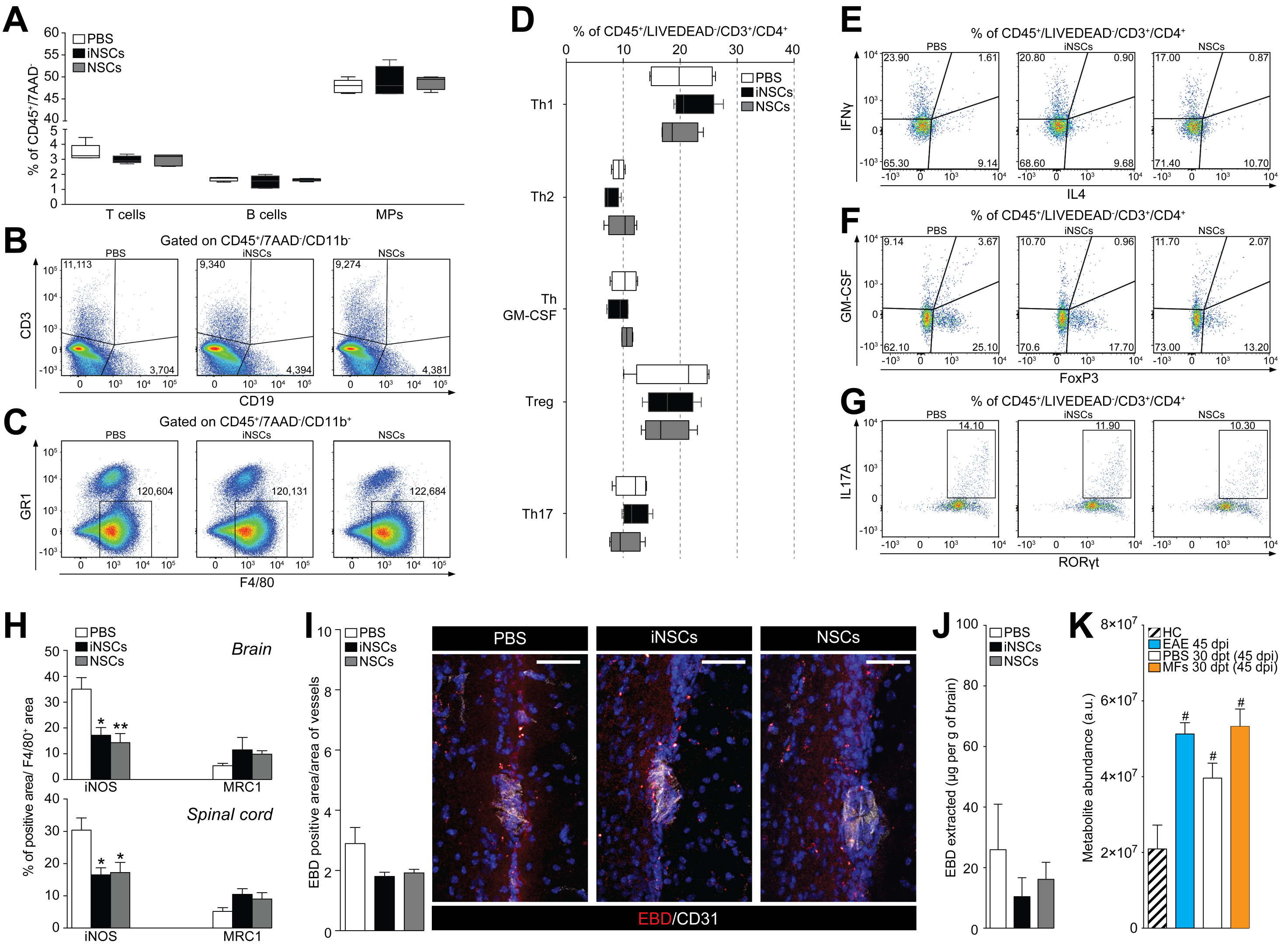

Peruzzotti-Jametti et al. Figure S3

Figure S3. Related to Figure 1. Inflammatory infiltrates and BBB analysis of EAE mice

(A) Flow cytometry *ex vivo* analysis of lymphoid and myeloid cells from the CNS of iNSC- and NSC-transplanted EAE mice at 30 dpt. Cells are gated as $CD45^{+}/7AAD^{-}/CD11b^{-}/CD3^{+}$ (T cells), $CD45^{+}/7AAD^{-}/CD11b^{-}/CD19^{+}$ (B cells), $CD45^{+}/7AAD^{-}/CD11b^{+}/GR1^{-}/F4/80^{+}$ (MPs). Data are min to max % of marker-positive cells from n≥ 4 pools of mice/group.

(B-C) Representative density plots as in A.

(D) Analysis of the $CD3^{+}/CD4^{+}$ T cells subsets as $IFN\gamma^{+}$ (Th1), $IL4^{+}$ (Th2), $GM\text{-}CSF^{+}$ (Th GM-CSF), $FoxP3^{+}$ (Treg) and $IL17A^{+}/ROR\gamma t^{+}$ (Th17). Data are min to max % of marker-positive cells from from n≥ 4 pools of mice/group.

(E-G) Representative density plots as in D.

(H) Quantification of the proportion of $F4/80^{+}$ cells expressing the type-1 inflammatory MPs marker iNOS, or the anti-inflammatory MPs marker MRC1. Data are mean % of positive-stained area/total section area and % of positive-stained area/$F4/80^{+}$ area (± SEM), respectively. Data are from n≥ 5 mice/group over n= 2 independent experiments.

(I) EBD-based quantification of peri-vascular BBB leakage/permeability by image analysis. Representative images of $CD31^+$ vessels with EBD staining in the brain of iNSC-/NSC-transplanted and PBS-treated control EAE mice are shown. Nuclei are stained with DAPI. Data are EBD positive area (± SEM) over the corresponding vascular area from n= 3 mice/group.

(J) Quantification of EBD extracted from brain samples as in I. Data are expressed as μg of EBD per g of tissue (± SEM) from n= 3 mice/group.

(K) Quantification of CSF succinate. EAE 45 dpi are EAE mice not subjected to surgery, while MFs are EAE mice injected icv with MFs as control cells. HC, healthy controls. Data are mean arbitrary units (a.u.) (± SEM) from n≥ 3 mice/group.

Scale bars: 50 μm. EBD: Evans blue dye. *p≤ 0.05 and **p≤ 0.01, *vs.* PBS; #p≤ 0.05, *vs.* HC.

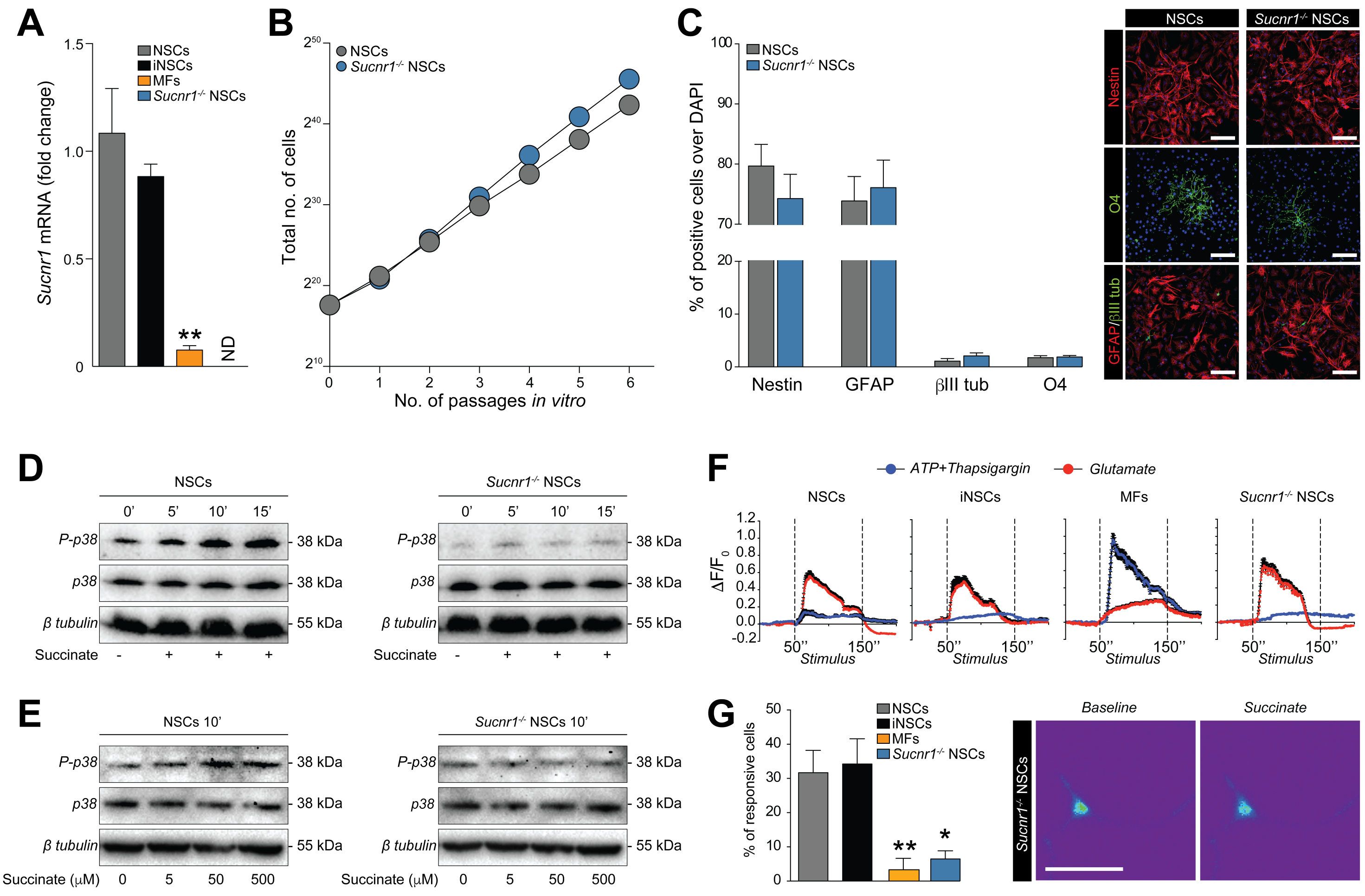

Peruzzotti-Jametti et al. Figure S4

Figure S4. Related to Figure 3. Mouse *Sucnr1*$^{-/-}$ NSCs *in vitro* features

(A) *Sucnr1* mRNA expression relative to *Actb* in mouse cells and *Sucnr1*$^{-/-}$ NSCs. Data are mean fold change (± SEM) *vs.* NSCs from n≥ 3 independent replicates per condition.

(B) Linear growth curves of NSCs and *Sucnr1*$^{-/-}$ NSCs over 6 passages *in vitro*. Data are mean numbers (± SEM) from n≥ 3 independent experiments per condition.

(C) *In vitro* differentiation properties of NSCs and *Sucnr1*$^{-/-}$ NSCs. Representative confocal microscopy images of differentiated cells stained for Nestin, O4, GFAP and βIII tubulin are shown. Nuclei are stained with DAPI. Data are mean percentages of positive cells (± SEM) from n≥ 3 independent experiments per condition.

(D-E) Phospho-p38 MAPK (P-p38) and total p38 MAPK (p38) protein expression in mouse NSCs (as reported in Figure 3G) and *Sucnr1*$^{-/-}$ NSCs after 500 μM succinate at different time points (D), or at 10' under different concentration of succinate (E).

(F) Intracellular Ca^{2+} response after treatment with 500 μM succinate (live staining with Fluo-4AM). Data are % of responsive cells (± SEM) from n≥ 3 experiments. Representative images of *Sucnr1*$^{-/-}$ NSCs (baseline and during stimulation) are pseudocolored with red/blue according to high/low fluorescence intensity.

(G) Intracellular Ca^{2+} response after treatment with 500 μM succinate (live staining with Fluo-4AM). Data are % of responsive cells (± SEM) from n≥ 2 experiments. Representative images of *Sucnr1*$^{-/-}$ NSCs (baseline and during stimulation) are pseudocolored with red/blue according to high/low fluorescence intensity.

Scale bars: 50 μm (C), 25 μm (G). *p≤ 0.05 and **p≤ 0.01, *vs.* NSCs; ND: not detected.

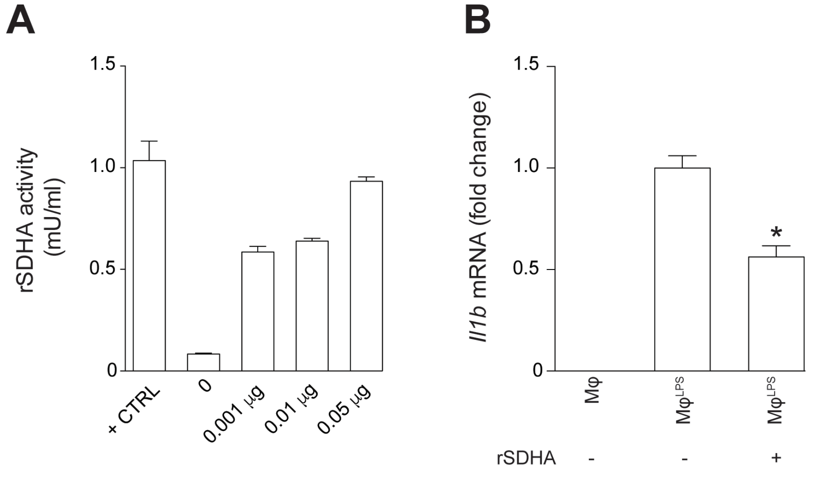

Peruzzotti-Jametti et al. Figure S5

Figure S5. Related to Figures 4 and 5. Anti-inflammatory effect of recombinant SDHA on type 1 inflammatory Mφ *in vitro*

(A) Activity of increasing concentrations of mouse recombinant complex subunit A (rSDHA) compared to manufacturer's positive control (+ CTRL).

Data are mean numbers (± SEM) from n= 3 independent replicates per condition.

(B) *Il1b* expression relative to *Actb* in Mφ^{LPS} treated with 0.05 μg/ml of mouse rSDHA. Data are mean fold change *vs.* Mφ^{LPS} (± SEM) from n≥ 3 independent experiments per condition.

*p≤ 0.05, *vs.* Mφ^{LPS}.

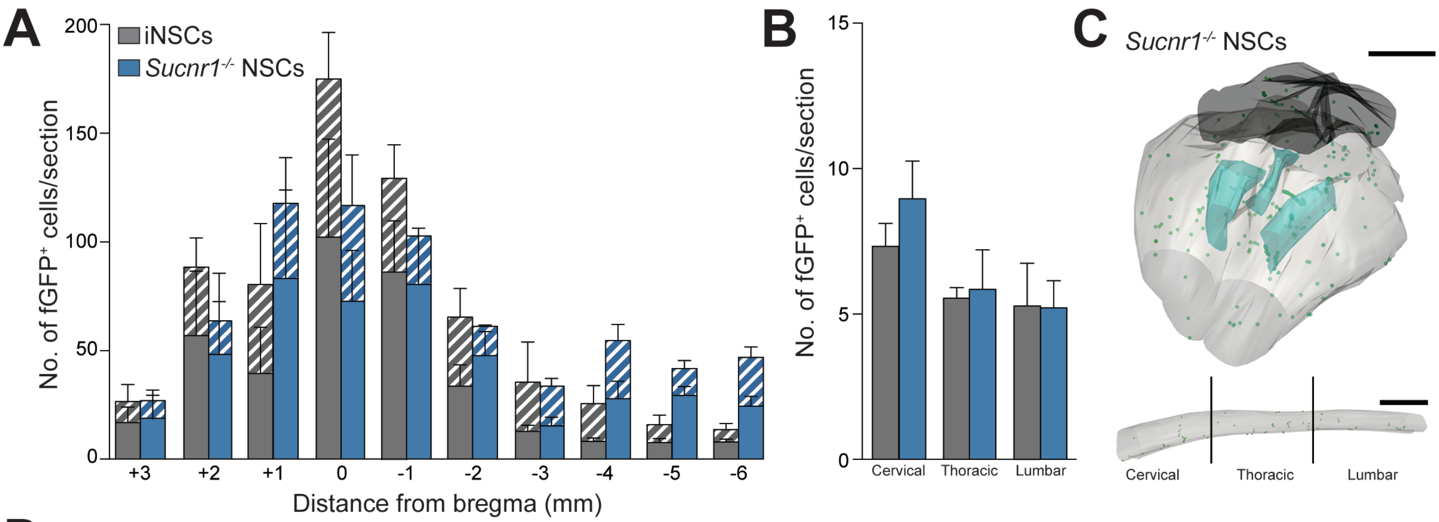

D

NSCs			*Sucnr1-/- NSCs*		
**Mean ± SEM**	**Brain**	**Spinal Cord**	**Mean ± SEM**	**Brain**	**Spinal Cord**
**No. of fGFP+ cells**	26,080 ± 7,350	727 ± 71	**No. of fGFP+ cells**	26,740 ± 5,871	801± 92
**% Nestin+/fGFP+**	2.30 ± 0.25	1.42 ± 0.27	**% Nestin+/fGFP+**	3.11 ± 1.19	2.24 ± 0.33
**% Ki67+/fGFP+**	0.62 ± 0.28	0.16 ± 0.16	**% Ki67+/fGFP+**	1.12 ± 0.55	0.46 ± 0.27
**% NeuN+/fGFP+**	0.97 ± 0.40	ND	**% NeuN+/fGFP+**	0.68 ± 0.23	ND
**% GFAP+/fGFP+**	23.64 ± 2.13	31.54 ± 0.49	**% GFAP+/fGFP+**	21.67 ± 2.59	30.17 ± 2.07
**% OLIG2+/fGFP+**	4.54 ± 0.69	1.08 ± 0.45	**% OLIG2+/fGFP+**	6.09 ± 1.69	0.43 ± 0.28
**% DCX+/fGFP+**	0.94 ± 0.20	ND	**% DCX+/fGFP+**	1.94 ± 0.50	ND

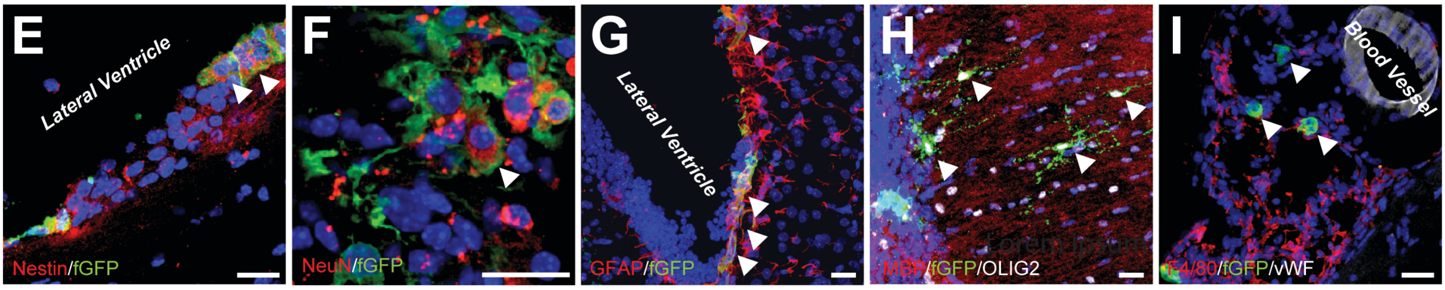

Peruzzotti-Jametti et al. Figure S6

Figure S6. Related to Figure 6. Distribution and differentiation of icv-injected *Sucnr1$^{-/-}$* NSCs at 30 dpt

(A-B) Stereology-based quantification of whole brain (A) and spinal cord (B) of fGFP^{+} *Sucnr1$^{-/-}$* NSCs (light blue bars) or fGFP^{+} NSCs (grey bars) injected icv in EAE mice at peak of disease. Solid bars in A are data from the left-brain

hemisphere, while wide upward diagonal bars are from data of the right brain hemisphere. Data are mean numbers (± SEM) from n≥ 4 mice/group.

(C) Representative 3D reconstructions of a brain and spinal cord from a *Sucnr1*$^{-/-}$ NSCs-treated EAE mouse (green dots indicate individual fGFP^{+} cells, contours of the ventricles are in light blue, those of the forebrain or spinal cord are in light grey, and those of the cerebellum are in dark grey).

(D) *In vivo* differentiation profile of transplanted fGFP^{+} *Sucnr1*$^{-/-}$ NSCs or fGFP^{+} NSCs at 30 dpt. Data are mean numbers (± SEM) from n≥ 4 mice/group.

(E-H) Representative images of fGFP^{+} *Sucnr1*$^{-/-}$ NSCs at 30 dpt expressing the neural marker Nestin (E, arrowheads), the mature neuronal marker NeuN (F, arrowhead), the astroglial lineage marker GFAP (G, arrowheads) or the oligodendroglial lineage marker OLIG2 (H, arrowheads).

(I) Confocal microscopy image of a perivascular area with several fGFP^{+} *Sucnr1*$^{-/-}$ NSCs (arrowheads) in juxtaposition with fGFP^{-}/F4/80^{+} MPs.

Nuclei in E-I are stained with DAPI (blue). Scale bars: 2 mm (C), 25 μm (E-I). ND: not detected.
