# Supplementary material for: Macrophage-Derived Extracellular Succinate Licenses Neural Stem Cells to Suppress Chronic Neuroinflammation
Source: Cell Stem Cell. 2018 Mar 1;22(3):355–368.e13. doi: 10.1016/j.stem.2018.01.020 (PMC5842147; doi:10.1016/j.stem.2018.01.020)
Supplement: Document S2. Article plus Supplemental Information [file mmc6.pdf]

Article

Cell Stem Cell

Macrophage-Derived Extracellular Succinate Licenses Neural Stem Cells to Suppress Chronic Neuroinflammation

Graphical Abstract

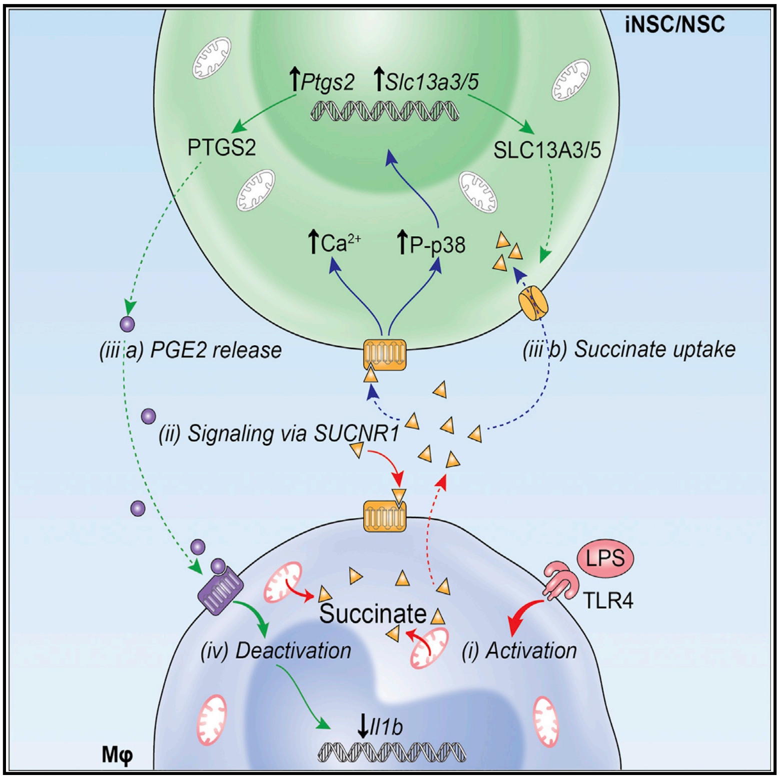

Highlights

- NSCs from somatic tissues or direct reprogramming equally repress neuroinflammation
- Extracellular succinate activates SUCNR1/GPR91 on NSCs
- Activated NSCs secrete PGE2 and scavenge succinate, thus reprogramming type 1 MPs
- *Sucnr1* mutant NSCs have reduced anti-inflammatory activity after transplantation

Authors

Luca Peruzzotti-Jametti, Joshua D. Bernstock, Nunzio Vicario, ..., Frank Edenhofer, Christian Frezza, Stefano Pluchino

Correspondence

lp429@cam.ac.uk (L.P.-J.), frank.edenhofer@uibk.ac.at (F.E.), cf366@mrc-cu.cam.ac.uk (C.F.), spp24@cam.ac.uk (S.P.)

In Brief

Peruzzotti-Jametti et al. demonstrate that somatic and directly induced brain stem cells injected into the cerebrospinal fluid of mice with experimental multiple sclerosis ameliorate chronic neuroinflammation. Grafted stem cells use SUCNR1 to decrease the inflammatory metabolite succinate, thus inducing a metabolic switch in endogenous macrophages and microglia toward an anti-inflammatory phenotype.

Peruzzotti-Jametti et al., 2018, Cell Stem Cell 22, 355–368
March 1, 2018 Crown Copyright © 2018 Published by Elsevier Inc.
https://doi.org/10.1016/j.stem.2018.01.020

CellPress

Macrophage-Derived Extracellular Succinate Licenses Neural Stem Cells to Suppress Chronic Neuroinflammation

Luca Peruzzotti-Jametti,[1,*] Joshua D. Bernstock,[1,2,8] Nunzio Vicario,[1,8] Ana S.H. Costa,[3] Chee Keong Kwok,[4] Tommaso Leonardi,[1] Lee M. Booty,[5] Iacopo Bicci,[1] Beatrice Balzarotti,[1] Giulio Volpe,[1] Giulia Mallucci,[1] Giulia Manferrari,[1] Matteo Donegà,[1] Nunzio Iraci,[1,7] Alice Braga,[1] John M. Hallenbeck,[2] Michael P. Murphy,[5] Frank Edenhofer,[4,6,*] Christian Frezza,[3,*] and Stefano Pluchino[1,9,*]

[1]Department of Clinical Neurosciences and NIHR Biomedical Research Centre, University of Cambridge, Cambridge, UK
[2]Stroke Branch, National Institute of Neurological Disorders and Stroke, NIH (NINDS/NIH), Bethesda, MD, USA
[3]MRC Cancer Unit, Hutchison/MRC Research Centre, University of Cambridge, Cambridge, UK
[4]Institute of Anatomy and Cell Biology, University of Würzburg, Würzburg, Germany
[5]MRC Mitochondrial Biology Unit, Hills Road, University of Cambridge, Cambridge, UK
[6]Institute of Molecular Biology and CMBI, Genomics, Stem Cell Biology and Regenerative Medicine, Leopold-Franzens-University Innsbruck, Innsbruck, Austria
[7]Department of Biomedical and Biotechnological Sciences (BIOMETEC), University of Catania, Via S. Sofia 97, Catania 95125, Italy
[8]These authors contributed equally
[9]Lead Contact
*Correspondence: lp429@cam.ac.uk (L.P.-J.), frank.edenhofer@uibk.ac.at (F.E.), cf366@mrc-cu.cam.ac.uk (C.F.), spp24@cam.ac.uk (S.P.)
https://doi.org/10.1016/j.stem.2018.01.020

SUMMARY

Neural stem cell (NSC) transplantation can influence immune responses and suppress inflammation in the CNS. Metabolites, such as succinate, modulate the phenotype and function of immune cells, but whether and how NSCs are also activated by such immunometabolites to control immunoreactivity and inflammatory responses is unclear. Here, we show that transplanted somatic and directly induced NSCs ameliorate chronic CNS inflammation by reducing succinate levels in the cerebrospinal fluid, thereby decreasing mononuclear phagocyte (MP) infiltration and secondary CNS damage. Inflammatory MPs release succinate, which activates succinate receptor 1 (SUCNR1)/GPR91 on NSCs, leading them to secrete prostaglandin E2 and scavenge extracellular succinate with consequential anti-inflammatory effects. Thus, our work reveals an unexpected role for the succinate-SUCNR1 axis in somatic and directly induced NSCs, which controls the response of stem cells to inflammatory metabolic signals released by type 1 MPs in the chronically inflamed brain.

INTRODUCTION

Advances in stem cell biology have raised hopes that diseases of the CNS may be ameliorated by non-hematopoietic stem cell medicines (Martino and Pluchino, 2006). We have provided compelling evidence that the transplantation of somatic neural stem cells (NSCs) improves the clinico-pathological features of animal models of inflammatory CNS disorders. Beyond the structural replacement of injured CNS cells, our work has shown that transplanted NSCs engage in complex stem cell graft-to-host communication programs, overall leading to trophic support and modulation of adaptive and innate immune responses (Bacigaluppi et al., 2009, 2016; Pluchino and Cossetti, 2013; Pluchino et al., 2005, 2009b). Specifically, NSC transplants reduce the burden of inflammation at site of injury (Pluchino et al., 2005, 2009a), decrease the number of type 1 inflammatory mononuclear phagocytes (MPs) (Cusimano et al., 2012), and promote the healing of the injured CNS via yet poorly characterized mechanisms.

However, the clinical translation of experimental NSC therapies is still limited by the sources from which human NSCs (hNSCs) are derived (Anderson et al., 2017), the intrinsic immunogenicity of allogeneic hNSC lines (Ramos-Zúñiga et al., 2012; Rice et al., 2013), and the stability of the so-called "intended clinical cell lot" (Anderson et al., 2017; Wright et al., 2006). Autologous and stably expandable directly induced NSCs (iNSCs) from patients' dermal fibroblasts are emerging as a valid alternative to NSC therapies (Lu et al., 2013; Meyer et al., 2015; Thier et al., 2012). The direct reprogramming into iNSCs avoids the laborious progression through a pluripotent state and subsequent differentiation into desired lineages described for induced pluripotent stem cell (iPSC) technology (Meyer et al., 2015; Thier et al., 2012). Therefore, making stably expandable iNSCs from somatic cells represents the most feasible way of obtaining autologous brain stem cells for downstream clinical applications (Wörsdörfer et al., 2013). However, the efficacy of directly reprogrammed iNSCs in treating inflammatory CNS disorders has not yet been tested.

In progressive forms of multiple sclerosis (MS), chronic CNS inflammation is sustained by widespread activation of MPs that include both CNS resident microglia and monocyte-derived infiltrating macrophages (Mallucci et al., 2015). MPs are found in

This is an open access article under the CC BY license (http://creativecommons.org/licenses/by/4.0/).

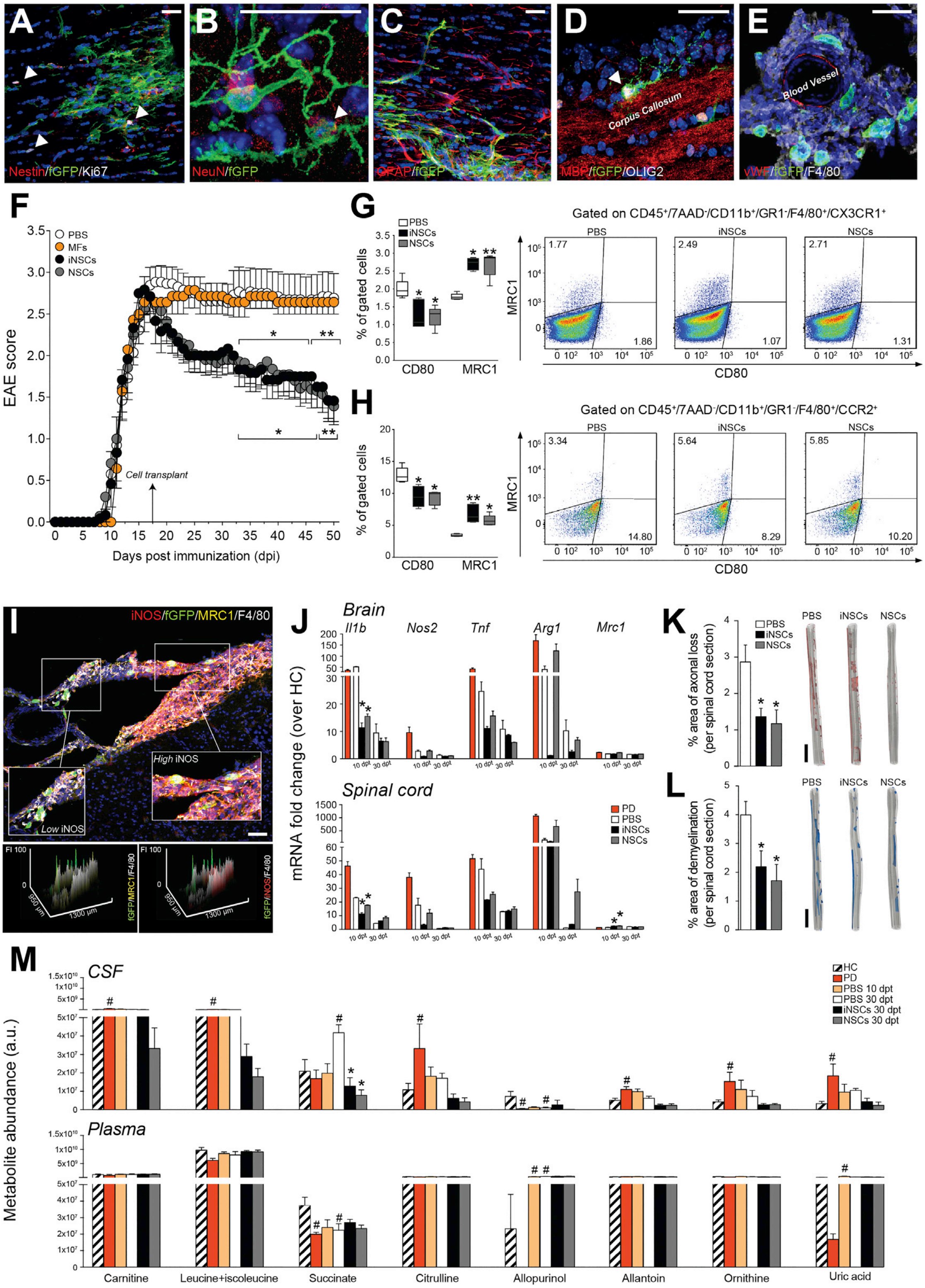

Figure 1. NSCs Transplantation Ameliorates Chronic Neuroinflammation and Reduces Succinate Levels in the CSF of EAE Mice

(A–D) Representative images of fGFP$^+$ iNSCs at 30 dpt expressing the proliferation marker Ki67 (A, arrowheads) and the neural marker Nestin (A), the mature neuronal marker NeuN (B, arrowhead), the astroglial lineage marker GFAP (C), or the oligodendroglial lineage marker OLIG2 (D, arrowhead).

(E) Confocal microscopy image of a perivascular area with several fGFP$^+$ iNSCs in juxtaposition to fGFP$^-$/F4/80$^+$ MPs. Nuclei in (A)–(E) are stained with DAPI (blue).

(legend continued on next page)

gray matter lesions, close to degenerating neurites and neuronal cell bodies (Peterson et al., 2001), and in white matter lesions, where the external rim of activated microglia is associated with chronic tissue damage (Bramow et al., 2010; Prineas et al., 2001). Areas of normal-appearing white matter are also characterized by MP accumulation, which leads to the formation of microglial nodules that drive disease pathology irrespective of concomitant T cell activation (Moll et al., 2011). The detrimental role of chronic MP-driven inflammation in progressive MS is also supported by evidence in animal disease models, where its overall burden correlates with impaired neuronal function (Planche et al., 2017), brain atrophy (Tambalo et al., 2015), and reduced regenerative responses (Jiang et al., 2014).

Activation of MPs by pro-inflammatory stimuli causes a metabolic switch toward glycolysis and reduced oxidative phosphorylation (OXPHOS) (Kelly and O'Neill, 2015). Recent evidence suggests that, within this metabolic rewiring, type 1 inflammatory MPs accumulate succinate, with important pathophysiological implications (Tannahill et al., 2013). Intracellular succinate inhibits the activity of prolyl hydroxylases enzymes (PHDs), thereby stabilizing hypoxia responsive element (HIF)-1α and inducing the transcription of interleukin (IL)-1β (Tannahill et al., 2013). Furthermore, oxidation of succinate by succinate dehydrogenase (SDH) repurposes mitochondria from ATP synthesis to reactive oxygen species (ROS) production as additional pro-inflammatory signal (Mills et al., 2016). Type 1 inflammatory MPs also release succinate extracellularly and upregulate its cognate succinate receptor 1 (SUCNR1), a G-protein-coupled receptor (also known as GPR91), which functions as autocrine and paracrine sensor to enhance IL-1β production (Littlewood-Evans et al., 2016).

As such, metabolism is emerging as an important therapeutic target to modulate the activation of both macrophages (Kelly and O'Neill, 2015) and microglia (Orihuela et al., 2016), and succinate-related pathways have key immune modulatory functions for acute and chronic inflammatory diseases (Ryu et al., 2003; Tannahill et al., 2015).

Given the established immune modulatory properties of NSCs (Pluchino and Cossetti, 2013), we hypothesized that NSCs may exert their therapeutic effects in chronic neuroinflammation by modulating MP metabolism toward reduction of secondary CNS damage.

In this work, we investigated the molecular mechanisms that underpin the capacity of somatic and directly induced NSCs to counteract the metabolic changes of type 1 inflammatory MPs both *in vivo* and *in vitro*. We show that transplanted iNSCs and NSCs are functionally equivalent in ameliorating chronic neuroinflammation in mice with experimental autoimmune encephalomyelitis (EAE). Transplanted iNSCs/NSCs switch in the activation profile of CNS-resident microglia and monocyte-derived infiltrating macrophages toward an anti-inflammatory phenotype, as well as reduce the levels of the immunometabolite succinate in the cerebrospinal fluid (CSF). iNSCs/NSCs also decrease extracellular succinate released by type 1 inflammatory MPs to reprogram their metabolism toward OXPHOS *in vitro*. Mechanistically, we show that succinate secreted by type 1 MPs elicits in iNSCs/NSCs a signaling cascade downstream SUCNR1, which enables their anti-inflammatory activity. This succinate-licensed anti-inflammatory function of iNSCs/NSCs is mediated by the secretion of prostaglandin (PG) E2, as well as by considerable scavenging of extracellular succinate. Loss of *Sucnr1* function in NSCs leads to significantly reduced anti-inflammatory activities *in vitro* and *in vivo* after transplantation in EAE.

Our study uncovers a succinate-SUCNR1 axis that clarifies how NSCs respond to inflammatory metabolic signals to inhibit the activation of type 1 MPs in chronic neuroinflammation.

RESULTS

NSC Transplantation Ameliorates Chronic Neuroinflammation and Is Coupled with Reduction of the Immunometabolite Succinate in the Cerebrospinal Fluid

We first assessed the effects of the intracerebroventricular (icv) transplantation at peak of disease (PD) of iNSCs or NSCs in mice with MOG35-55-induced chronic EAE and compared it to PBS-treated control EAE mice. Prior to transplantation, iNSCs and NSCs were expanded, characterized (Figure S1), and labeled with farnesylated (f)GFP *in vitro*. At 30 days post-transplantation (dpt), iNSC and NSC transplants survived, distributed, and integrated within the EAE brain and spinal cord (Figure S2). Only a minority of retrieved fGFP$^+$ cells (iNSCs: 2.1% ± 0.9%; NSCs: 1.7% ± 0.1%) were proliferating (Figure 1A) or expressing neuronal (Figure 1B), astroglial (Figure 1C), or oligodendroglial

(F) Behavioral outcome of iNSCs/NSCs-transplanted EAE mice. Data are mean EAE score (±SEM) from n $\geq$ 7 mice/group over n = 2 independent experiments. EAE mice injected icv with mouse fibroblasts (MFs) or PBS were used as controls.

(G and H) Flow-cytometry-based *ex vivo* quantification of the expression levels of type 1 inflammatory (CD80) and anti-inflammatory (MRC1) markers in CX3CR1$^+$ microglial cells (G) and CCR2$^+$ monocyte-derived infiltrating macrophages (H) from the CNS of iNSC- and NSC-transplanted EAE mice at 30 dpt. Quantitative data are shown on the left, whereas representative density plots are shown on the right. Data are min to max % of marker-positive cells from n $\geq$ 4 pools of mice/group.

(I) Representative confocal microscopy image and comparative histograms of a perivascular area with several fGFP$^+$ iNSCs in juxtaposition to F4/80$^+$ MPs. Low iNOS and prevalent MRC1 expression is detected in F4/80$^+$ MPs close to fGFP$^+$ iNSCs (inset on the left), whereas high iNOS expression is observed in the remaining MP infiltrate (inset on the right). Nuclei are stained with DAPI.

(J) Expression levels (qRT-PCR) of pro- and anti-inflammatory genes in the brain and spinal cord of EAE mice. Data are mean fold change over HC from n $\geq$ 3 mice/group.

(K and L) Quantification and representative 3D reconstructions of spinal cord damage in iNSC- and NSC-transplanted EAE mice. Data are mean % of Bielschowsky negative-stained axonal loss (K) or Luxol fast blue (LFB) negative-stained demyelinated (L) areas/spinal cord section (±SEM) from n $\geq$ 5 mice/group over n = 2 independent experiments.

(M) Levels of CSF metabolites significantly changed during EAE (versus HC). Corresponding levels in matched plasma samples are also shown. Data are mean a.u. (±SEM) from n $\geq$ 3 mice/group.

The scale bars represent 25 μm (A–E), 50 μm (I), and 2 mm (K and L). *p $\leq$ 0.05 and **p $\leq$ 0.01 versus PBS; $^{\#}$p $\leq$ 0.05 versus HC; dpt, days post-transplantation; FI, fluorescence intensity; HC, healthy controls; PD, peak of disease. See also Figures S1, S2, and S3 and Table S1.

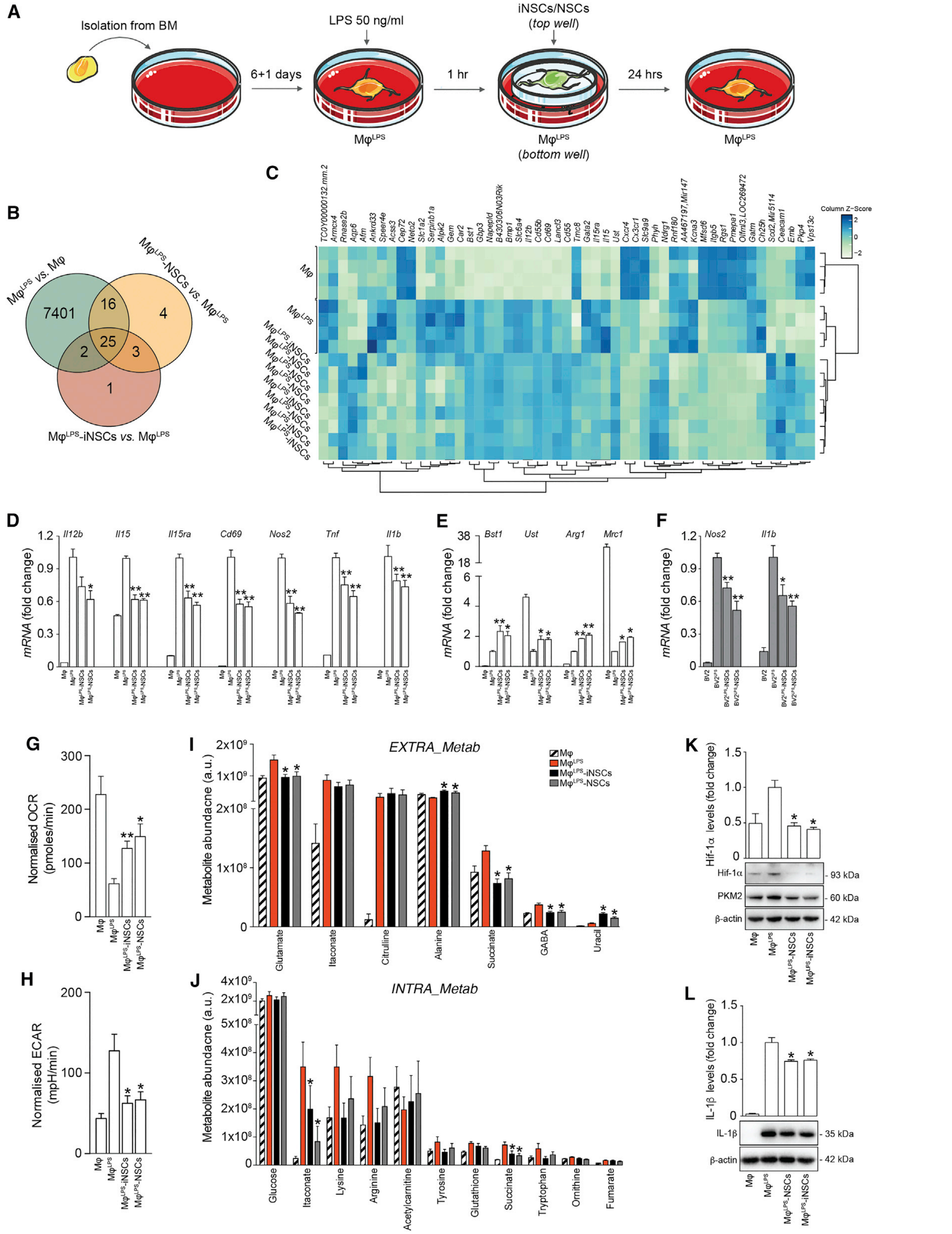

(legend on next page)

(Figure 1D) lineage markers (Figure S2). The majority (~75%) of iNSCs surviving to transplantation were found instead not to be expressing any of the neural lineage markers tested and localizing around meningeal perivascular niche-like areas close to F4/80$^+$ endogenous MPs (Figure 1E), as observed in somatic NSC grafts (Cusimano et al., 2012; Pluchino et al., 2003). The transplantation of iNSCs induced a significant and long-lasting (up to 90 dpt) amelioration of EAE scores, which started from 15 to 20 dpt onward (Figures 1F and S2). Functional recovery was also confirmed by computer-assisted automated gait analysis (Figure S2). Overall, icv-transplanted iNSCs were safe and led to behavioral and pathological recovery.

We then analyzed the composition of CNS inflammatory infiltrates via *ex vivo* flow cytometry in iNSC- and NSC-transplanted versus PBS-treated control EAE mice. The transplantation of iNSCs or NSCs had no effects on the fraction of CNS-infiltrating T cells, B cells, and total MPs, as well as in that of CD3$^+$/CD4$^+$ T cell subsets (including Th1, Th2, Treg, ThGM-CSF, and Th17 subsets) at 30 dpt (Figure S3). Instead, iNSC- or NSC-transplanted EAE mice showed a significant switch in the activation profile of CX3CR1$^+$ cells with ~1.5-fold decrease of the CD80$^+$ type 1 inflammatory microglia and parallel increase of the MRC1$^+$ anti-inflammatory microglia (Figure 1G). Likewise, CNS-infiltrating (monocyte-derived) CCR2$^+$ macrophages from iNSC- or NSC-transplanted EAE mice underwent significant phenotype switch with ~1.3-fold decrease of the CD80$^+$ type 1 inflammatory macrophages and parallel ~1.8-fold increase of the MRC1$^+$ anti-inflammatory macrophages (Figure 1H). This effect was accompanied by a significant reduction of the expression of the type 1 inflammatory MP marker inducible nitric oxide synthase (iNOS) by F4/80$^+$ MPs *in vivo* (Figures 1I and S3).

We then analyzed the expression levels of the main pro- and anti-inflammatory genes in the whole CNS. iNSC- and NSC-transplanted EAE mice both exhibited significantly reduced levels of *interleukin-1 beta* (*Il1b*) in the brain and spinal cord and increased levels of *mannose receptor C type 1* (*Mrc1*) in the spinal cord, both at 10 dpt (Figure 1J).

We found no significant differences in blood-brain barrier (BBB) permeability at 30 dpt when comparing iNSC-/NSC-transplanted with PBS-treated control EAE mice (Figure S3).

Finally, iNSC- and NSC-transplanted EAE mice accumulated significantly reduced axonal loss (Figure 1K) and demyelination (Figure 1L) in the spinal cord.

Given the established importance of metabolism in regulating the phenotype and function of MPs, we investigated whether NSC transplants affected the neuroinflammatory metabolic microenvironment. To this end, we performed an untargeted metabolic profiling of polar metabolites by liquid chromatography coupled to mass spectrometry (LC-MS) of matched CSF and plasma samples (Table S1). PBS-treated control EAE mice showed a significant increase of several CSF (but not plasma) metabolites, among which succinate only peaked at 45 days post-immunization (dpi) (corresponding to 30 dpt; Figure 1M). EAE mice not subjected to surgery also showed a significant increased succinate only in the CSF at 45 dpi (versus healthy control mice), which was not different from the levels of succinate in the CSF PBS-treated control EAE mice (Figure S3).

Whereas we did not detect any significant change in plasma metabolite levels between iNSC/NSC-transplanted and PBS-treated control EAE mice (Table S1), we found that the transplantation of iNSCs or NSCs led to a significant drop in CSF succinate at 30 dpt (Figure 1M; Table S1).

Further, we found no significant differences in CSF succinate when comparing PBS-treated EAE mice versus EAE mice injected icv with mouse fibroblasts (MFs) as control cells (Figure S3).

Thus, iNSCs and NSCs directly injected into the EAE CNS induce a specific phenotype switch of MPs, which is associated with reduction of the immunometabolite succinate in the CSF only and amelioration of chronic neuroinflammation.

NSCs Reduce Succinate Levels and Reprogram the Metabolism of Type 1 Inflammatory Mφ *In Vitro*

We then investigated the molecular mechanisms through which iNSCs/NSCs display anti-inflammatory activities on type 1 MPs, using an *in vitro* system that recapitulates the interactions between MPs and iNSCs/NSCs. Naive bone-marrow-derived macrophages (Mφ) were polarized into a type 1 inflammatory phenotype with LPS (Mφ^{LPS}), as described (Tannahill et al., 2013). Mφ^{LPS} were then co-cultured with iNSCs (Mφ^{LPS}-iNSCs) or NSCs (Mφ^{LPS}-NSCs) in a trans-well system that avoids cell-to-cell contacts (Figure 2A). Unpolarized Mφ were used as controls.

Microarray gene expression profiling showed significant transcriptional changes in Mφ^{LPS} with 7,401 genes affected (versus Mφ; adjusted p value < 0.1; Figure 2B; Table S2) and 51 genes differentially expressed in Mφ^{LPS}-iNSCs or Mφ^{LPS}-NSCs (versus

Figure 2. NSCs Reduce Succinate Levels and Reprogram the Metabolism of Type 1 Pro-inflammatory Mφ toward Oxidative Phosphorylation *In Vitro*

(A) Experimental setup for *in vitro* Mφ^{LPS} co-cultures with iNSCs/NSCs.

(B and C) Gene expression microarrays of Mφ^{LPS}-iNSCs/NSCs. (B) Venn diagram of differentially expressed genes (adjusted p value < 0.1). (C) Heatmap of genes differentially expressed (adjusted p value < 0.1) in Mφ^{LPS}-iNSCs or Mφ^{LPS}-NSCs.

(D and E) qRT-PCR independent validation of differentially expressed inflammatory genes as in (C). (D) Expression of genes related to type 1 inflammatory (E) and anti-inflammatory Mφ phenotypes relative to *Actb*. Data are mean fold change (±SEM) versus Mφ^{LPS} from n ≥ 3 independent replicates per condition.

(F) qRT-PCR of BV2^{LPS}-iNSCs/NSCs (±SEM) from n ≥ 3 independent experiments per condition. BV2 and BV2^{LPS} are shown as controls.

(G and H) Extracellular flux (XF) assay of the oxygen consumption rate (OCR) (G) and extracellular acidification rate (ECAR) (H) in Mφ^{LPS}-iNSCs/NSCs. Data were normalized on total protein content and are expressed as mean values (±SEM) from n ≥ 3 independent experiments per condition.

(I and J) Levels of significantly changed extracellular (*EXTRA_Metab*, I) and intracellular (*INTRA_Metab*, J) metabolites in Mφ^{LPS} versus Mφ at 25 hr. Data are mean a.u. (±SEM) from n ≥ 2 independent experiments per condition.

(K and L) Hif-1α (K), PKM2 (K), and IL-1β (L) expression levels relative to β-actin. Data are mean fold change versus Mφ^{LPS} (±SEM) from n ≥ 3 independent experiments per condition.

*p ≤ 0.05 and **p ≤ 0.01 versus Mφ^{LPS}. See also Tables S2 and S3.

Mφ^{LPS}; adjusted p value < 0.1; Figures 2B and 2C; Table S2). This latter set of genes was enriched in biological processes related to positive regulation of leukocyte activation (GO: 0002696), myeloid leukocyte differentiation (GO: 0002761), and immune system processes (GO: 0002376). Independent qRT-PCR validation of selected Mφ pro-inflammatory genes confirmed significant downregulation of the expression levels of *Il12b*, *Il15*, *Il15ra*, and *Cd69*, as well as the classical inflammatory genes *Nos2*, *tumor necrosis factor* (*Tnf*), and *Il1b* in Mφ^{LPS}-iNSCs and Mφ^{LPS}-NSCs (versus Mφ^{LPS}; Figure 2D). This effect was coupled with the concomitant upregulation of the expression levels of genes associated with an anti-inflammatory Mφ phenotype, such as uronyl-2-sulfotransferase (*Ust*) and *bone marrow stromal cell antigen 1* (*Bst1*) (Al-Shabany et al., 2016; Martinez et al., 2015), as well as *arginase 1* (*Arg1*) and *Mrc1* (versus Mφ^{LPS}; Figure 2E). When iNSCs/NSCs were co-cultured with lipopolysaccharide (LPS)-activated mouse BV2 microglial cells as before, significant reduction of the expression levels of the pro-inflammatory genes *Nos2* and *Il1b* was also observed (Figure 2F).

To link gene expression profiles with functional metabolic states, we assessed the basal oxygen consumption rate (OCR) and extracellular acidification rate (ECAR) of Mφ^{LPS} as readouts of their tricarboxylic acid (TCA) cycle and glycolytic activities, respectively. We found a significant reduction of OCR and a significant increase of ECAR in Mφ^{LPS} (versus Mφs). Instead, Mφ^{LPS}-iNSCs and Mφ^{LPS}-NSCs underwent significant restoration of both OCR and ECAR values (versus Mφ^{LPS}; Figures 2G and 2H), as observed in Mφ switching to an anti-inflammatory phenotype (O'Neill and Pearce, 2016).

In an effort to clarify the metabolic determinants of these anti-inflammatory effects, we performed an untargeted LC-MS analysis of the extracellular and intracellular small-molecule metabolite content of Mφ^{LPS}. As expected, LPS stimulation profoundly changed the extracellular and intracellular metabolic milieu of Mφ (Mφ^{LPS}) (versus Mφ; Table S3). In co-cultures, Mφ^{LPS}-iNSCs and Mφ^{LPS}-NSCs both showed significant reduction of extracellular glutamate, GABA, and succinate (versus Mφ^{LPS}; Figure 2I; Table S3). Furthermore, Mφ^{LPS}-iNSCs and Mφ^{LPS}-NSCs also displayed a significant reduction of intracellular succinate and itaconate (versus Mφ^{LPS}; Figure 2J; Table S3).

Consistent with the reduction of succinate levels, we found that Mφ^{LPS}-iNSCs and Mφ^{LPS}-NSCs exhibited significantly reduced levels of HIF-1α, of the upstream protein pyruvate kinase isozyme M2 (PKM2) (Palsson-McDermott et al., 2015; Figure 2K), as well as of IL-1β (versus Mφ^{LPS}; Figure 2L).

Altogether, these *in vitro* data provide evidence that iNSCs/NSCs reduce the accumulation of both intracellular and extracellular succinate in co-cultures with type 1 inflammatory MPs, reprogramming them toward an OXPHOS anti-inflammatory phenotype.

Succinate Signals via SUCNR1/GPR91 in Mouse and Human NSCs

Given the importance of succinate as immunometabolic signal, we investigated whether succinate released by type 1 pro-inflammatory MPs could regulate the activity of surrounding cells *in situ*, including that of transplanted iNSCs/NSCs.

We found that transplanted iNSCs/NSCs detected in proximity to meningeal perivascular areas (Figures 3A and 3B) and F4/80$^+$ MPs (Figure 3C) expressed SUCNR1 *in vivo* in the CNS. SUCNR1 was also expressed at protein level on both iNSCs and NSCs *in vitro*, but not in MFs (Figure 3D).

To further assess whether SUCNR1 in iNSCs/NSCs was functionally activated by succinate, we investigated its downstream signaling cascade *in vitro*. When exposed to succinate (Figure 3E; Rubic et al., 2008), 34.2% (±7.4%) of iNSCs and 31.7% (±6.5%) of NSCs showed a release of intracellular calcium stores (Figures S4 and 3F). This response was followed by a significant upregulation of the phospho-p38 mitogen-activated protein kinase (Figure 3G), indicative of its activation. We confirmed the expression of *SUCNR1* and SUCNR1 also in human fetal NSCs (hNSCs) and human iNSCs (hiNSCs) (Figures 3H and 3I). As in mouse iNSCs, succinate-dependent p38 signaling was evoked in hiNSCs, but not in hiNSCs pre-treated with the selective SUCNR1 inhibitor *4c* (Figure 3J).

Thus, mouse and human iNSCs and NSCs express functional SUCNR1, which induces a signaling pathway downstream of its stimulation with the immunometabolite succinate.

SUCNR1 Stimulation Initiates the Secretion of Prostaglandin E2 by NSCs

To clarify the functional consequences of SUCNR1 signaling in NSCs, we generated NSCs from mice lacking *Sucnr1* (*Sucnr1*$^{-/-}$ NSCs) (Rubic et al., 2008; Figure S4). Compared to control NSCs, *Sucnr1*$^{-/-}$ NSCs showed similar growth curves and differentiation *in vitro* (Figure S4). However, when exposed to succinate at different time points and concentrations, *Sucnr1*$^{-/-}$ NSCs showed no upregulation of phospho-p38 (Figure S4). Stimulation with glutamate or ATP + thapsigargin induced in *Sucnr1*$^{-/-}$ NSCs a calcium response similar to that of control NSCs (Figure S4). On the contrary, succinate treatment did not elicit release of calcium from intracellular stores (Figure S4), which indicated a defective SUCNR1 signaling in *Sucnr1*$^{-/-}$ NSCs.

We then performed a gene expression profiling microarray following treatment with succinate in control NSCs and *Sucnr1*$^{-/-}$ NSCs (Table S4). We found that *prostaglandin-endoperoxide synthase 2* (*Ptgs2*), the key enzyme in PG biosynthesis encoding the inducible PTGS2, was the most upregulated gene in succinate-stimulated control NSCs (log_2 fold change 1.05), but not in succinate-stimulated *Sucnr1*$^{-/-}$ NSCs (log_2 fold change −0.43; Figure 4A). We validated these results on *Ptgs2* by qRT-PCR, confirming that its expression levels were significantly upregulated (2.1- to 2.7-fold change) in succinate-stimulated iNSCs and NSCs, whereas they were not in succinate-treated *Sucnr1*$^{-/-}$ NSCs (Figure 4B).

Given the role of PGE2 as regulator of the immunosuppressive effects of mesenchymal stem cells (MSCs) (Vasandan et al., 2016; Yañez et al., 2010), we tested its accumulation in tissue culture media from iNSCs, NSCs, and *Sucnr1*$^{-/-}$ NSCs after stimulation with succinate. iNSCs and NSCs, but not *Sucnr1*$^{-/-}$ NSCs, showed significant (>2.5-fold) increase of their basal release of PGE2 as early as 30 min after succinate. This succinate-induced effect was abolished by pre-treatment with the irreversible PTGS2 blocker SC-58125 (Figure 4C). As in mouse iNSCs, exposure of hiNSCs to succinate elicited a significant

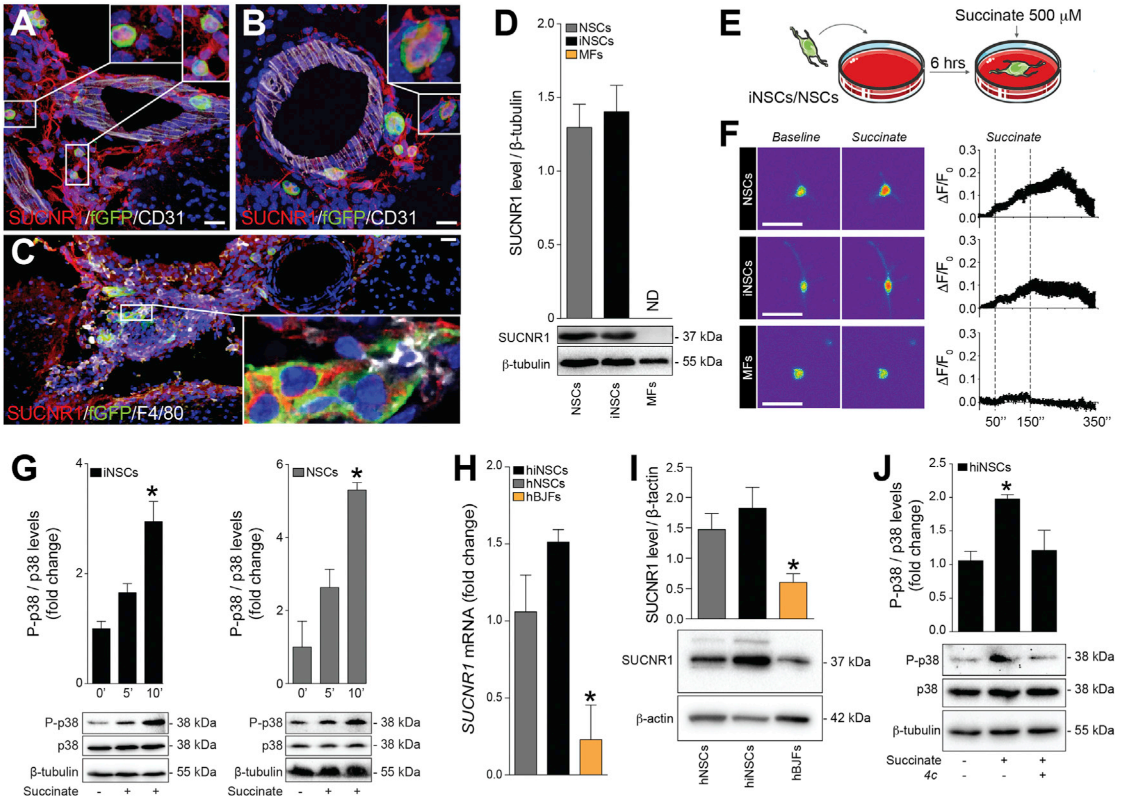

Figure 3. Succinate Signals via SUCNR1 in Mouse and Human NSCs

(A–C) Representative confocal microscopy images of meningeal perivascular areas with transplanted fGFP^{+} iNSCs (A) and NSCs (B) expressing SUCNR1 in the brain of a mouse with EAE. The image in (C) shows transplanted SUCNR1^{+} iNSCs in close vicinity to SUCNR1^{+}/F4/80^{+} MPs. Nuclei are stained with DAPI.

(D) SUCNR1 protein expression relative to β-tubulin *in vitro*. Data are shown as mean (±SEM) of n $\geq$ 3 independent replicates per condition.

(E) Experimental setup for succinate treatment of iNSCs/NSCs *in vitro*.

(F) Intracellular Ca^{2+} response after treatment with 500 μM succinate (live staining with Fluo-4AM). Representative images (baseline and during stimulation) are pseudocolored with red/blue according to high/low fluorescence intensity. Data are mean changes in fluorescence intensity as ΔF/F0 (±SEM) from n $\geq$ 3 experiments.

(G) Phospho-p38 MAPK (P-p38) and total p38 MAPK (p38) protein expression after succinate treatment. Data are P-p38/p38 expression relative to β-tubulin and expressed as mean fold change (±SEM) versus untreated cells over n $\geq$ 3 independent experiments per condition.

(H) qRT-PCR of *SUCNR1* basal expression in human cells. Data are normalized on *18S* and expressed as mean fold change (±SEM) versus NSCs from n $\geq$ 3 independent replicates per condition.

(I) Representative blot of SUNCR1 basal protein expression in human cells.

(J) P-p38 and p38 protein expression after stimulation with succinate ± pre-treatment with the irreversible inhibitor of the human SUCNR1 *4c*.

The scale bars represent 25 μm. *p $\leq$ 0.05 versus 0'. hBJFs, human BJ fibroblasts; ND, not detected. See also Figure S4.

increase of PGE2 concentrations in tissue culture media, whereas again pre-treatment with either SC-58125 or *4c* prevented its release (Figure 4D).

To further extend the relevance of these findings to co-cultures between NSCs and Mφ^{LPS}, we analyzed the levels of PGE2 in tissue culture media. We found that Mφ^{LPS}-NSCs accumulated higher levels of PGE2 compared to Mφ^{LPS}, whereas pre-treatment of co-cultured NSCs with SC-58125 significantly reduced PGE2 levels (Figure 4E). SC-58125 pre-treatment of NSCs was also coupled with a significant increase of *Il1b* expression in Mφ^{LPS} (Figure 4F) and with a reduction of OCR values indicative of a pro-inflammatory phenotype (Figure 4G). However, we noticed that NSCs pre-treated with SC-58125 retained some residual anti-inflammatory effects on Mφ^{LPS} compared to *Sucnr1*$^{-/-}$ NSCs (Figure 4F). On the contrary, *Sucnr1* loss of function in NSCs completely abolished their anti-inflammatory effects on Mφ^{LPS} (Figures 4F and 4G). We also show that the observed PGE2-dependent anti-inflammatory ability of NSCs is conserved and relevant for human NSCs.

As such, hiNSCs induced a significant reduction of *Il1b* expression in Mφ^{LPS} in co-cultures (Figure 4H), which was coupled with a restoration of OCR values (Figure 4I) and increased PGE2 levels in tissue culture media (Figure 4J). These effects were completely suppressed by pre-treatment of hiNSCs with the selective SUCNR1 inhibitor *4c* (Figures 4H–4J).

Thus, the activation of SUCNR1 signaling pathway in mouse and human NSCs triggers the release of PGE2 leading to anti-inflammatory effects on type 1 MPs.

However, inhibition experiments targeting either PTGS2 or SUCNR1 anticipate that additional SUCNR1-dependent—PGE2-independent—mechanisms are likely to play a key role in the anti-inflammatory effects of NSCs.

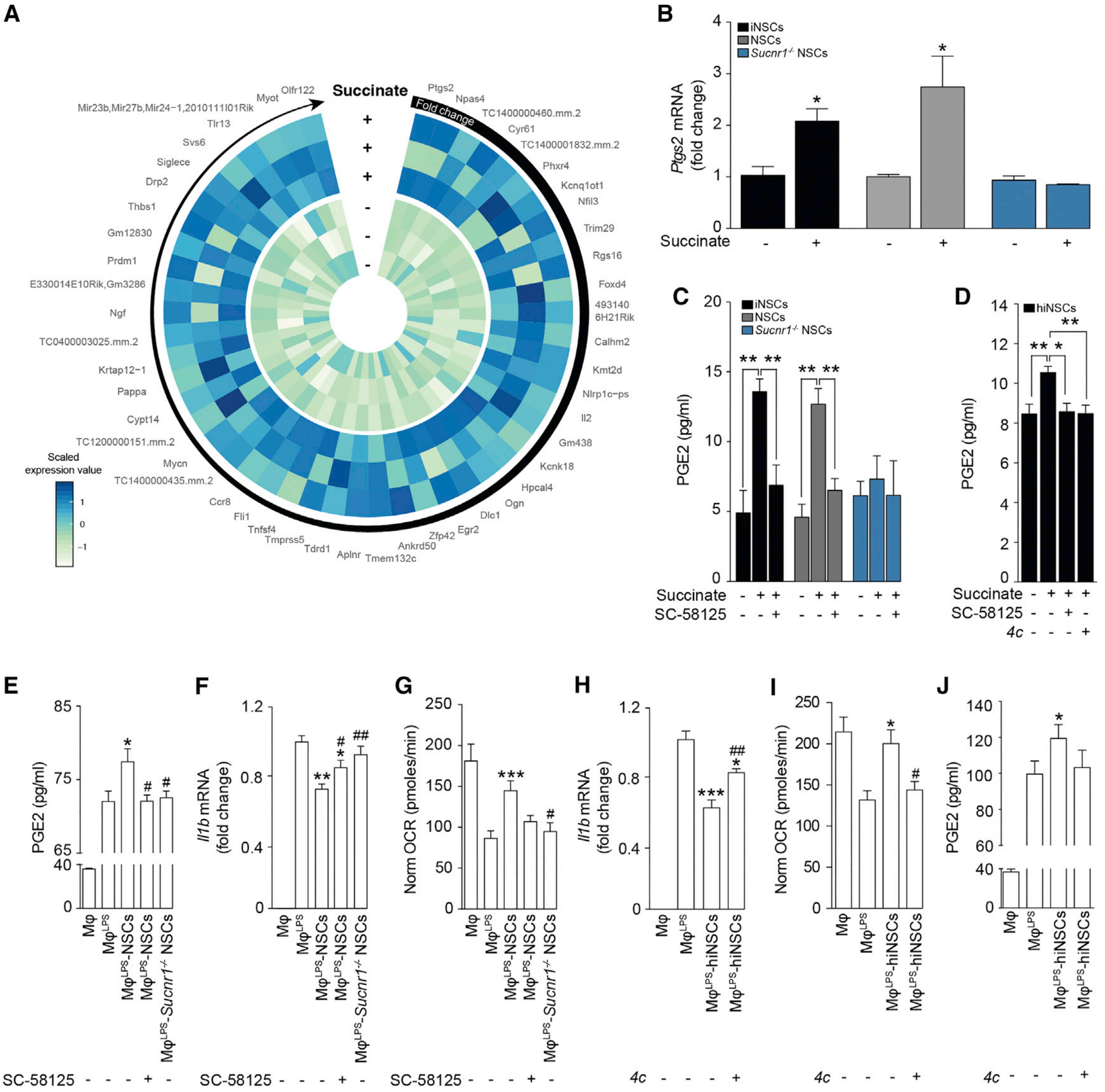

Figure 4. SUCNR1 Expression Is Necessary for the Anti-inflammatory Effect of NSCs on Type 1 Pro-inflammatory Mφ *In Vitro*

(A) Heatmap showing the microarray expression profile of the 50 most upregulated genes in NSCs after treatment with succinate. Data are shown as *Z* scores.

(B) qRT-PCR independent validation of *Ptgs2* expression as in (A). Data are calculated relative to *Actb* and shown as mean fold change (±SEM) versus untreated cells over n $\geq$ 3 independent experiments per condition.

(C) PGE2 secretion following 1 hr treatment with succinate ± pre-treatment with the selective PTGS2 blocker SC-58125. Data are mean values (±SEM) over n $\geq$ 3 independent experiments per condition.

(D) PGE2 secretion by hiNSCs treated with succinate ± pre-treatment with either SC-58125 or *4c*. Data are mean values (±SEM) over n $\geq$ 3 independent experiments per condition.

(E) PGE2 secretion in Mφ co-cultures. Data are mean values (±SEM) over n $\geq$ 3 independent experiments per condition.

(F) *Il1b* expression relative to *Actb* in Mφ co-cultures. Data are mean fold change versus $M\varphi^{LPS}$ (±SEM) from n $\geq$ 3 independent experiments per condition.

(G) XF assay of the OCR of Mφ as in (E) and (F). Data are normalized on total protein content and expressed as mean values (±SEM) over n $\geq$ 3 independent experiments per condition.

(H) *Il1b* expression relative to *Actb* of Mφ co-cultures with hiNSCs. Data are mean fold change versus $M\varphi^{LPS}$ (±SEM) from n $\geq$ 3 independent experiments per condition.

(I) XF assay showing the OCR of Mφ as in (H). Data are normalized on total protein content and expressed as mean values (±SEM) over n $\geq$ 2 independent experiments per condition.

(J) PGE2 secretion in Mφ co-cultures as in (H) and (I). Data are mean values (±SEM) over n $\geq$ 3 independent experiments per condition.

*p $\leq$ 0.05 versus untreated cells (B); *p $\leq$ 0.05 and **p $\leq$ 0.01 (C and D); *p $\leq$ 0.05, **p $\leq$ 0.01, and ***p $\leq$ 0.001 versus $M\varphi^{LPS}$ (E–J); $^{\#}$p $\leq$ 0.05 and $^{\#\#}$p $\leq$ 0.01 versus $M\varphi^{LPS}$-NSCs (E–G) or versus $M\varphi^{LPS}$-hiNSCs (H and I). See also Figure S4 and Table S4.

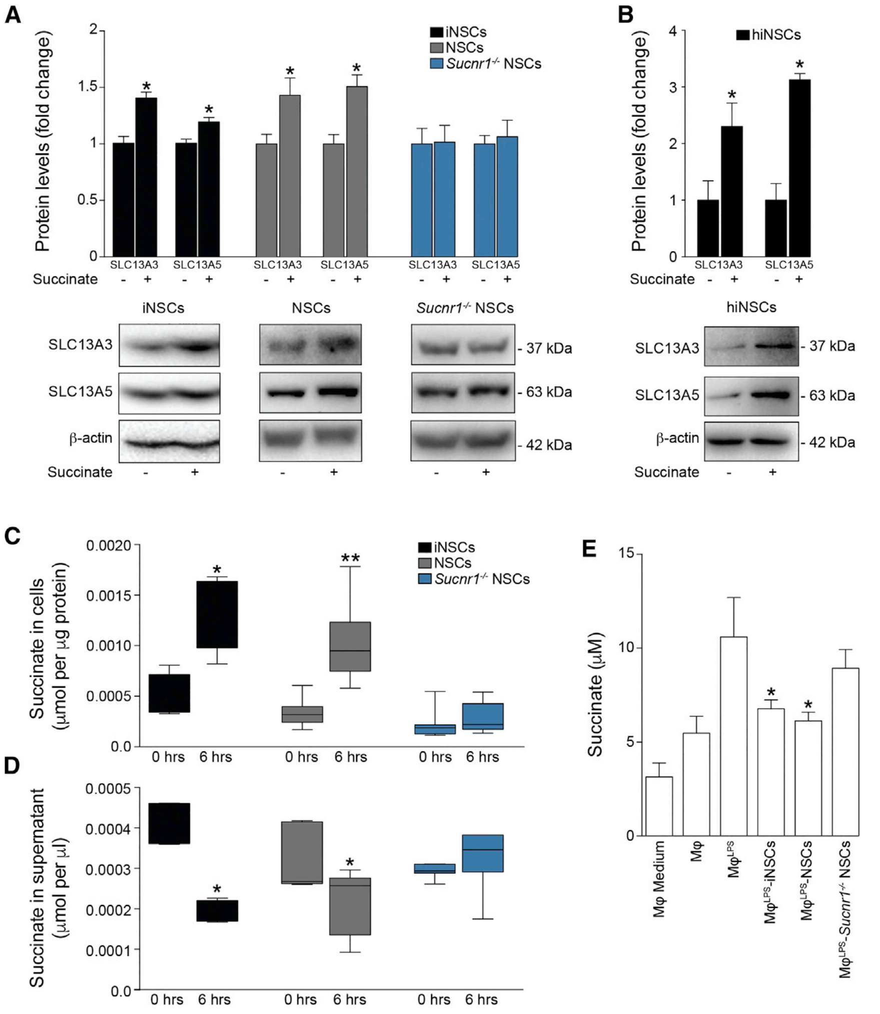

Figure 5. SUCNR1 Regulates the Uptake of Succinate by NSCs *In Vitro*

(A) SLC13A3 and SLC13A5 protein expression levels after 2 hr of succinate treatment.

(B) SLC13A3 and SLC13A5 protein expression levels after 6 hr of succinate treatment in hiNSCs. Data in (A) and (B) are relative to β-actin and expressed as mean fold change (±SEM) versus untreated cells over $n \geq 3$ independent experiments per condition.

(C and D) Uptake assay of [^{14}C]-labeled succinate at 0 and 6 hr. (C) Intracellular [^{14}C] labeling and (D) extracellular [^{14}C] signal in tissue culture media are shown. Box-whiskers plots ± min to max value from $n \geq 4$ technical replicates per group from n = 2 independent experiments are shown.

(E) Succinate release in Mφ co-cultures. Data are mean values versus Mφ (±SEM) from $n \geq 2$ independent experiments per condition.

*$p \leq 0.05$ versus untreated cells (A and B) or versus $M\varphi^{LPS}$ (E); *$p \leq 0.05$ and **$p \leq 0.01$ versus 0 hr, Mann-Whitney test (C and D). See also Figure S5.

SUCNR1 Stimulation Triggers the Uptake of Succinate by NSCs

Gene expression arrays of succinate-stimulated NSCs revealed that, besides *Ptgs2*, *NaCT/Slc13a5* was among the most upregulated genes in wild-type (WT) NSCs ($\log_2$ fold change = 0.49), but not in *Sucnr1*$^{-/-}$ NSCs ($\log_2$ fold change = −0.12). SLC13A5 is a dicarboxylate co-transporter known to be involved in succinate transport (Srisawang et al., 2007). Given the consistent depletion of succinate found both *in vivo* in the CSF of iNSC- or NSC-transplanted EAE mice and *in vitro* in co-cultures with $M\varphi^{LPS}$, we hypothesized that iNSCs/NSCs would activate SLC13A5 to scavenge succinate.

We found that the expression of SLC13A5, as well as of the high-affinity dicarboxylate co-transporter SLC13A3, were significantly increased in iNSCs and NSCs, but not in *Sucnr1*$^{-/-}$ NSCs, upon succinate stimulation (Figure 5A). Similarly, hiNSCs exposed to succinate upregulated the protein expression levels of both these SLC13 co-transporters *in vitro* (Figure 5B).

We next investigated the role of these co-transporters by measuring succinate uptake in iNSCs and NSCs. We found that both iNSCs and NSCs significantly accumulated [^{14}C]-succinate (Figure 5C) while reducing the amount of extracellular [^{14}C]-succinate in tissue culture media (Figure 5D). *Sucnr1*$^{-/-}$ NSCs neither accumulated [^{14}C]-succinate intracellularly nor did they deplete it extracellularly (Figures 5C and 5D). Interestingly, *Sucnr1*$^{-/-}$ NSCs, which we have shown to have no effects on *Il1b* expression in $M\varphi^{LPS}$ (Figure 4F), failed to reduce the extracellular succinate levels in co-cultures with $M\varphi^{LPS}$ (Figure 5E). As further proof of the importance of succinate depletion in modulating the phenotype of type 1 pro-inflammatory MPs, we show that treatment with active recombinant (r)SDH complex subunit A is able to significantly reduce the expression of *Il1b* in $M\varphi^{LPS}$ (Figure S5).

Thus, SUCNR1 signaling in NSCs prompts the uptake of the immunometabolite succinate, thereby depleting the available extracellular pool sustaining the autocrine and paracrine activation of type 1 MPs.

Transplantation of *Sucnr1* Loss-of-Function NSCs Shows Impaired Ability to Ameliorate Chronic Neuroinflammation *In Vivo*

To confirm the role of the succinate-SUCNR1 axis in mediating the response of NSC grafts to succinate *in vivo*, we assessed the effects of the icv transplantation of *Sucnr1*$^{-/-}$ NSCs in mice with chronic EAE.

At 30 dpt, *Sucnr1*$^{-/-}$ NSCs survived, distributed, and integrated within the EAE brain and spinal cord with no significant differences compared to control NSCs (Figure S6). However, the transplantation of *Sucnr1*$^{-/-}$ NSCs induced only a slight recovery of EAE behavioral deficits versus PBS-treated control EAE mice (EAE score—*Sucnr1*$^{-/-}$ NSCs: 2.9 ± 0.2; PBS: 3.6 ± 0.4), which was significantly less pronounced (50% of the effect) than that observed in EAE mice transplanted with control NSCs (EAE score—NSCs: 2.1 ± 0.3; Figure 6A).

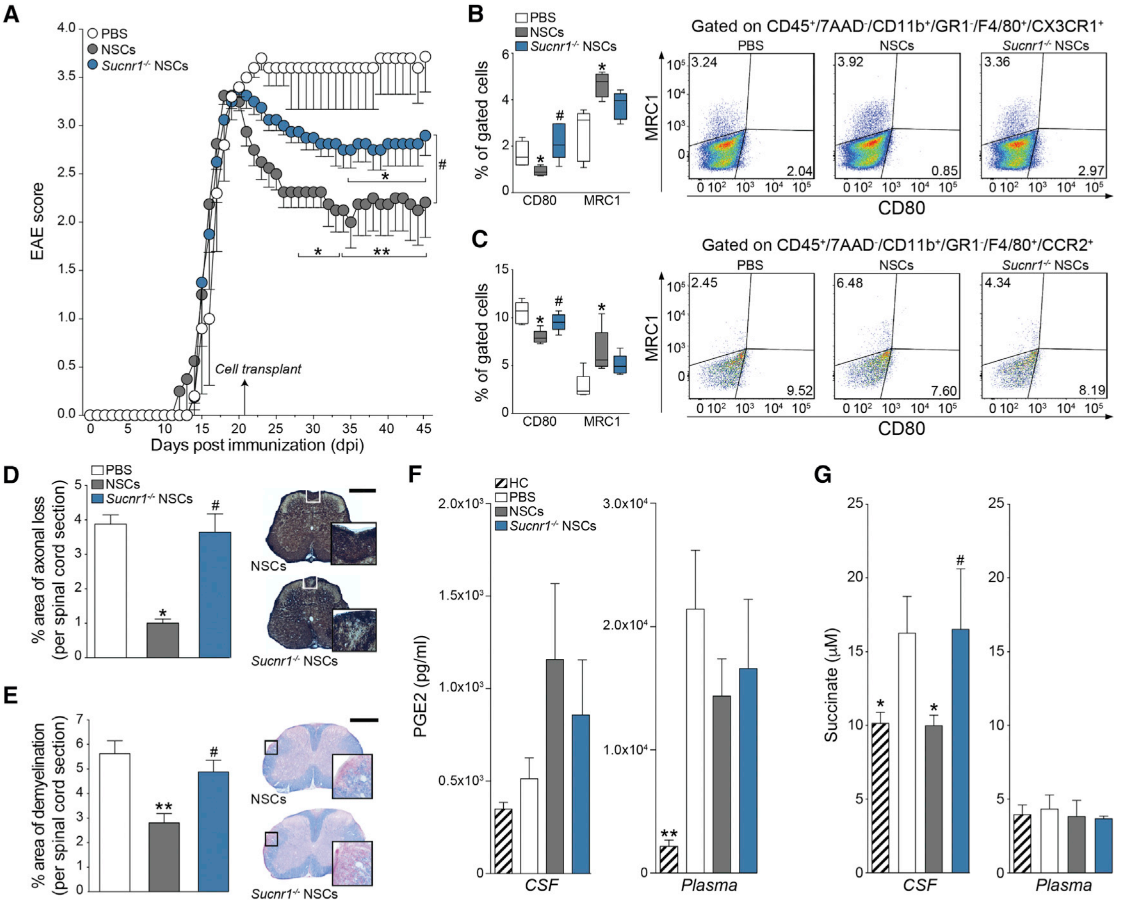

Figure 6. Transplantation of *Sucnr1* Loss-of-Function NSCs Shows Impaired Ability to Ameliorate Chronic Neuroinflammation *In Vivo*

(A) Behavioral outcome of EAE mice. Data are mean EAE score (±SEM) from n ≥ 5 mice/group.

(B and C) Flow-cytometry-based *ex vivo* quantification of the expression levels of type 1 inflammatory (CD80) and anti-inflammatory (MRC1) markers in CX3CR1$^+$ microglial cells (B) and CCR2$^+$ monocyte-derived infiltrating macrophages (C) at 30 dpt. Quantitative data are shown on the left, whereas representative density plots are shown on the right. Data are min to max % of marker-positive cells from n ≥ 4 pools of mice/group.

(D and E) Pathological outcomes of experiments as in (A). Data are mean % Bielschowsky negative-stained axonal loss (D) or LFB negative-stained demyelinated (E) areas/spinal cord section (±SEM) from n ≥ 4 mice/group. The scale bars represent 400 μm.

(F) PGE2 levels in the CSF and plasma of EAE mice at 30 dpt. Data are mean values (±SEM) from n ≥ 3 samples/group.

(G) Succinate levels in the CSF and plasma of EAE mice at 30 dpt. Data are mean values (±SEM) from n ≥ 4 mice/group. Kruskal-Wallis followed by Mann-Whitney post-test is shown.

*p ≤ 0.05, **p ≤ 0.01, and ***p ≤ 0.001 versus PBS; $^{\#}$p ≤ 0.05 versus NSCs. See also Figure S6.

Ex vivo flow-cytometry-based analysis of the composition of CNS inflammatory infiltrates showed that transplantation of EAE mice with *Sucnr1*$^{-/-}$ NSCs failed to shift the proportions of type 1 inflammatory and anti-inflammatory MPs—including CX3CR1$^+$ microglia and CCR2$^+$ monocyte-derived infiltrating macrophages—in contrast with the effects of control NSCs (Figures 6B and 6C). *Post mortem* tissue pathology further confirmed the reduced tissue-protective effects of *Sucnr1*$^{-/-}$ NSC grafts (Figures 6D and 6E).

We then investigated the levels of PGE2 and succinate in matched CSF and plasma samples from NSC-transplanted and PBS-treated control EAE mice. We found that both *Sucnr1*$^{-/-}$ NSCs and control NSCs failed to induce significant changes of the levels of PGE2 in the CSF. Plasma PGE2 significantly increased in EAE mice only (versus healthy controls), with no treatment effect observed (Figure 6F). Importantly, whereas transplantation of control NSCs reduced CSF succinate (HC: 5.524 × 10^7 a.u. ± 0.19; PBS: 9.35 × 10^7 a.u. ± 0.14; NSCs: 5.64 × 10^7 a.u. ± 0.44), *Sucnr1*$^{-/-}$ NSC grafts showed no effects (*Sucnr1*$^{-/-}$ NSCs: 10.40 × 10^7 a.u. ± 2.59; Figure 6G).

These data confirm the requirement of a functional SUCNR1 signaling pathway in the regulation of the anti-inflammatory and neuroprotective effects of NSC transplants *in vivo* and underline the importance of succinate scavenging as a predominant anti-inflammatory mechanism of action of NSCs.

DISCUSSION

There is an unmet clinical need to develop cellular and molecular approaches to target core drivers of the pathophysiology of chronic neuroinflammatory conditions that include progressive forms of MS (Volpe et al., 2016). In principle, stem cells possess

a therapeutic potential that is distinct from that of small molecules and biologics and extend far beyond the classical regenerative medicine arena. Part drug and part device, stem cells could work as biological disease-modifying agents (DMAs) that sense diverse signals, migrate to specific sites in the body, integrate inputs to make decisions, and execute complex response behaviors in the context of a specific tissue microenvironment (Fischbach et al., 2013). All these attributes could be harnessed to treat several disease processes, including the persistent MP-driven inflammation and tissue degeneration that occur in progressive MS.

Here, we used accessible, autologous, and stably expandable iNSCs (Thier et al., 2012), as well as somatic NSCs, to investigate the effects of brain stem cell transplantation in a mouse model of chronic neuroinflammation, which mimics the inflammatory cascade observed in progressive MS.

We found that the transplantation of iNSCs into the CSF circulation of EAE mice promotes equivalent outcomes to those previously observed in mice transplanted with somatic NSCs (Pluchino et al., 2003). Transplanted iNSCs or NSCs induced significant clinical amelioration, as well as reduced axonal and myelin damage, with no significant reduction of BBB permeability at 30 dpt. Further studies will help clarify whether changes of BBB permeability or recruitment of inflammatory monocytes to the CNS occur immediately following the transplantation of iNSCs/NSCs. Whether such an effect is likely to change the main clinical outcomes of diseases with high prevalence of CNS infiltration by inflammatory cells, such as EAE/MS, is hard to anticipate.

Instead, we found that our paradigm of transplantation was associated with a specific switch in the activation profile of both CX3CR1$^+$ microglial cells and CCR2$^+$ monocyte-derived infiltrating macrophages with a decrease of the CD80$^+$ type 1 inflammatory MPs and parallel increase of the MRC1$^+$ anti-inflammatory MPs. Transplanted iNSCs/NSCs distributed and survived in the CNS of EAE mice, while preferentially accumulating at the level of meningeal perivascular areas in juxtaposition to endogenous MPs. Altogether, these findings would imply the presence of some yet unknown mechanisms of intercellular coupling between grafted stem cells and inflammatory MPs. Whether this iNSC/NSC-MP communication *in vivo* takes place only in perivascular niches or also at the level of other emerging immune sensor-like structures of the brain that include the choroid plexus remains to be addressed (Ge et al., 2017).

We then investigated the underlying immunological mechanisms driving the beneficial effects of NSCs on MPs during chronic neuroinflammation. Untargeted small molecule analysis of matched CSF and plasma samples revealed profound metabolic changes in the CSF of EAE mice, with differences between the early and the delayed phases of disease.

Carnitine, leucine + isoleucine, citrulline, allantoin, ornithine, and uric acid were all significantly increased in the PBS-treated control EAE mice at the peak of disease. Our findings are consistent with published evidence showing that leucine, as well as uric acid and its by-product allantoin, are all increased in the CSF of subjects with MS (Amorini et al., 2009; Hooper et al., 1998; Monaco et al., 1979). Whereas increased CSF carnitine has not been reported in MS, important increases have been described in non-MS inflammatory conditions of the CNS, such as encephalitis (Wikoff et al., 2008) and meningitis (Shinawi et al., 1998).

Conversely, only succinate showed a delayed (i.e., 45 dpi) increase in the CSF of PBS-treated control EAE mice. Succinate is becoming a valuable *in vivo* biomarker of metabolic distress and inflammatory activity (Littlewood-Evans et al., 2016; Mills and O'Neill, 2014). Importantly, we found that succinate was significantly decreased in the CSF of iNSC-/NSC-transplanted mice. The reduction of CSF succinate following iNSC or NSC transplantation was of interest and might have a prominent role in interfering with chronic neuroinflammation.

Succinate released from type 1 inflammatory MPs is a key inflammatory signal that interacts with its specific G-protein-coupled receptor SUCNR1. SUCNR1 acts as an early detector of metabolic stress in several physiological and pathological conditions, including renin-induced hypertension, ischemia/reperfusion injury, inflammation, platelet aggregation, and retinal angiogenesis (de Castro Fonseca et al., 2016; Gilissen et al., 2016). Notably, we found that the expression of SUCNR1 is required for the therapeutic effects of transplanted NSCs *in vivo*.

Succinate-mediated activation of SUCNR1 on rodent and human iNSCs and NSCs activates calcium signaling and mitogen-activated protein kinase (MAPK) phosphorylation *in vitro*, thus eliciting the acquisition of a concerted anti-inflammatory phenotype in NSCs. On the one hand, SUCNR1 activated the secretion of PGE2, a well-established pleiotropic immune modulator, whose function targets multiple cell types within the inflamed microenvironment, including MPs (Kota et al., 2017; Vasandan et al., 2016). On the other hand, succinate-SUCNR1 signaling in iNSCs and NSCs led to the upregulation of two members of the SLC13 family of co-transporters (i.e., SLC13A3 and SLC13A5) and uptake of extracellular succinate.

In vivo, we demonstrate effective scavenging of extracellular local succinate by NSCs injected in EAE mice through the CSF circulation, which is predominant, compared to the secretion of PGE2. The loss of SUCNR1-dependent signaling in transplanted NSCs led to significant reduction in their anti-inflammatory and neuroprotective effects, whereas *Sucnr1*$^{-/-}$ NSC grafts showed no difference of survival, distribution, and differentiation versus control NSCs.

We then hypothesize that the extracellular succinate secreted by type 1 inflammatory MPs initiates a scavenging behavior that transplanted NSCs adjust in response to increased substrate availability (Srisawang et al., 2007). This novel intercellular metabolic coupling fits well with the available literature showing that, within specific microenvironments, cells compete for available nutrients, affecting each other's function and fate (Pearce and Pearce, 2013).

We anticipate that succinate depletion by SUCNR1-expressing iNSCs and NSCs might play a crucial role in reducing the availability of a key metabolic signal in inflammatory contexts where the interactions between transplanted stem cells and host immune cells become complementary (Pluchino and Cossetti, 2013). More generally, our findings are in line with the provocative, yet still emerging, concept of NSCs as ancestral guardians of the brain capable of exerting several complementary immune modulatory and tissue trophic effects (Martino and Pluchino, 2007).

Additional studies are needed to further characterize the function of the succinate-SUCNR1 axis in neuro-immune interactions, provide additional insights on the critical role of cellular

metabolism for neural stem/progenitor cells (Knobloch and Jessberger, 2017), and develop complementary pharmacological interventions targeting this pathway in the chronically inflamed brain.

In conclusion, we show here that NSCs sense the extracellular succinate that accumulates in the chronically inflamed CNS to ameliorate neuroinflammation via succinate-SUCNR1-dependent mechanisms. Our work identifies a novel anti-inflammatory mechanism that underpins the regenerative potential of somatic and directly induced NSCs, thus paving the way for a new era of personalized stem cell medicines for chronic inflammatory and degenerative neurological diseases.

STAR★METHODS

Detailed methods are provided in the online version of this paper and include the following:

- KEY RESOURCES TABLE
- CONTACT FOR REAGENT AND RESOURCE SHARING
- EXPERIMENTAL MODEL AND SUBJECT DETAILS
- METHOD DETAILS
  - Mouse iNSC/NSC proliferation, viability and differentiation *in vitro*
  - Fluorescence-activated cell sorting (FACS) analysis
  - Type-1 inflammatory Mφ or BV2 cells co-cultures with iNSCs/NSCs
  - Recombinant SDHA activity and treatment of type-1 inflammatory Mφ
  - Lentiviral fGFP tagging
  - EAE induction, transplantation and behavioral studies
  - *Ex vivo* tissue pathology
  - Blood brain barrier functional analysis
  - Plasma and cerebrospinal fluid (CSF) sampling
  - Calcium imaging
  - ELISA
  - Uptake experiments with [^{14}C] - labeled succinate
  - Immunoblotting
  - Extracellular flux (XF) assays
  - Gene expression analysis (microarrays and qRT-PCR)
  - Metabolite extraction and LC-MS analysis
- QUANTITATION AND STATISTICAL ANALYSES
- DATA AND SOFTWARE AVAILABILITY

SUPPLEMENTAL INFORMATION

Supplemental Information includes six figures and four tables and can be found with this article online at https://doi.org/10.1016/j.stem.2018.01.020.

ACKNOWLEDGMENTS

The authors thank P. Chinnery, F. Dazzi, D. Franciotta, J. Jones, A. Tolkovsky, and L. Vallier for critically discussing the article. The authors wish to acknowledge G. Pluchino, J. Smith, G. Tannahil, and K. Saeb-Parsy for their technical contributions and critical insights throughout the execution of the study and A. Elkalhoun and W. Wu of the NIH/NHGRI IRP Microarray Core Facility for their assistance with the transcriptomic/microarray studies. The authors also thank J.M. Carballido (Novartis, Switzerland) for providing the C57BL/6 *Sucnr1*$^{-/-}$ mice, A.L. Vescovi (Milano, Italy) for providing the RNA and protein extracts from human fetal NSCs (hNSCs), and S. De (Advinus Therapeutics TATA Enterprise, India) for providing the compound *4c* used in this study. This work was funded by the Italian Multiple Sclerosis Association (AISM) (grant 2010/R/31 and grant 2014/PMS/4 to S.P.), the United States Department of Defense (DoD) Congressionally Directed Medical Research Programs (CDMRP) (grant MS140019 to S.P.), the Italian Ministry of Health (GR08-7 to S.P.), the European Research Council (ERC) under the ERC-2010-StG grant agreement no. 260511-SEM_SEM, the Medical Research Council, the Engineering and Physical Sciences Research Council, the Biotechnology and Biological Sciences Research Council UK Regenerative Medicine Platform Hub "Acellular Approaches for Therapeutic Delivery" (MR/K026682/1 to S.P.), the Evelyn Trust (RG 69865 to S.P.) and the Bascule Charitable Trust (RG 75149 to S.P.). L.P.-J. was supported by a research training fellowship from the Wellcome Trust (RRZA/057 RG79423). J.D.B. was supported by a NIH-OxCam training fellowship. Work in the F.E. lab was supported by grants from the ERA-Net E-rare research programme and the Austrian Science Fund (FWF). C.K.K. was supported by a stipend of the German Excellence Initiative to the Graduate School of Life Sciences, University of Würzburg. J.M.H. was supported by the Intramural Research Program (IRP) of NINDS/NIH. Work in M.P.M.'s lab was supported by grants from the Medical Research Council UK (MC_U105663142) and by a Wellcome Trust Investigator award (110159/Z/15/Z).

AUTHOR CONTRIBUTIONS

Conceptualization, L.P.J., C.F., and S.P.; Methodology, L.P.J., F.E., and S.P.; Formal Analysis, L.P.J., J.D.B., N.V., A.S.H.C., T.L., C.F., and S.P.; Investigation, L.P.J., J.D.B., N.V., I.B., A.S.H.C., C.K.K., B.B., G. Mallucci, G. Manferrari., G.V., M.D., N.I., A.B., and L.M.B.; Resources, M.P.M., J.H., F.E., C.F., and S.P.; Data Curation, L.P.J., J.D.B., and N.V.; Writing – Original Draft, L.P.J., J.D.B., N.V., C.F., and S.P.; Writing – Review & Editing, L.P.J., J.D.B., N.V., M.P.M., J.H., F.E., C.F., and S.P.; Supervision, M.P.M., J.H., F.E., C.F., and S.P.; Project Administration, L.P.J., J.D.B., N.V., C.F., and S.P.; Funding Acquisition, L.P.J. and S.P.

DECLARATION OF INTERESTS

S.P. owns >5% of CITC.

Received: April 14, 2017
Revised: September 18, 2017
Accepted: January 25, 2018
Published: February 22, 2018

REFERENCES

Al-Shabany, A.J., Moody, A.J., Foey, A.D., and Billington, R.A. (2016). Intracellular NAD+ levels are associated with LPS-induced TNF-α release in pro-inflammatory macrophages. Biosci. Rep. *36*, e00301.

Amorini, A.M., Petzold, A., Tavazzi, B., Eikelenboom, J., Keir, G., Belli, A., Giovannoni, G., Di Pietro, V., Polman, C., D'Urso, S., et al. (2009). Increase of uric acid and purine compounds in biological fluids of multiple sclerosis patients. Clin. Biochem. *42*, 1001–1006.

Anderson, A.J., Piltti, K.M., Hooshmand, M.J., Nishi, R.A., and Cummings, B.J. (2017). Preclinical efficacy failure of human CNS-derived stem cells for use in the pathway study of cervical spinal cord injury. Stem Cell Reports *8*, 249–263.

Bacigaluppi, M., Pluchino, S., Peruzzotti-Jametti, L., Kilic, E., Kilic, U., Salani, G., Brambilla, E., West, M.J., Comi, G., Martino, G., and Hermann, D.M. (2009). Delayed post-ischaemic neuroprotection following systemic neural stem cell transplantation involves multiple mechanisms. Brain *132*, 2239–2251.

Bacigaluppi, M., Russo, G.L., Peruzzotti-Jametti, L., Rossi, S., Sandrone, S., Butti, E., De Ceglia, R., Bergamaschi, A., Motta, C., Gallizioli, M., et al. (2016). Neural stem cell transplantation induces stroke recovery by upregulating glutamate transporter GLT-1 in astrocytes. J. Neurosci. *36*, 10529–10544.

Bramow, S., Frischer, J.M., Lassmann, H., Koch-Henriksen, N., Lucchinetti, C.F., Sørensen, P.S., and Laursen, H. (2010). Demyelination versus remyelination in progressive multiple sclerosis. Brain *133*, 2983–2998.

Carvalho, B.S., and Irizarry, R.A. (2010). A framework for oligonucleotide microarray preprocessing. Bioinformatics *26*, 2363–2367.

Cusimano, M., Biziato, D., Brambilla, E., Donegà, M., Alfaro-Cervello, C., Snider, S., Salani, G., Pucci, F., Comi, G., Garcia-Verdugo, J.M., et al. (2012). Transplanted neural stem/precursor cells instruct phagocytes and reduce secondary tissue damage in the injured spinal cord. Brain *135*, 447–460.

de Castro Fonseca, M., Aguiar, C.J., da Rocha Franco, J.A., Gingold, R.N., and Leite, M.F. (2016). GPR91: expanding the frontiers of Krebs cycle intermediates. Cell Commun. Signal. *14*, 3.

Eftaxiopoulou, T., Macdonald, W., Britzman, D., and Bull, A.M.J. (2014). Gait compensations in rats after a temporary nerve palsy quantified using temporo-spatial and kinematic parameters. Journal of Neuroscience Methods *232*, 16–23.

Fischbach, M.A., Bluestone, J.A., and Lim, W.A. (2013). Cell-based therapeutics: the next pillar of medicine. Sci. Transl. Med. *5*, 179ps7.

Ge, R., Tornero, D., Hirota, M., Monni, E., Laterza, C., Lindvall, O., and Kokaia, Z. (2017). Choroid plexus-cerebrospinal fluid route for monocyte-derived macrophages after stroke. J. Neuroinflammation *14*, 153.

Gilissen, J., Jouret, F., Pirotte, B., and Hanson, J. (2016). Insight into SUCNR1 (GPR91) structure and function. Pharmacol. Ther. *159*, 56–65.

Grupp, C., and Müller, G.A. (1999). Renal fibroblast culture. Exp. Nephrol. *7*, 377–385.

Hooper, D.C., Spitsin, S., Kean, R.B., Champion, J.M., Dickson, G.M., Chaudhry, I., and Koprowski, H. (1998). Uric acid, a natural scavenger of peroxynitrite, in experimental allergic encephalomyelitis and multiple sclerosis. Proc. Natl. Acad. Sci. USA *95*, 675–680.

Jiang, Z., Jiang, J.X., and Zhang, G.X. (2014). Macrophages: a double-edged sword in experimental autoimmune encephalomyelitis. Immunol. Lett. *160*, 17–22.

Kelly, B., and O'Neill, L.A. (2015). Metabolic reprogramming in macrophages and dendritic cells in innate immunity. Cell Res. *25*, 771–784.

Knobloch, M., and Jessberger, S. (2017). Metabolism and neurogenesis. Curr. Opin. Neurobiol. *42*, 45–52.

Kota, D.J., Prabhakara, K.S., Toledano-Furman, N., Bhattarai, D., Chen, Q., DiCarlo, B., Smith, P., Triolo, F., Wenzel, P.L., Cox, C.S., Jr., et al. (2017). Prostaglandin E2 indicates therapeutic efficacy of mesenchymal stem cells in experimental traumatic brain injury. Stem Cells *35*, 1416–1430.

Littlewood-Evans, A., Sarret, S., Apfel, V., Loesle, P., Dawson, J., Zhang, J., Muller, A., Tigani, B., Kneuer, R., Patel, S., et al. (2016). GPR91 senses extracellular succinate released from inflammatory macrophages and exacerbates rheumatoid arthritis. J. Exp. Med. *213*, 1655–1662.

Liu, L., and Duff, K. (2008). A technique for serial collection of cerebrospinal fluid from the cisterna magna in mouse. J. Vis. Exp. (21), 960.

Lu, J., Liu, H., Huang, C.T., Chen, H., Du, Z., Liu, Y., Sherafat, M.A., and Zhang, S.C. (2013). Generation of integration-free and region-specific neural progenitors from primate fibroblasts. Cell Rep. *3*, 1580–1591.

Mallucci, G., Peruzzotti-Jametti, L., Bernstock, J.D., and Pluchino, S. (2015). The role of immune cells, glia and neurons in white and gray matter pathology in multiple sclerosis. Prog. Neurobiol. *127-128*, 1–22.

Martinez, P., Denys, A., Delos, M., Sikora, A.S., Carpentier, M., Julien, S., Pestel, J., and Allain, F. (2015). Macrophage polarization alters the expression and sulfation pattern of glycosaminoglycans. Glycobiology *25*, 502–513.

Martino, G., and Pluchino, S. (2006). The therapeutic potential of neural stem cells. Nat. Rev. Neurosci. *7*, 395–406.

Martino, G., and Pluchino, S. (2007). Neural stem cells: guardians of the brain. Nat. Cell Biol. *9*, 1031–1034.

Masters, S.L., Dunne, A., Subramanian, S.L., Hull, R.L., Tannahill, G.M., Sharp, F.A., Becker, C., Franchi, L., Yoshihara, E., Chen, Z., et al. (2010). Activation of the NLRP3 inflammasome by islet amyloid polypeptide provides a mechanism for enhanced IL-1β in type 2 diabetes. Nat. Immunol. *11*, 897–904.

Mazzini, L., Gelati, M., Profico, D.C., Sgaravizzi, G., Projetti Pensi, M., Muzi, G., Ricciolini, C., Rota Nodari, L., Carletti, S., Giorgi, C., et al. (2015). Human neural stem cell transplantation in ALS: initial results from a phase I trial. J. Transl. Med. *13*, 17.

Meyer, S., Wörsdörfer, P., Günther, K., Thier, M., and Edenhofer, F. (2015). Derivation of adult human fibroblasts and their direct conversion into expandable neural progenitor cells. J. Vis. Exp. *101*, e52831.

Mills, E., and O'Neill, L.A. (2014). Succinate: a metabolic signal in inflammation. Trends Cell Biol. *24*, 313–320.

Mills, E.L., Kelly, B., Logan, A., Costa, A.S.H., Varma, M., Bryant, C.E., Tourlomousis, P., Dabritz, J.H.M., Gottlieb, E., Latorre, I., et al. (2016). Succinate dehydrogenase supports metabolic repurposing of mitochondria to drive inflammatory macrophages. Cell *167*, 457–470.e13.

Moll, N.M., Rietsch, A.M., Thomas, S., Ransohoff, A.J., Lee, J.C., Fox, R., Chang, A., Ransohoff, R.M., and Fisher, E. (2011). Multiple sclerosis normal-appearing white matter: pathology-imaging correlations. Ann. Neurol. *70*, 764–773.

Monaco, F., Fumero, S., Mondino, A., and Mutani, R. (1979). Plasma and cerebrospinal fluid tryptophan in multiple sclerosis and degenerative diseases. J. Neurol. Neurosurg. Psychiatry *42*, 640–641.

O'Neill, L.A., and Pearce, E.J. (2016). Immunometabolism governs dendritic cell and macrophage function. J. Exp. Med. *213*, 15–23.

Orihuela, R., McPherson, C.A., and Harry, G.J. (2016). Microglial M1/M2 polarization and metabolic states. Br. J. Pharmacol. *173*, 649–665.

Palsson-McDermott, E.M., Curtis, A.M., Goel, G., Lauterbach, M.A., Sheedy, F.J., Gleeson, L.E., van den Bosch, M.W., Quinn, S.R., Domingo-Fernandez, R., Johnston, D.G., et al. (2015). Pyruvate kinase M2 regulates Hif-1α activity and IL-1β induction and is a critical determinant of the warburg effect in LPS-activated macrophages. Cell Metab. *21*, 65–80.

Pearce, E.L., and Pearce, E.J. (2013). Metabolic pathways in immune cell activation and quiescence. Immunity *38*, 633–643.

Peterson, J.W., Bö, L., Mörk, S., Chang, A., and Trapp, B.D. (2001). Transected neurites, apoptotic neurons, and reduced inflammation in cortical multiple sclerosis lesions. Ann. Neurol. *50*, 389–400.

Planche, V., Panatier, A., Hiba, B., Ducourneau, E.G., Raffard, G., Dubourdieu, N., Maitre, M., Lesté-Lasserre, T., Brochet, B., Dousset, V., et al. (2017). Selective dentate gyrus disruption causes memory impairment at the early stage of experimental multiple sclerosis. Brain Behav. Immun. *60*, 240–254.

Pluchino, S., and Cossetti, C. (2013). How stem cells speak with host immune cells in inflammatory brain diseases. Glia *61*, 1379–1401.

Pluchino, S., Quattrini, A., Brambilla, E., Gritti, A., Salani, G., Dina, G., Galli, R., Del Carro, U., Amadio, S., Bergami, A., et al. (2003). Injection of adult neurospheres induces recovery in a chronic model of multiple sclerosis. Nature *422*, 688–694.

Pluchino, S., Zanotti, L., Rossi, B., Brambilla, E., Ottoboni, L., Salani, G., Martinello, M., Cattalini, A., Bergami, A., Furlan, R., et al. (2005). Neurosphere-derived multipotent precursors promote neuroprotection by an immunomodulatory mechanism. Nature *436*, 266–271.

Pluchino, S., Muzio, L., Imitola, J., Deleidi, M., Alfaro-Cervello, C., Salani, G., Porcheri, C., Brambilla, E., Cavasinni, F., Bergamaschi, A., et al. (2008). Persistent inflammation alters the function of the endogenous brain stem cell compartment. Brain *131*, 2564–2578.

Pluchino, S., Gritti, A., Blezer, E., Amadio, S., Brambilla, E., Borsellino, G., Cossetti, C., Del Carro, U., Comi, G., 't Hart, B., et al. (2009a). Human neural stem cells ameliorate autoimmune encephalomyelitis in non-human primates. Ann. Neurol. *66*, 343–354.

Pluchino, S., Zanotti, L., Brambilla, E., Rovere-Querini, P., Capobianco, A., Alfaro-Cervello, C., Salani, G., Cossetti, C., Borsellino, G., Battistini, L., et al. (2009b). Immune regulatory neural stem/precursor cells protect from central nervous system autoimmunity by restraining dendritic cell function. PLoS ONE *4*, e5959.

Prineas, J.W., Kwon, E.E., Cho, E.S., Sharer, L.R., Barnett, M.H., Oleszak, E.L., Hoffman, B., and Morgan, B.P. (2001). Immunopathology of secondary-progressive multiple sclerosis. Ann. Neurol. *50*, 646–657.

Ramos-Zúñiga, R., González-Pérez, O., Macías-Ornelas, A., Capilla-González, V., and Quiñones-Hinojosa, A. (2012). Ethical implications in the use of embryonic and adult neural stem cells. Stem Cells Int. *2012*, 470949.

Rice, C.M., Kemp, K., Wilkins, A., and Scolding, N.J. (2013). Cell therapy for multiple sclerosis: an evolving concept with implications for other neurodegenerative diseases. Lancet *382*, 1204–1213.

Ritchie, M.E., Phipson, B., Wu, D., Hu, Y., Law, C.W., Shi, W., and Smyth, G.K. (2015). limma powers differential expression analyses for RNA-sequencing and microarray studies. Nucleic Acids Res. *43*, e47.

Rubic, T., Lametschwandtner, G., Jost, S., Hinteregger, S., Kund, J., Carballido-Perrig, N., Schwärzler, C., Junt, T., Voshol, H., Meingassner, J.G., et al. (2008). Triggering the succinate receptor GPR91 on dendritic cells enhances immunity. Nat. Immunol. *9*, 1261–1269.

Ryu, J.K., Nagai, A., Kim, J., Lee, M.C., McLarnon, J.G., and Kim, S.U. (2003). Microglial activation and cell death induced by the mitochondrial toxin 3-nitropropionic acid: in vitro and in vivo studies. Neurobiol. Dis. *12*, 121–132.

Shinawi, M., Gruener, N., and Lerner, A. (1998). CSF levels of carnitine in children with meningitis, neurologic disorders, acute gastroenteritis, and seizure. Neurology *50*, 1869–1871.

Srisawang, P., Chatsudthipong, A., and Chatsudthipong, V. (2007). Modulation of succinate transport in Hep G2 cell line by PKC. Biochim. Biophys. Acta *1768*, 1378–1388.

Tambalo, S., Peruzzotti-Jametti, L., Rigolio, R., Fiorini, S., Bontempi, P., Mallucci, G., Balzarotti, B., Marmiroli, P., Sbarbati, A., Cavaletti, G., et al. (2015). Functional magnetic resonance imaging of rats with experimental autoimmune encephalomyelitis reveals brain cortex remodeling. J. Neurosci. *35*, 10088–10100.

Tannahill, G.M., Curtis, A.M., Adamik, J., Palsson-McDermott, E.M., McGettrick, A.F., Goel, G., Frezza, C., Bernard, N.J., Kelly, B., Foley, N.H., et al. (2013). Succinate is an inflammatory signal that induces IL-1β through HIF-1α. Nature *496*, 238–242.

Tannahill, G.M., Iraci, N., Gaude, E., Frezza, C., and Pluchino, S. (2015). Metabolic reprograming of mononuclear phagocytes in progressive multiple sclerosis. Front. Immunol. *6*, 106.

Thier, M., Wörsdörfer, P., Lakes, Y.B., Gorris, R., Herms, S., Opitz, T., Seiferling, D., Quandel, T., Hoffmann, P., Nöthen, M.M., et al. (2012). Direct conversion of fibroblasts into stably expandable neural stem cells. Cell Stem Cell *10*, 473–479.

Vasandan, A.B., Jahnavi, S., Shashank, C., Prasad, P., Kumar, A., and Prasanna, S.J. (2016). Human Mesenchymal stem cells program macrophage plasticity by altering their metabolic status via a PGE2-dependent mechanism. Sci. Rep. *6*, 38308.

Vescovi, A.L., and Snyder, E.Y. (1999). Establishment and properties of neural stem cell clones: plasticity in vitro and in vivo. Brain Pathol. *9*, 569–598.

Volpe, G., Bernstock, J.D., Peruzzotti-Jametti, L., and Pluchino, S. (2016). Modulation of host immune responses following non-hematopoietic stem cell transplantation: Translational implications in progressive multiple sclerosis. J. Neuroimmunol., S0165-5728(16)30312-5.

Wang, H.L., and Lai, T.W. (2014). Optimization of Evans blue quantitation in limited rat tissue samples. Sci. Rep. *4*, 6588.

Wikoff, W.R., Pendyala, G., Siuzdak, G., and Fox, H.S. (2008). Metabolomic analysis of the cerebrospinal fluid reveals changes in phospholipase expression in the CNS of SIV-infected macaques. J. Clin. Invest. *118*, 2661–2669.

Wörsdörfer, P., Thier, M., Kadari, A., and Edenhofer, F. (2013). Roadmap to cellular reprogramming–manipulating transcriptional networks with DNA, RNA, proteins and small molecules. Curr. Mol. Med. *13*, 868–878.

Wright, L.S., Prowse, K.R., Wallace, K., Linskens, M.H., and Svendsen, C.N. (2006). Human progenitor cells isolated from the developing cortex undergo decreased neurogenesis and eventual senescence following expansion in vitro. Exp. Cell Res. *312*, 2107–2120.

Yañez, R., Oviedo, A., Aldea, M., Bueren, J.A., and Lamana, M.L. (2010). Prostaglandin E2 plays a key role in the immunosuppressive properties of adipose and bone marrow tissue-derived mesenchymal stromal cells. Exp. Cell Res. *316*, 3109–3123.

STAR★METHODS

KEY RESOURCES TABLE

REAGENT or RESOURCE	SOURCE	IDENTIFIER
Antibodies		
AffiniPure Fab Fragment Goat Anti-Mouse IgG (H+L)	Jackson	115-007-003
Alexa Fluor 488 Rat anti-Mouse Foxp3	BD Biosciences	756348
Alexa Fluor 594 anti-mouse Ly-6G/Ly-6C (Gr-1)	BioLegend	108448
Alexa Fluor 647 Rat Anti-Mouse CD4 Clone RM4-5 (RUO)	BD Biosciences	557681
Alexa Fluor 700 Rat Anti-Mouse IFN-γ	BD Biosciences	557998
Alexa Fluor 647 anti-mouse CCR2	Biolegend	150603
APC/Cy7 anti-mouse F4/80	Biolegend	123117
Brilliant Violet 421 Goat anti-rat IgG	BioLegend	405414
Brilliant Violet 711 anti-mouse CD206	BioLegend	141727
BUV395 Hamster Anti-Mouse CD3e Clone 500A2 (RUO)	BD Biosciences	740221
BV421 Rat Anti-Mouse GM-CSF	BD Biosciences	564747
BV605 Hamster Anti-Mouse CD80	BD Biosciences	563052
BV786 Rat Anti-Mouse IL-4	BD Biosciences	564006
CD45 MicroBeads	Milteny	130-052-301
Chicken anti-GFP (polyclonal)	abcam	ab13870
Chicken anti-Nestin (polyclonal)	abcam	ab134017
Donkey anti-goat AF488 conjugated secondary	abcam	AB150129
Donkey anti-goat Biotin conjugated secondary	adb serotech	642008
FITC Rat Anti-CD11b M1/70	BD Biosciences	553310
Goat anti mouse-HRP conjugated secondary	Thermo Scientific	31430
Goat anti-CD20 (polyclonal)	Santa Cruz	sc-7735
Goat anti-chicken AF488 conjugated secondary	Invitrogen	A11039
Goat anti-chicken AF555 conjugated secondary	Invitrogen	A21237
Goat anti-chicken AF647 conjugated secondary	Lifetech	A21449
Goat anti-chicken IgG biotin conjugated secondary	vector laboratories	BA-9010
Goat anti-il1β (polyclonal)	R&D systems	AF-401-NA
Goat anti-mouse AF488 conjugated secondary	Invitrogen	A11001
Goat anti-mouse AF546 conjugated secondary	Invitrogen	A21045
Goat anti-mouse AF647 conjugated secondary	Invitrogen	A21235
Goat anti-rabbit AF488 conjugated secondary	Invitrogen	A11008
Goat anti-rabbit AF546 conjugated secondary	Invitrogen	A11010
Goat anti-rabbit AF647 conjugated secondary	Invitrogen	A21244
Goat anti-rabbit HRP conjugated secondary	Thermo Scientific	31460
Goat anti-Rat AF405 conjugated secondary	abcam	ab175671
Goat anti-Rat AF488 conjugated secondary	Invitrogen	A11006
Goat anti-Rat AF546 conjugated secondary	Invitrogen	A11081
Mouse anti-CD3 (clone: PS1)	abcam	ab699
Mouse anti-GFAP (clone: 52/GFAP)	BD Biosciences	610566
Mouse anti-iNOS	BD Biosciences	610329
Mouse anti-NeuN (clone: A60)	Millipore	MAB377
Mouse anti-O4 (clone: O4)	R&D systems	MAB1326
Mouse anti-β-actin (clone: AC-15)	Sigma Aldrich	A1978
Mouse anti-β-tubulin (clone: TUB 2.1)	Sigma Aldrich	T4026

(*Continued on next page*)

Continued

REAGENT or RESOURCE	SOURCE	IDENTIFIER
PE anti-mouse CX3CR1	BioLegend	149005
PE Mouse anti-Mouse RORγt	BD Biosciences	562607
PE-Cy7 Rat Anti-Mouse CD19	BD Biosciences	552854
PerCP-Cy5.5 Rat Anti-Mouse IL-17A	BD Biosciences	560666
Purified anti-P2RY12 Antibody	BioLegend	848002
Rabbit anti-MRC1 (polyclonal)	abcam	ab64693
Rabbit anti-GFAP (polyclonal)	DAKO	Z0334
Rabbit anti-iNOS (polyclonal)	abcam	ab3523
Rabbit anti-Ki67 (polyclonal)	abcam	ab15580
Rabbit anti-MRC1 (polyclonal)	abcam	Ab64693
Rabbit anti-Olig2 (polyclonal)	Chemicon	AB9610
Rabbit anti-p38 MAPK (polyclonal)	Cell Signaling	9212
Rabbit anti-Phospho-p38 MAPK (polyclonal)	Cell Signaling	9211
Rabbit anti-Pkm2 (polyclonal)	Cell Signaling	3198
Rabbit anti-SLC13A3 (polyclonal)	Aviva Systems biology	ARP41438_T100
Rabbit anti-SLC13A5 (polyclonal)	ThermoFisher	PA5-24675
Rabbit anti-SOX2 (polyclonal)	Abcam	ab15830
Rabbit anti-Sucnr1 (polyclonal)	Novus Biologicals	NBP1-00861
Rabbit anti-vWF (polyclonal)	Abcam	ab6994
Rabbit HIF1α (polyclonal)	Novus Biologicals	NB100-134
Rat anti-CD45 (clone: 30-F11)	BD Biosciences	550539
Rat anti-F4/80 (clone: Cl:A3-1)	Bio-Rad	MCA497R
Rat anti-MBP (aa82-87) (clone: 12)	Bio-Rad	MCA409S
Rat anti-mouse FcγIII/II receptor (CD16/CD32)	BD Biosciences	558636
**Chemicals, Peptides, and Recombinant Proteins**		
[^{14}C]-succinic acid	American Radiolabelled Chemicals	ARC 3593-50μCi
4'6-diamidino-2-phenylindole (DAPI)	Invitrogen	D1306
4C	Advinus Therapeutics	N/A
7-amino-actinomycin D	BioLegend	420403
Accumax	Affymetrix	00-4666
Adenosine 5′-triphosphate disodium salt hydrate (ATP)	Sigma	A7699
AutoMACS Rinsing Solution	Milteny	130-091-222
B27 w/vitamin A (50x)	GIBCO	17504-044
Basal Fibroblast Growth Factor	Peprotech	100-18B-1000
Basement Membrane Matrix Growth Factor Reduced	Corning	354230
CHIR 99021	Axon MedChem	1386
Collagenase IV	Sigma	C9891
Dialyzed Foetal Bovine Serum (dFBS)	GIBCO	26400036
Dimethyl malonate	Sigma Aldrich	136441
Dispase 25U	MP Bio	195022
DMEM high glucose	GIBCO	41966029
DMEM/F12	GIBCO	11320-033
DNase I 5000U	Biolabs	M0303L
DNase Buffer	Biolabs	B0303S
Evans Blue	Sigma	E2129-50G
Flow Cytometry Staining Buffer	BioLegend	420201
Fluo-4AM	Life Technologies	F-14217
Foetal bovine serum (FBS)	GIBCO	10500-064

(*Continued on next page*)

Continued

REAGENT or RESOURCE	SOURCE	IDENTIFIER
Freund adiuvant incomplete	Sigma Aldrich	f5506
Glutamax	GIBCO	35050-038
Heparin	Sigma Aldrich	H3393
hLIF	GIBCO	PHC9484
IMDM	GIBCO	12440053
Insulin from bovine pancreas	Sigma Aldrich	I1882
Laemmli	Sigma	S3401-L
Laminin	Roche	11243217001
Leukocyte Activation Cocktail	BD Biosciences	550583
Lipopolysaccharide (LPS)	Enzo life sciences	ALX-581-013-L002
MACS BSA Stock Solution	Milteny	130-091-376
Mersalyl acid	Sigma Aldrich	M9784
Minimum Essential Medium Eagle	Sigma Aldrich	M7278
Monosodium glutamate	Sigma	1446600
Mouse differentiation supplement	Stem cells Technologies	05703
Mycobacterium Tuberculosis H37Ra	Difco	231141
N2 supplement	ThermoFisher	17502-048
Neurobasal medium	GIBCO	21103-049
NeuroCult proliferation supplements	Stem cells Technologies	05701
Normal goat serum	Invitrogen	10000C
Pen/strep	Invitrogen	151401
Percoll	Sigma-Aldrich	GE17-0891-02
Pertussis Toxin from Bordetella pertussis	List Biological Laboratories	181
r(MOG 35-55)	Espikem	EPK 1
Recombinant human EGF	Peprotech	AF-100-15
Recombinant Human FEF-basic	Peprotech	100-18B
Recombinant Human Leukemia Inhibitory Factor	GIBCO	PHC9484
Recombinant murine M-CSF	Miltenyi Biotec	130-101-706
Recombinant SDH subunit A	Cloud-clone corp	RPJ784Mu01
RNAlater	QIAGEN	76104
RPMI	GIBCO	31870
ROCK inhibitor Y27632	Calbiochem	688000
SB 431542	Invivogen	inh-sb43
SC-58125	Sigma Aldrich	PZ0139
Sodium succinate dibasic hexa-hydrate	Sigma Aldrich	S2378
Thapsigargin	Sigma	T9033
Trichloroacetic Acid	Sigma	T0699
Trypsin-EDTA (0.05%), phenol red	GIBCO	25300-054
Ultima Gold liquid scintillation cocktail	PerkinElmer	6013329
Critical Commercial Assays		
Fix/Perm Transcription Factor Buffer Set	BD Biosciences	562574
High Capacity cDNA Reverse Transcription kit	applied biosystems	4368813
LDH-Cytotoxicity Assay kit	abcam	ab102526
LIVE/DEAD Fixable Yellow Dead Cell Stain Kit	ThermoFisher	L34959
LS columns	Milteny	130-041-306
NeuroCult NS-A proliferation kit (Human)	Stemcell	5750
Nitrite/Nitrate Assay Kit	Sigma-Aldrich	23479-1KT-F
Prostaglandin E2 ELISA Kit	Caymanchem	514010

(*Continued on next page*)

Continued		
REAGENT or RESOURCE	SOURCE	IDENTIFIER
RNeasy Micro Kit	QIAGEN	74006
RNeasy Midi kit	QIAGEN	73442
Succinate Dehydrogenase Activity Colorimetric Assay Kit	BioVision	K660-100
TaqMan Fast Universal PCR Master	applied biosystems	4352042
Taqman Gene Expression assay: *18S* (4318839)	Life Technologies	# 4331182
Taqman Gene Expression assay: *ACTB*	Life Technologies	# 4331182
Taqman Gene Expression assay: *Arg1* (Mm00475988_m1)	Life Technologies	# 4331182
Taqman Gene Expression assay: *Bst1* (Mm00477672_m1)	Life Technologies	# 4331182
Taqman Gene Expression assay: *Cd69* (Mm01183378_m1)	Life Technologies	# 4331182
Taqman Gene Expression assay: *SUCNR1* (Hs00908230_m1)	Life Technologies	# 4331182
Taqman Gene Expression assay: *Sucnr1* (Mm00519024_m1)	Life Technologies	# 4331182
Taqman Gene Expression assay: *Il1b* (Mm00434228_m1)	Life Technologies	# 4331182
Taqman Gene Expression assay: *Il12b* (Mm01288989_m1)	Life Technologies	# 4331182
Taqman Gene Expression assay: *Il15* (Mm00434210_m1)	Life Technologies	# 4331182
Taqman Gene Expression assay: *Il15ra* (Mm04336046_m1)	Life Technologies	# 4331182
Taqman Gene Expression assay: *Mrc1* (Mm01329362_m1)	Life Technologies	# 4331182
Taqman Gene Expression assay: *Nos2* (Mm00440502_m1)	Life Technologies	# 4331182
Taqman Gene Expression assay: *Ptgs2* (Mm00478374_m1)	Life Technologies	# 4331182
Taqman Gene Expression assay: *Tnf* (Mm00443258_m1)	Life Technologies	# 4331182
Taqman Gene Expression assay: *Ust* (Mm00616790_m1)	Life Technologies	# 4331182
Deposited Data		
The microarray data have been deposited in ArrayExpress	Affymetrix	Accession numbers E-MTAB-5579 and E-MTAB-5586
Experimental Models: Cell Lines		
Human cell line: human BJ fibroblasts	ATCC (CRL-2522)	N/A
Human cell line: human fetal NSCs	Vescovi's lab	N/A
Human cell line: human iNSCs	Edenhofer's lab	N/A
Mouse cell line: L-929	ATCC (NCTC clone 929)	N/A
Mouse cell line: BMDM	Pluchino's lab	N/A
Mouse cell line: BV2	Spillantini's lab	N/A
Mouse cell line: MFs (mouse fibroblasts)	Pluchino's lab	N/A
Mouse cell line: iNSCs	Edenhofer's lab	N/A
Mouse cell line: NSCs	Pluchino's lab	N/A
Mouse cell line: Sucnr1$^{-/-}$ NSCs	Pluchino's lab	N/A
Experimental Models: Organisms/Strains		
C57BL/6 mice	Charles River	c57bl6
Sucnr1$^{-/-}$ mice	NOVARTIS AUSTRIA	N/A
Recombinant DNA		
pRRLsinPPT-hCMV	N/A	N/A
pCT-f-GFP	System Bioscieces	CYTO120-PA-1
Software and Algorithms		
R/Bioconductor	N/A	N/A
Bioconductor pdInfoBuilder package	https://www.bioconductor.org/packages/release/bioc/html/pdInfoBuilder.html	N/A
Bioconductor oligo package	(Carvalho and Irizarry, 2010)	N/A
Bioconductor limma package	(Ritchie et al., 2015)	N/A

(*Continued on next page*)

Continued		
REAGENT or RESOURCE	SOURCE	IDENTIFIER
Bioconductor TopGO package	https://bioconductor.org/packages/release/bioc/html/topGO.html	N/A
Bioconductor Gage package		N/A
GraphPad Prism version 6.00 for Mac, GraphPad Software, La Jolla California USA	https://www.graphpad.com	N/A
Other		
Microvettes	Sarstedt	CB300Z

CONTACT FOR REAGENT AND RESOURCE SHARING

Further queries and reagent requests may be directed and will be fulfilled by the lead contact, Stefano Pluchino (spp24@cam.ac.uk).

EXPERIMENTAL MODEL AND SUBJECT DETAILS

Six independent *in vivo* transplantation studies were performed on a total n = 175 C57BL/6 female mice (weight 20 gr) affected by myelin oligodendrocyte glycoprotein (MOG)-induced experimental autoimmune encephalomyelitis (EAE) (Pluchino et al., 2003). Mice were housed in a controlled environment with a 12:12 hr light-dark cycle with food and water provided *ad libitum*. This research has been regulated under the Animals (Scientific Procedures) Act 1986 Amendment Regulations 2012 following ethical review by the University of Cambridge Animal Welfare and Ethical Review Body (AWERB). Animal work was covered by the PPL 80/2457 (to S.P.).

All human cell lines were cultured at 37°C in a controlled humidified atmosphere of 5% CO_2. Human BJ fibroblasts (hBJFs) were purchased from ATCC (CRL-2522) and cultured as adherent cells in hBJF medium [DMEM high glucose (GIBCO), 10% FBS, 1% GlutaMAX (GIBCO), 1% pen/strep (Invitrogen)] until they reached confluency (80%–90%). The day of passage, hBJFs were washed with PBS. Trypsin (0.05% in DMEM) was added at 37°C and inactivated after for 3 min with hBJF medium (2:1). Cells were collected and then split 1:3 for expansion.

Human induced Neural Stem Cells (hiNSCs) were obtained from direct reprogramming of hBJFs in Edenhofer's lab, as previously described (Meyer et al., 2015). Briefly, hBJFs were infected with Oct4-, Klf4-, Sox2- and c-*myc*-Sendai viruses and cultured in neuro-induction medium (NIM) [1:1 DMEM/F12 (Life Technologies): NeuroCult^{TM} basal medium (GIBCO), 1X B27 (GIBCO), 1X N2 (ThermoFisher), 1% pen/strep (Invitrogen), 1% GlutaMAX(GIBCO), 10 ng/ml hLIF (GIBCO), 3 μM ChiR (Axon MedChem), 2 μM SB (Invitrogen)]. 17 days after infection cultures showed morphological changes and NSC colonies formation. hiNSCs were cultured as adherent monolayers in NIM and culture media was completely replaced every other day. When cells reached confluency of 50%–60%, they were enzymatic dissociated with Accumax^{TM} (Ebioscience) at 37°C for 5 min. Cells were then centrifuged at 300 g for 5 min and re-plated 1:6 on 6 well plates pre-coated with Matrigel^{TM} (BD Bioscience). 10 mM ROCK inhibitor Y27632 (Calbiochem) was added upon seeding. Mycoplasma negative human cells at passage $n \leq 20$ were used in all experiments. RNA and protein extracts from human fetal NSCs (hNSCs) (Mazzini et al., 2015) were provided by Angelo Vescovi (Milano, Italy).

All mouse cell lines were cultured at 37°C in a controlled humidified atmosphere of 5% CO_2. Mouse induced Neural Stem Cells (iNSCs) were obtained from direct reprogramming of Oct4-GiP Mouse Embryonic Fibroblasts (MEFs) from C57BL/6 mice in Edenhofer's lab. Briefly, Oct4-GiP MEFs were infected with retroviruses encoding for Sox2, Klf4, and c-Myc, as previously described (Thier et al., 2012). iNSCs colonies were picked 19 days post infection and cultured as either small cellular aggregates (i.e., neurospheres) or adherent monolayers in iNSCs medium [DMEM/F12 (Life Technologies), 1% pen/strep (Invitrogen), 1X N2 (ThermoFisher), 10 ng/ml purified human recombinant (EGF, Peprotech), 10 ng/ml human recombinant basic fibroblast growth factor (bFGF, Peprotech)]. For maintenance of adherent cells, laminin (1:100, Roche) was added to the iNSCs medium upon seeding. When neurospheres reached a diameter of 150-200 μm (or confluency of 70%–80% for adherent cells), cells were collected and harvested in a 15 mL tube (Falcon) and centrifuged at 300 g for 8 min. The supernatant was removed, and the pellet was dissociated by enzymatic digestion with Accumax^{TM} (Ebioscience) at 37°C for 10 min. In case of adherent cells, culture medium was instead removed, cells were washed in PBS, detachment was performed with Accumax^{TM} (Ebioscience) at 37°C for 10 min. Then fresh iNSCs medium was added, and cells were centrifuged at 300 g for 8 min. The number of viable cells was determined by trypan blue exclusion and viable cells were re-seeded at clonal density 9,700 cells/cm^2. New iNSCs medium was added to each flask every other day. Mycoplasma negative iNSCs at passage $n \leq 25$ were used in all experiments.

Somatic NSCs and *Sucnr1*$^{-/-}$ NSCs were obtained in Pluchino's lab from the subventricular zone (SVZ) of 7-12 week old (18-20 g) female C57BL/6 mice (Charles River, UK) and *Sucnr1*$^{-/-}$ C57BL/6 mice (Rubic et al., 2008) respectively, as previously described (Vescovi and Snyder, 1999). C57BL/6 *Sucnr1*$^{-/-}$ mice were provided by José M. Carballido (Novartis) (Rubic et al., 2008). Briefly, mice were humanely culled by cervical dislocation followed by decapitation, the parietal bones were cut cranially to caudally using micro-surgery scissors, and the brains removed. A brain slice matrix was used to obtain 3 mm thick brain coronal sections starting

from 2 mm after the anterior pole of the brain. The SVZ of the lateral ventricles was isolated from coronal sections using iridectomy scissors. Tissues derived from at least 2 mice were pooled to generate cultures. Dissected tissues were transferred to a 15 mL tube with digestion medium [early balance salt solution (EBSS, GIBCO), papain (1 mg/ml, Worthington), ethylenediaminetetraacetic acid (EDTA) (0.2 mg/ml, Sigma-Aldrich) and L-cysteine (0.2 mg/ml, Sigma-Aldrich)] and incubated for 45 min at 37°C on a rocking platform. At the end of the incubation, the tube was centrifuged at 200 g for 12 min, the supernatant was removed and the pellet was mechanically disaggregated with 2 mL of EBSS. The pellet was centrifuged again at 200 g for 12 min and then dissociated with a 200 μL pipette and seeded in complete growth medium (CGM). CGM was constituted of mouse NeuroCult^{TM} basal medium (Stem Cell Technologies) plus mouse NeuroCult^{TM} proliferation supplements (Stem Cell Technologies) added with 2 μg/ml heparin (Sigma-Aldrich), 20 ng/ml EGF and 10 ng/ml bFGF. After approximately 4-7 days, a small percentage of the isolated cells begun to proliferate, giving rise to neurospheres. When neurospheres reached the necessary dimension (150-200 μm diameter), the cells were harvested in a 15 mL tube and centrifuged at 100 g for 8 min. The supernatant was then removed and the pellet dissociated by enzymatic digestion with Accumax^{TM} at 37°C for 10 min. The number of viable cells was determined by trypan blue exclusion and viable cells were re-seeded at clonal density 8,000 cells/cm^2. Mycoplasma negative NSCs at passage $n \leq 25$ were used in all experiments.

Mouse Fibroblasts (MFs) were prepared in Pluchino's lab from the kidneys of adult C57BL/6 female mice as previously described and immortalized (Grupp and Müller, 1999). MFs were cultured as adherent cells in fibroblasts medium [DMEM high glucose (GIBCO), 10% FBS, 1% GlutaMAX (GIBCO), 1% pen/strep (Invitrogen)] until they reached confluency (80%–90%). The day of passage, cells were washed with PBS. Trypsin (0.05% in DMEM) was added at 37°C and inactivated for 3 min with fibroblasts medium (2:1). Cells were collected and then split 1:3 for expansion.

Bone marrow derived macrophages (Mφ) were obtained in Pluchino's lab from the bone marrow of C57BL/6 female mice, as previously described (Masters et al., 2010). Briefly, 9-10 weeks-old C57BL/6 female mice were anesthetized with 2% isoflurane and killed by cervical dislocation. Bone marrow was flushed from femurs and tibiae and bone marrow progenitor cells were cultured for 6 days on Petri dishes (Thermo Scientific) in Mφ medium [DMEM high glucose (GIBCO), 10% FBS, 1% pen/strep (Invitrogen) and 10% of macrophage colony-stimulating factor (M-CSF) conditioned media from L-929 fibroblast cells].

L-929 fibroblast (NCTC clone 929) cells were purchased from ATCC and grown as adherent cells in L-929-medium [RPMI media, 10% Foetal Calf Serum (GIBCO) and 1% pen/strep (invitrogen)] until they reached confluency (80%). The day of passage cells were washed with PBS. Trypsin (0.05% in DMEM) was added at 37°C and inactivated after for 10 min with, L-929-medium (2:1). Cells were collected and spun at 300 g for 5 min, and then re-seeded 1:10. Conditioned medium was collected from cultures and filtered through a membrane filter (0.22 μm pore diameter) to remove cells and debris and frozen (−80°C) until use. M-CSF conditioned media from L-929 fibroblast cells were used for Mφ cultures unless otherwise stated.

The BV2 microglial cell line was provided by Maria Grazia Spillantini (Cambridge, UK). For normal expansion, cells were cultured in BV2 expansion medium [DMEM high glucose (GIBCO), 2% FBS, 1% pen/strep (Invitrogen)] until they reached confluency (70%). The day of passage cells were washed with PBS. Trypsin (0.05% in DMEM) was added at 37°C and inactivated after for 3 min with BV2 expansion medium (2:1). Cells were collected and spun at 300 g for 5 min, and then re-seeded at 4,200 cells/cm^2 for expansion.

METHOD DETAILS

Mouse iNSC/NSC proliferation, viability and differentiation *in vitro*

Cellular viability of iNSCs/NSCs was assessed by vital stain exclusion (trypan blue staining) and a continuous growth curve was built up by seeding cells at clonal density. The linear growth curve was generated extimating the total number of cells by multiplying the growth rate (i.e., number of live cells divided by the number of seeded cells) for the total number of cells present at the previous time point. The mean number of cells per time point (±SEM) was reported to build the linear trend line. The daily growth rate was obtained dividing the growth rate by the number of days per passage. Viability was defined as the percentage of viable cells over dead cells (±SEM). For differentiation, cells were seeded on 13 mm glass coverslips pre-coated with Matrigel^{TM} (8x10^4 cells/coverslips, BD Bioscience) and cultured in 400 μL differentiation medium [NeuroCult^{TM} basal medium (Stem cells Technologies), 10% mouse differentiation supplement (Stem cells Technologies), 1% pen/strep (Invitrogen)], as previously described (Pluchino et al., 2008). Half of the medium was replaced with fresh differentiation medium after 3 days. After 3 more days (6 days in total), coverslips were washed with PBS and fixed with 4% paraformaldehyde (PFA, Sigma-Aldrich) and 2% sucrose in PBS.

For immunofluorescence staining, cells were rinsed with PBS, and then blocked for 1 hr at room temperature (RT) in blocking buffer (0.1% Triton X-100 and 10% secondary antibody species serum in PBS). The following primary antibodies diluted in blocking buffer were used: anti-nestin (1:200, Abcam), anti-SOX2 (1:100, Abcam), anti-glial fibrillary acidic protein (GFAP) (1:500, Abcam), anti-β-tubulin-III (1:500, Covance), anti-O4 (1:400, R&D). Primary antibodies were incubated at 4°C overnight. Cells were then washed in PBS with 0.1% Triton X-100 and incubated with the appropriate fluorescent secondary antibodies (1:1,000 Alexa Fluor 405, 488, 555, 647, Invitrogen) 1 hr at RT. After washing in PBS, nuclei were counterstained with 4',6-diamidino-2-phenylindole (DAPI) (1:10,000, Invitrogen) for 3 min and then mounted with Dako mounting kit (Fluka). Nonspecific staining was observed in control incubations in which the primary antibodies were omitted. For quantification, images were acquired on a CCD camera (DC 480, Leica) under a fluorescence microscope (Olympus, BX51) with a 40X objective on 6 regions of interest (ROI) of each coverslip. Images were analyzed and prepared using ImageJ software. Data were represented as the percentage of positive cells over the total of DAPI positive cells ± SEM, from a total of $n \geq 3$ independent experiments.

Fluorescence-activated cell sorting (FACS) analysis

For the analysis of the expression of surface molecules on iNSCs/NSCs, cells were harvested and dissociated for counting, as previously described. A total of $5x10^5$ cells were kept in the blocking solution [2% FBS (GIBCO) in PBS] for 15 min. Cells were then incubated for 30 min, at RT with fluorescence-conjugated antibodies: anti-CD44-fluorescein isothiocyanate (FITC) (1:100, BD Biosciences), anti-alpha-4-integrin-phycoerythrin (PE) (1:100, Abcam), anti-L-selectin-allophycocyanin (APC) (1:100, BD Biosciences), anti-CX3C chemokine receptor 1 (CX3CR1)-PE (10 μl, R&D), anti-CXC chemokine receptor type 4 (CXCR4)-PE (1:3, BD Biosciences), anti-C-C chemokine receptor type 2 (CCR2)-APC (10 μl, R&D). After incubation cells were rinsed with PBS and fixed in 0.5% PFA in PBS. FACS analyses were carried out on a Cyan-ADP (Dako Cytomation) and data were analyzed using FlowJo (Treestar).

Ex vivo FACS characterization of inflammatory infiltrates was performed as it follows. Mice were deeply anesthetized, perfused with saline-EDTA. Tissues were isolated, kept in ice-cold complete IMDM medium [5% FBS, 1% GlutaMAX (GIBCO), 1% pen/strep (Invitrogen) in IMDM (GIBCO)], sectioned into small pieces ($\approx$5-10 mm^3) and incubated at 37°C with 1 mL of digestion buffer [2 mg/ml collagenase (Sigma), 0.2 mg/ml dispase (MP Bio), 0.1 mg/ml DNase (New England) in complete IMDM medium] on a shaking platform for 30 min at 700 rpm. After digestion, the cloudy suspension was filtered through 70 μm cell strainer and 9 mL of complete IMDM medium was added.

To remove myelin and debris, 3.3 mL of isotonic 90% percoll solution was added to the samples. Samples were gently mixed and centrifuged at 800 g for 20 min at 4°C with brake speed 0. Myelin debris were carefully removed and pellets were washed 2 times in cold buffer [5% autoMACS Rinsing Solution (Milteny) in 1x MACS BSA solution (Milteny)] and centrifuged at 300 g for 5 min at 4°C. Pellets were then suspended in 100 μL of cold buffer, counted and $CD45^+$ cells sorted using CD45- separation beads (Milteny) and LS columns (Milteny). Cell number was adjusted by pulling together n = 2 EAE mice from the same treatment group and sorting were performed according to manufacturer's recommendations. After sorting, cells were divided in two different staining protocols.

$1x10^6$ $CD45^+$ cells/sample were incubated with rat anti-mouse FcyIII/II receptor (CD16/CD32) blocking antibodies (1:50, BD) for 10 min at 4°C. Samples were then incubated for 30 min at 4°C with the following antibodies: FITC-CD11b (1:200, BD), BUV395-CD3e (1:100, BD), PE-Cy7-CD19 (1:100, BD), APC/Cy7-F4/80 (1:20, Biolegend), AlexaFluor594-GR-1 (1:50, Biolegend), PE-CX3CR1 (1:500, Biolegend), AlexaFluor647-CCR2 (1:50, Biolegend), BV605-CD80 (1:100, BD) and Brilliant Violet 711-CD206/MRC1 (1:40, Biolegend). 7-amino-actinomycin D (7AAD) (1:50, Biolegend) was used to stain dead cells.

$1x10^6$ $CD45^+$ cells/sample were instead seeded with lympho-medium [10% FBS, 1% GlutaMAX (GIBCO), 1% pen/strep (Invitrogen), 10 mM HEPES (Sigma), 55 μM β-mercaptoethanol (GIBCO), 1 mm sodium pyruvate (GIBCO) in RPMI medium (GIBCO)] in a 12 well plate ($1x10^6$ $CD45^+$ cells/well). Cultured cells were supplemented with 1x Leukocyte Activation Cocktail (GolgiPlug, BD) and incubated for 4 hr at 37°C and 5% CO_2. Cells were then harvest and incubated for 30 min at 4°C with the following antibodies: BUV395-CD3e (1:100, BD), AlexaFluor 647-CD4 (1:100, BD), BV786-IL-4 (1:100, BD), BV421-GM-CSF (1:100, BD), PerCP-Cy5.5-IL-17A (1:100, BD), AlexaFluor 488-Foxp3 (1:100, BD), PE-RORγt (1:100, BD) and AlexaFluor 700-IFN-γ (1:100, BD). LIVE/DEAD Fixable Cell stain Kit (ThermoFisher) was used to stain dead cells.

Ex vivo FACS samples were acquired using a BD LSRFortessa cell analyzer flow cytometer and data were analyzed using FlowJo (Treestar). Fluorochrome compensation was performed manually based on single color-marked samples and/or compensation beads (BD Biosciences) when appropriate. All gates were set based on specific fluorescence minus one (FMO) control samples. The following hierarchical gating strategy was employed: 1) exclusion of doublets on an area (FSC-A) versus peak (FSC-H) plot; 2) exclusion of debris on a physical parameter plot (FSC-A versus SSC-A); 3) dead cells were excluded by 7AAD or LIVEDEAD staining; and 4) phenotypic identification of subpopulations (combination of up to 8 markers).

Type-1 inflammatory Mφ or BV2 cells co-cultures with iNSCs/NSCs

After 6 days from bone marrow isolation, Mφ were re-seeded with fresh Mφ medium on 6 or 12 well plates ($5x10^5$ or $1x10^5$ cells/well respectively) for co-culture experiments. For gene microarrays and metabolomic studies, recombinant mouse macrophage colony-stimulating factor (M-CSF) (50 ng/ml, Miltenyi Biotec) and dialyzed (d)FBS were used instead of M-CSF conditioned media from L-929 fibroblast cells and FBS. After 18 hr from re-seeding, Mφ were stimulated by adding 50 ng/ml LPS (Enzo life sciences). To assess the metabolic profile of LPS-activated type-1 inflammatory Mφ ($Mφ^{LPS}$), intracellular and extracellular metabolites were collected at given time points (see also section: *Metabolite extraction and LC-MS analysis*).

For co-culture experiments, treatment cells were dissociated, counted and re-suspended directly in Mφ medium. For co-culture experiments in which specific blockers were used, iNSCs/NSCs/*Sucnr*$^{-/-}$NSCs/hiNSCs were kept in an Eppendorf tube at 37°C ± the irreversible blocker of COX2 *SC-58125* (10 μM, Sigma-Aldrich), the specific inhibitor of human SUCNR1 *4c* (1 μM, Advinus Therapeutics), or PBS (control) for 1 hr prior co-cultures. Cells were then spun at 400 g for 5 min, washed with PBS and re-suspended in Mφ medium. Co-cultures of Mφ and treatment cells were all started 1 hr after LPS stimulation using 0.4 μm-pore size trans-well inserts (Millipore) at a 1:1 ratio. 24 hr after the start of the co-cultures, inserts were removed and Mφ/culture media were isolated for subsequent analysis.

After BV2 cell line reached ~70% confluence, cells were collected and re-seeded at a density of $1x10^5$ cells/3.7 cm^2 on 12 well plates in BV2 experiment medium [DMEM high glucose (GIBCO), 1% pen/strep (Invitrogen)] to avoid excessive activation. After 12 hr from re-seeding 50 ng/ml LPS (Enzo) was added to the medium for stimulation. For co-culture experiments, treatment cells were dissociated, counted and re-suspended in BV2 experiment medium. Co-cultures of BV2 and treatment cells were all started

1 hr after LPS stimulation using 0.4 μm-pore size trans-well inserts (Millipore) at a 1:1 ratio. 6 hr after the start of the co-cultures, inserts were removed and BV2 were isolated for subsequent analysis.

Recombinant SDHA activity and treatment of type-1 inflammatory Mφ

Mouse recombinant succinate dehydrogenase complex subunit A (rSDHA) was purchased from Cloud-Clone Corp. (RPJ784Mu01), reconstituted on ddH_20 and kept at −80°C (stock solution 0.1 μg/μl). Activity measurements were performed using a SDH Activity Colorimetric Assay Kit (#K660-100, BioVision) following manufacturer's instructions. For mouse rSDHA treatment, $M\varphi^{LPS}$ were activated as described and rSDHA was added to the Mφ medium (0.05 μg/ml) 1 hr after LPS stimulation. After 24 hr Mφ were isolated for subsequent gene expression analysis.

Lentiviral fGFP tagging

Cells used for transplantation studies were transduced *in vitro* using a third-generation lentiviral carrier (pRRLsinPPT-hCMV) coding for the enhanced farnesylated (f)GFP, which targets the fluorescent protein to the inner plasma membrane of transduced cells (Cusimano et al., 2012). The functional stability of these cells (in the absence or in the presence of the lentiviral transcript) has been confirmed with clonal and population studies (Pluchino et al., 2003). Briefly, neurospheres were harvested, dissociated to a single cell suspension and seeded at high density [1.5x10^6 in a T75 cm^2 flask (Sigma-Aldrich)] in 5 mL fresh medium. After 12 hr, 3x10^6 T.U./ml of lentiviral vectors were added and 6 hr later additional 5 mL of fresh medium were added. 72 hr after viral transduction, cells were harvested and re-seeded at normal concentration. Transgene expression was measured by FACS analysis before transplantation and > 98% of cells were found to be labeled.

EAE induction, transplantation and behavioral studies

For EAE induction, mice were anaesthetized with isoflurane (4% induction, 1.5% maintenance), and received n = 3 subcutaneous (s.c.) injections (2 flanks and 1 at the base of the tail) of 50 μL containing 200 μg/mouse MOG35-55 (Multiple Peptide System) (Espikem), incomplete Freund's Adjuvant (IFA) and 8 mg/ml Mycobacterium tuberculosis (Scientific Laboratories Supply). 100 μL of Pertussis Toxin (5 ng/μl) (List Biological Laboratories) was injected intravenously (i.v.) on the day of the immunization and again after 48 hr.

Body weight and EAE clinical score (0 = healthy; 1 = limp tail; 2 = ataxia and/or paresis of hindlimbs; 3 = paralysis of hindlimbs and/or paresis of forelimbs; 4 = tetraparalysis; 5 = moribund or death) were recorded daily (Pluchino et al., 2003).

After 11-19 days post immunisation (dpi), mice developed the first clinical signs of diseases (disease onset). At 3 days after disease onset, mice with similar scores were randomly assigned to the different treatment groups. After randomization, mice received a single intracerebroventricular (icv) injection (AP −0.15, ML +1.0 left, DV −2.4) of $fGFP^+$ NSCs, or $fGFP^+$ iNSCs or $fGFP^+$ *Sucnr1*$^{-/-}$ NSCs (1x10^6 in 5 μL PBS). EAE mice untreated or transplanted icv with 1x10^6 MFs or injected icv with 5 μL PBS were used as controls.

Body weight and EAE clinical score were recorded daily up to 50 dpi or 110 dpi. Data were expressed as the mean of EAE score (±SEM) from a total of n ≥ 6 mice per group per time point.

Gait kinematics was assessed using a DigiGait™ (Mouse Specifics Inc., Boston, MA) ventral plane treadmill videography before EAE onset (baseline) and at 10-30 days post transplantation (dpt), as previously described (Eftaxiopoulou et al., 2014). Briefly, the treadmill speed was set at either 10 or 5 cm/sec (depending on each mouse fitness), and the gait of the mice was recorded. An analyzable run was defined as a 5 s video segment without wall or bumper contacts. The mouse was designated as noncompliant and the test was stopped if the mouse failed to accomplish an analyzable run after 3 trials (a 30 s rest was allowed between trials). Criteria for test failure where (i) at least 2 min of test without capturing an analyzable run, and/or (ii) the mouse could not run without contacting the rear wall of the enclosure.

Ex vivo tissue pathology

All pathological quantifications were performed by investigators blind to the treatment groups. The day of sacrifice, mice were deeply anesthetized with an intraperitoneal (i.p.) injection of ketamine 10 mg/ml (Boehringer Ingelheim) and xylazine 1.17 mg/ml (Bayer) in sterile water and transcardially perfused with 1ml EDTA 5M in 500ml saline 0.9% NaCl for 5 min, followed by a solution of 4% PFA in PBS for another 5 min.

Brains and spinal cords were isolated and post-fixed in 4% PFA in PBS at 4°C overnight. Tissues were then washed in PBS and left for at least 48-72 hr in 30% sucrose in PBS at 4°C for cryo-protection. Brains and spinal cords were then embedded in optimum cutting temperature (OCT) medium, frozen with liquid nitrogen and cryo-sectioned (25 μm coronal section thickness for brains and 10 μm axial section thickness for spinal cords) using a cryostat (CM1850, Leica, Wetzlar, Germany) with a microtome blade (A35, Feather, Osaka, Japan). Sections were then stored at −80°C until use.

For quantification of graft survival and inflammatory infiltrates, sections were pre-treated with peroxidase 3% for 15 min, and then were incubated in the blocking solution [PBS + 10% normal goat serum (NGS, Sigma-Aldrich) ± 0.1% Triton X-100] for 1 hr at RT. Primary antibodies were diluted in a solution of PBS + 1% NGS ± 0.1% Triton X-100, and incubated at 4°C overnight. The following primary antibodies were used: anti-GFP (1:250, Invitrogen), anti-CD45 (1:100, Serotec), anti-F4/80 (1:100, Serotec), anti-CD3 (1:250, Abcam), anti-CD20 (1:100, Santa Cruz). The following day, tissues were washed with PBS and incubated for 1 hr with the appropriate secondary biotinilated antibody (1:500, Sigma-Aldrich) diluted in a solution of PBS + 1% NGS, ± 0.1% Triton X-100. Components "A" and "B" of Vectastain Elite ABC kit were mixed for 45 min and the reaction developed by means of 3,3′-Diaminobenzidine (DAB) as

per manufacturer's instructions. The reaction was blocked dipping the section into distilled water and sections were counterstained with hematoxylin. The tissues were then dehydrated (with increasing alcohol solutions), washed in xylene (Merck, Darnstadt, Germany) and mounted with a synthetic mounting medium (EUKITT, Hatfield, PA, USA). The numbers of transplanted fGFP$^+$ cells and the areas of CD45$^+$/F4/80$^+$/CD3$^+$/CD20$^+$ inflammatory infiltrates were calculated on n = 10 equally spaced sections axial brain sections and n = 15 equally spaced axial spinal cord sections. fGFP$^+$ cells and inflammatory contours were outlined using an Olympus BX53 microscope with motorized stage and Neurolucida software (11.07 64-bit, Microbrightfield) and descriptive 3D brain/spinal cord reconstructions were obtained.

For quantification of demyelination and axonal damage, cryostat 10 μm thick spinal cord sections were stained for Luxol fast blue (LFB)/periodic-acid Schiff and Bielschowsky silver impregnation respectively, as previously described (Pluchino et al., 2003). The LFB/Bielschowsky negative areas of n = 15 equally spaced axial spinal cord sections were outlined using an Olympus BX53 microscope with motorized stage and Neurolucida software (11.07 64-bit, Microbrightfield) and descriptive 3D spinal cord reconstructions were obtained. Data are expressed as the percentage (%) of damaged tissue per section (±SEM).

For the quantification of stem cell differentiation and of the % of MPs expressing pro/anti-inflammatory markers *in vivo*, sections were rinsed with PBS, and then blocked for 1 hr at RT in blocking buffer (0.1% Triton X-100 and 10% secondary antibody species serum in PBS). A Fab fragment affinity purified IgG anti-mouse was applied if anti-mouse antibodies were used (1:10, Jackson ImmunoResearch). The following primary antibodies, diluted in blocking buffer, were used at 4°C overnight: anti-GFP (1:250, Invitrogen), anti-nestin (1:200, Abcam), anti-Ki67 (1:250, Abcam), anti-GFAP (1:500, Abcam), anti-NeuN (1:250, Chemicon), anti-MBP (1:100, AbD SeroTec), anti-OLIG2 (1:500, Millipore), anti-von willebrand factor (vWF) (1:200 Abcam), anti-doublecortin (DCX) (1:250, Abcam), anti-fibronectin (1:400, Sigma-Aldrich), anti-CD31 (1:20, BD PharMingen), anti-F4/80 (1:100 Serotec), anti-iNOS (1:100, BD Bioscience), anti-MRC1 (1:400, Abcam). Sections were then washed in PBS with 0.1% Triton X-100 and incubated with the appropriate fluorescent secondary antibodies (1:1,000 Alexa Fluor 405, 488, 555, 647, Invitrogen) for 1 hr at RT. After washing in PBS, nuclei were counterstained with DAPI (1:10,000, Invitrogen) for 3 min and then mounted with Dako mounting kit (Fluka). Nonspecific staining was observed in control incubations in which the primary antibodies were omitted.

Quantification of graft differentiation was obtained from randomized $n \geq 3$ brain ROIs and $n \geq 5$ brain spinal cord ROIs acquired using a confocal microscope (Leica TCS SP5 Microscope). Data are expressed as % of double positive cells over total fGFP$^+$ cells (±SEM) ($\geq$95 fGFP$^+$ cells for each marker of interest were counted). Quantification of MPs expressing pro/anti-inflammatory markers was obtained from the brain ($n \geq 25$ randomized ROIs) and the spinal cords (n = 6 equally spaced entire sections) of EAE mice using a fluorescence microscope (Leica DFC 3000G). Data are expressed as % of double positive area over total F4/80$^+$ area (±SEM).

Blood brain barrier functional analysis

Blood brain barrier (BBB) functional analysis in EAE mice was performed at 30 dpt, as it follows. Briefly, 2% Evans blue dye (EBD, Sigma Aldrich) in physiological saline solution was administered at a dose of 5 μL per g body weight through the tail vein and allowed to circulate for 1 hr. Mice were then deeply anesthetized, perfused with saline-EDTA and the brains snap frozen on dry ice. Brains were cryo-sectioned (25 μm coronal section thickness) using a cryostat (CM1850, Leica, Wetzlar, Germany) with a microtome blade (A35, Feather, Osaka, Japan). Sections were then stored at −80°C until use. To analyze the peri-vascular BBB permeability, sections were fixed in 4% PFA for 15min at RT, washed twice in distilled deionized water and blocked for 1h at RT in blocking solution (0.3% Triton X-100 and 10% goat serum in PBS). Anti-CD31 antibody (1:20, BD PharMingen) was diluted in a solution of PBS + 1% NGS ± 0.3% Triton X-100, and incubated at 4°C overnight. The following day, sections were washed with PBS and incubated for 1h with the appropriate secondary antibody diluted in a solution of PBS + 1% NGS, ± 0.3% Triton X-100 (1:1000, Alexa Fluor 488, Invitrogen). After washing in PBS, nuclei were counterstained with DAPI (1:10,000, Invitrogen) for 3 min and mounted with Dako mounting kit (Fluka). EBD fluorescence intensity (excitation at 620 nm, emission at 680 nm) was calculated on constant ROI areas within the brain (n = 3 mice per group, $n \geq 13$ ROI areas per mouse) using ImageJ software.

Quantification of BBB permeability on brain samples was performed as previously described (Wang and Lai, 2014). Briefly, 50 mg of brain slices were incubated in 500 μL of 0.9% saline for 60 min and centrifuged at 10,000 g for 10 min. Supernatants were treated with 1:1 volume-ratio of 50% TCA and centrifuged at 10,000 g for 20 min. Samples were diluted 1:3 with 95% ethanol and absorbance were read at 620 nm. Data are expressed as μg of EBD per g of tissue (±SEM).

Plasma and cerebrospinal fluid (CSF) sampling

For plasma sampling (at 10 and 30 dpt), the tail vein was punctured and whole blood was sucked by capillarity in EDTA filled Microvettes (Sarstedt) (10-30 μl/mouse). Samples were kept at 4°C until centrifugation (950 g for 5 min). Plasma was then collected from supernatant and stored at −80°C for subsequent analysis.

For CSF sampling (at 10 and 30 dpt), mice were deeply anesthetized with isoflurane (4% induction, 1.5% maintenance), and CSF (3-5 μl/mouse) was obtained as part of a terminal procedure from the cisterna magna, as previously described (Liu and Duff, 2008). CSF samples were initially put on dry ice and then stored at −80°C for subsequent analysis.

Calcium imaging

Cells were counted after dissociation and seeded in their own specific medium plus laminin (Roche) 1:100 (laminin was not added for MFs) on 35 mm glass bottom culture dishes (MatTek Corporation) ($1.5x10^5$ cells/dish). After 2 days in culture, media was changed with Tyrode's solution (isotonic solution resembling CSF composition and containing 129 mM NaCl, 5 mM KCl, 2 mM $CaCl_2$, 3 mM

$MgCl_2$, 30 mM Glucose, 25 mM HEPES) with 5 μM Fluo-4AM (Life Technologies) for 30 min at 37°C. Cells were then washed twice (15 min) with fresh Tyrode's solution. The dish was then mounted in a home-made microfluidic chamber and put on the stage of a Leica DMI 6000B inverted live imaging microscope in a controlled humidified atmosphere of 5% CO_2 at 37°C. The chamber was connected to a perfusion system to allow a continuous/regular flow of solutions and stimulation with 500 μM succinate dibasic hexa-hydrate (Sigma-Aldrich), 1 μM monosodium glutamate (Sigma-Aldrich) or 1 μM ATP (Sigma-Aldrich) and 10 μM Thapsigargin (Sigma-Aldrich), when necessary.

Cells were recorded for 200 s: 50 s of baseline, 100 s of stimulus and 50-180 s of recovery. Images were acquired with a frequency of 2 frames per sec (fps) using a live cell imaging fluorescence microscope. For quantification, acquired time-lapses were analyzed using ImageJ software. The changes of fluorescence intensity overtime of individual ROIs corresponding to the soma of each cell were quantified and normalized over the background fluorescence. The mean intensity of 10 s (corresponding to 20 fps) of basal recording was considered as F0. Changes in fluorescence intensity are expressed as ΔF/F0, where ΔF = Fi-F0 and Fi is the fluorescence intensity of a ROI at a given time point. Signal quantifications were averaged on the top 25 most responsive cells. Percentage of responsive cells was calculated as % of cells showing a ΔF/F0 ≥ 0.07. Representative pictures of baseline and succinate iNSCs/NSCs/MFs and SUCNR NSCs were pseudolored applying an RGB color model assigning red (RGB: 255, 0, 0) or violet (RGB: 68, 0, 96) according to high or low fluorescence intensities.

ELISA

For measurements of prostaglandin (PG) E2 levels after succinate stimulation, cells were dissociated, counted, and $5x10^5$ cells/500 μl/well per condition were seeded in a 24 well plate. After 6 hr, sodium succinate dibasic hexa-hydrate (500 μM, Sigma-Aldrich) in PBS (or PBS alone) was added to the wells. Cells pre-treated (1 hr before succinate stimulus) with ± *SC-58125* (10 μM, Sigma-Aldrich) or ± *4c* (1 μM, Advinus Therapeutics) were used as controls. After 30 min from succinate stimulation, media were collected, spun at 1,000 g for 5 min, and supernatants stored at −80°C for subsequent analysis.

For measurements of PGE2 levels after co-cultures, media were collected at 24 hr of co-culture spun at 1,000 g for 5 min, and supernatants were stored at −80°C for subsequent analysis.

For *ex vivo* measurements of PGE2 levels in CSF, frozen samples were thawed on ice and n ≥ 2 samples from EAE mice were pulled together. For *ex vivo* measurements of PGE2 levels in plasma, frozen samples were thawed on ice (n = 1 sample from each EAE mouse). Samples were then diluted in ice-cold ELISA buffer (1:10) and analyzed the same day.

All PGE2 levels were determined using PGE2 ELISA kit (Caymanchem) following the manufacturer's instructions. Briefly, 50 μL of samples were added to 96 wells pre-coated plates and incubated for 18 hr at 4°C. Plates were then washed, developed for 60-90 min at RT, and read at 405-420 nm. PGE2 concentration was determined by comparison to the standard curve performing 4-parameter logistic fit.

Uptake experiments with [^{14}C] - labeled succinate

Cells were counted after dissociation and re-plated at high cell-density ($2.4x10^5$ cells/cm^2) the day before the experiment. The following day cells were collected and centrifuged at 300 g for 8 min. Pellet was re-suspended in CGM or iNSCs medium (previously adjusted at pH 6.8) and cells were seeded in a 6 well plate at a final concentration of $5x10^5$ cells/ml/well. Each well was respectively stimulated with [^{14}C] - labeled succinate (American Radiolabelled Chemicals) at final concentration 500 μM (80 nCi [^{14}C] - succinate/mL) in PBS. PBS alone was used as controls. At the corresponding time points, culture media and cells were collected, and centrifuged at 400 g for 5 min. To isolate the extracellular fraction, 500 μL of supernatant from each sample was added to tubes containing 3 mL of Ultima Gold liquid scintillation cocktail (PerkinElmer). To isolate the cellular fraction, the remaining volume in the tube was removed first after a 1,000 g for 5 min spin. Another 1,000 g for 1 min spin was then performed to better separate the pellet from the residual supernatant. Finally, each pellet was dried with blotting paper (to further avoid any extracellular fraction's contamination) and 40 μL of Triton X-100 was added. Cellular fraction was then added to tubes containing 3 mL Ultima Gold liquid scintillation cocktail (PerkinElmer). Total radioactivity was then measured using a TriCarb LSC Counter (PerkinElmer). Radioactive counts were converted into decays per min and subsequently converted into amounts of succinate using a final specific activity of 0.15 mCi/mmol. Data were normalized on total proteins content evaluated by BCA Protein assay kit (Thermo Scientific).

Immunoblotting

Western blots for SUCNR1 were performed on freshly dissociated cells. Cells were collected and spun at 16,000 g for 30 s. Pellets were washed with PBS, and then re-suspended in 50 μL of 1X RIPA buffer (Abcam) with protease (Roche) and phosphatase inhibitors (Thermo Fisher Scientific). Samples were frozen at −80°C until further use.

Western blots after succinate stimulation were performed as it follows. Cells were dissociated, counted, and $1.5x10^6$ cells/1ml/well per condition were seeded in a 6 well plate. After 6 hr, either sodium succinate dibasic hexa-hydrate (Sigma-Aldrich) in PBS (final concentration 500 μM, unless otherwise stated) or PBS alone (control) was added to each well. Cells pre-treated (1 hr before succinate stimulus) with ± *4c* (1 μM, Advinus Therapeutics) were used as controls for hiNSCs studies. After given time points (0min-5min-10min-15min-2hr-6hrs) cells were collected and spun at 16,000 g for 30 s. Pellets were washed with PBS, and then re-suspended in 50 μL (or 25 μL for pP38/P38 WB) of 1X RIPA buffer (Abcam) (with protease/phosphatase inhibitors). Samples were frozen at −80°C and electrophoresis was performed the following day.

Western blots for IL-1β on Mφ were performed after 24 hr of co-culture. Proteins were extracted by pooling 3 wells of a 12 well plate per condition ($3x10^5$ cells in total). First, cells were washed with PBS, and then 1X RIPA buffer (Abcam) with protease (Roche) and phosphatase inhibitors (Thermo Fisher Scientific) was added to each well (30 μl/well). Cells were frozen at −20°C for 1 hr, and then scraped before pooling. Samples were frozen at −80°C and electrophoresis was performed the following day.

Western blots for Hif-1α/PKM2 on Mφ were performed after 24 hr of co-culture. Proteins were extracted by pooling 6 wells of a 12 well plate ($6x10^5$ cells in total) per condition. First, cells were washed with PBS, and then Laemmli buffer (Sigma-Aldrich) 1X final concentration with protease (Roche) and phosphatase inhibitors (Thermo Fisher Scientific) was added to each well (50 μl/well). Cells were scraped before pooling, samples were heated 95°C for 5 mins, and the same volume of extract (25 μl) was loaded for electrophoresis on the same day on a 10% Bis-Tris precasted gel (Life technologies).

For all the other samples (i.e., those stored at −80°C), extracts were instead defrosted, sonicated and proteins were quantified using Pierce BCA Protein Assay kit (Thermo scientific). These samples were then heated at 95°C for 5 mins, and the same amount of protein (50-60 μg of extract for P-p38/p38 or 10-20 μg of extract for all the other targets) per each condition was loaded with 1X NuPAGE LDS sample buffer and NuPAGE sample reducing agent 1X on a 10% SDS-PAGE gel.

After running at 120V, samples were then transferred on Immobilon PVDF filter paper sandwich at run completion (Millipore). The membrane was then blocked with 5% non-fat milk in 0.1% PBS-Tween 20 for 1 hr and then incubated with primary antibodies diluted in 5% non-fat milk in 0.1% PBS-Tween 20 [with phosphatase inhibitors (Thermo Fisher Scientific) in case of P-p38/p38 WB] overnight at 4°C.

The following primary antibodies were used for immunoblotting: anti-HiF1α (1:1,000, Novus Biologicals), anti-PKM2 (1:1,000 Cell Signaling), anti-IL-1β (1:1,000 R&D systems), anti-SUCNR1 (1:500 Novus Biologicals), anti-P-p38 MAPK (1:1,000 Cell Signaling), anti-p38 MAPK (1:1,000 Cell Signaling), anti-SLC13A5 (1:400 ThermoFisher), anti-SLC13A3 (1:1,000 Aviva Systems Biology), anti-β-actin (1:10,000 Sigma-Aldrich), anti-β-tubulin (1:1,000 Sigma-Aldrich). After 3 washes in 0.1% PBS-Tween 20, membranes were incubated for 1 hr at RT with the appropriate HRP-conjugated secondary antibodies: anti-rabbit HRP conjugated secondary (1:10,000 Thermo Scientific), anti-mouse HRP conjugated secondary (1:20,000 Thermo Scientific). Proteins bands were developed using Enhanced Chemiluminescence Substrate (Perkin Elmer) and acquired using a Biorad Chemidoc MP system. The density of each band was quantified using ImageJ software and normalized to housekeeping bands (β-actin or β-tubulin) measured in the same membranes.

Extracellular flux (XF) assays

A $XF24^e$ Extracellular Flux Analyzer (Seahorse Bioscience, Billerica, MA) was used for all XF assays.

For XF assay on Mφ co-cultures, Mφ were seeded 6 days after bone marrow isolation, with fresh Mφ medium on a 24 well XF24 cell culture microplate ($1x10^5$ cells/well) for co-culture experiments. After approximately 18 hr from seeding, Mφ were stimulated by adding 50 ng/ml LPS (Enzo life sciences). Treatment (or control) cells were added at 1:1 ratio, 1 hr after LPS stimulation, using 0.4 μm-pore size trans-well inserts (24 well-size, Millipore). 24 hr after the start of the co-culture the inserts were removed, Mφ medium was replaced with XF medium [Seahorse salt solution (Seahorse Bioscience), 1% glutamine 200 mM, 1% pyruvate 100 mM, 1% FBS, D-glucose (225 mg/50ml final volume)] pH 7.35-7.45, and baseline oxygen consumption rate (OCR) and extracellular acidification rate (ECAR) were measured for 10 reads.

For XF assay on all other cell types, cells were counted after dissociation and seeded in their own specific medium plus laminin (Roche) 1:100 (laminin was not added for MFs) on a 24 well XF24 cell culture microplate ($1x10^5$ cells/well). When cells reached > 90% confluency, media were replaced with XF medium pH 7.35-7.45. Mitochondrial stress protocol was performed using oligomycin, FCCP, rotenone and antimycin (1 μM final concentration).

After the completion of each XF assay, cells were washed with PBS and 25 μL of 1X RIPA buffer (with protease/phosphatase inhibitors) were added to each well. The total protein amount/well was estimated with a BCA Protein assay kit (Thermo Scientific) and used to normalize the OCR and ECAR values of the single well.

Gene expression analysis (microarrays and qRT-PCR)

Ex vivo samples were collected at 10 and 30 dpt. Mice were deeply anesthetized with isoflurane (4% induction) and decapitated. The entire brain and spinal cord were exposed, isolated and stored in RNAlater (QIAGEN) at 4°C until use. Samples were homogenated using a potter and total RNA was extracted using the RNeasy Plus Universal Midi Kit (QIAGEN) following manufacturer's instructions.

Total RNA from Mφ or BV2 microglial cell line in co-cultures was collected at given time points. Before collection, cells were washed with PBS, 350 μL of RLT buffer were added, and samples stored at −80°C until extraction.

Total RNA after succinate stimulation was collected as it follows. Cells were dissociated, counted, and $1.5x10^6$ cells/ml/well per condition were seeded in a 6 well plate. After 6 hr, sodium succinate dibasic hexa-hydrate (500 μM, Sigma-Aldrich) in PBS or PBS alone (control) was added to each well. After 15 min cells were collected and spun at 16,000 g for 30 s. Pellets were washed with PBS, resuspended in 350 μL of RLT buffer and stored at −80°C until extraction.

Total RNA from all *in vitro* samples was extracted using the RNeasy Micro Kit (QIAGEN) following manufacturer's instructions.

For microarrays, samples were prepared according to Affymetrix protocols (Affymetrix, Santa Clara, CA). RNA quality and quantity were ensured using the Bioanalyzer (Agilent, Santa Clara, CA) and NanoDrop (Thermo Scientific, Waltham, MA) respectively. For RNA labeling, 200 ng of total RNA was used in conjunction with the Affymetrix recommended protocol for the Clariom_S chips. The hybridization cocktail containing the fragmented and labeled cDNAs was hybridized to the Affymetrix Mouse Clariom_S GeneChip. The

chips were washed and stained by the Affymetrix Fluidics Station using the standard format and protocols as described by Affymetrix. The probe arrays were stained with streptavidin phycoerythrin solution (Molecular Probes, Carlsbad, CA) and enhanced by using an antibody solution containing 0.5 mg/mL of biotinylated anti-streptavidin (Vector Laboratories, Burlingame, CA). An Affymetrix Gene Chip Scanner 3000 was used to scan the probe arrays. Gene expression intensities were calculated using Affymetrix AGCC software. Downstream analysis was conducted in R/Bioconductor.

The annotation package for the Clariom_S chips was generated with *pdInfoBuilder* (https://www.bioconductor.org/packages/release/bioc/html/pdInfoBuilder.html) using the platform files provided by Affymetrix. The CEL files were then loaded into R, RMA normalized with the *oligo* package, and filtered to only retain probes annotated as "main" (Carvalho and Irizarry, 2010). Differential expression testing was performed using *limma* (Ritchie et al., 2015) and the resulting p.values were corrected with the Benjamini-Hochberg method.

GO enrichment analyses were performed using the topGO package (https://bioconductor.org/packages/release/bioc/html/topGO.html) with the *classic* algorithm and *Fisher* statistic. Microarray heatmaps were generated with the *heatmap.2* function of the *gplots* package with the default clustering methods. The microarray raw data were deposited in ArrayExpress with the accession numbers E-MTAB-5579 and E-MTAB-5586.

For qRT-PCR analysis, equal amounts of RNA were reversed-transcribed using the High Capacity cDNA Reverse Transcription Kit (Applied Biosystems) according to the manufacturer's instructions. cDNA was then quantified with the NanoDrop 2000c instrument (Thermo Scientific) and qRT-PCR was performed with the TaqMan® Universal PCR Master Mix (Applied Biosystems) and TaqMan® Gene Expression Assays for: *Il12b* (Mm01288989_m1, Life Technologies), *Il15* (Mm00434210_m1, Life Technologies), *Il15ra* (Mm04336046_m1, Life Technologies), *Cd69* (Mm01183378_m1, Life Technologies), *Nos2* (Mm00440502_m1, Life Technologies), *Tnf* (Mm00443258_m1, Life Technologies), *Il1b* (Mm00434228_m1, Life Technologies), *Bst1* (Mm00477672_m1, Life Technologies), *Ust* (Mm00616790_m1, Life Technologies) *Arg1* (Mm00475988_m1, Life Technologies), *Mrc1* (Mm00485148_m1, Life Technologies), *Sucnr1* (Mm00519024_m1, Life Technologies), *SUCNR1* (Hs00908230_m1, Life Technologies), *Ptgs2* (Mm00478374_m1, Life Technologies), and *Actb*/18S (Life Technologies) were used as internal calibrators. All samples were tested in triplicate on a 7500 Fast Real-Time PCR System (Applied Biosystems) and analyzed with the 2-ΔΔCT method.

Metabolite extraction and LC-MS analysis

Ex vivo CSF/plasma samples (stored at −80°C) were thawed on ice for metabolites extraction. Samples were diluted in cold methanol (1:10) and put in agitation for 15 min at 4°C (800 rpm). After a centrifugation (20,000 g for 10 min), the supernatants were transferred to a new pre-chilled Eppendorf tube. Samples were concentrated using a SpeedVac (ThermoScientific Savant DNA 120) for 15 min and the pellet was dissolved in cold acetonitrile/water (1:1) using 16X of the initial sample volume. Samples were transferred to pre-chilled autosampler vials (Thermo Fisher) and stored at −80°C for subsequent liquid chromatography coupled to mass spectrometry (LC-MS) analysis.

In vitro samples were collected from Mφ (*INTRA_Metab*) and cell culture media (*EXTRA_Metab*). *INTRA_Metab* were collected from Mφ at 1-2-4-6-8-12-25 hr upon LPS stimulation and at 24 hr after the start of co-cultures (for co-culture experiments). For *INTRA_Metab* extraction, Mφ (originally re-seeded as 1x10^5 cells/well in a 12 well plate) were washed with PBS and kept on ice throughout the procedure. 200 μL of Metabolite Extraction Buffer (MEB) (50% Methanol, 30% Acetonitrile, 20% water, with 100 ng/mL HEPES) was added to each well, and after scraping, extracts from each single well were collected separately. Samples were kept in agitation at 4°C (800 rpm) for 15 min, and then spun at 13,000 g for 15 min at 4°C. 100 μL of the supernatant was transferred to a pre-chilled autosampler vial and stored at −80°C for subsequent LC-MS analysis. *EXTRA_Metab* were collected from Mφ media at 25 hr from LPS stimulation and at 24 hr after the start of co-cultures (for co-culture experiments). For *EXTRA_Metab* extraction, media were collected from Mφ, spun at 1,000 g for 5 min, and 50 μL of the supernatant transferred to a pre-chilled Eppendorf containing 750 μL of MEB. Samples were kept in agitation at 4°C (800 rpm) for 15 min, and then spun at 1,000 g for 10 min at 4°C. 600 μL of the supernatant was transferred to a pre-chilled autosampler vial and stored at −80°C for subsequent LC-MS analysis.

LC-MS analysis was performed on a QExactive Orbitrap mass spectrometer coupled to a Dionex U3000 UHPLC system (Thermo). The liquid chromatography system was fitted with a Sequant ZIC-HILIC column (150 mm x 2.1 mm, 5 μm) and guard column (20 mm × 2.1 mm, 5 μm) from HiChrom, Reading, UK. The mobile phase was composed of 0.1% formic acid (v/v) in water (solvent A), and 0.1% formic acid (v/v) in acetonitrile (solvent B). The flow rate was set at 300 μL/min with the following gradient: 0 min 80% B, 5 min 30% B, 15 min 10% B, 20 min 10% B, 21 min 80% B, 30 min 80% B. The mass spectrometer was operated in full MS and polarity switching mode. Samples were randomized in order to avoid bias due to machine drift. The acquired spectra were analyzed using XCalibur Qual Browser and XCalibur Quan Browser software (Thermo Scientific) by referencing to an internal library of compounds.

QUANTITATION AND STATISTICAL ANALYSES

Statistical analyses were performed with GraphPad Prism (version 5.00 for Mac, GraphPad Software), unless otherwise stated. Statistical analyses of EAE score and mitochondrial stress protocol were performed using a two-way ANOVA analysis, followed by Holm-Sidak post-test. Statistical analysis of DigiGait data was performed with SPSS (version 21 for mac, IBM Software) and outcomes were analyzed using a multivariate general linear model. Metabolic changes were analyzed using a one-way ANOVA, followed by Bonferroni post-test, unless otherwise stated. All remaining differences among groups were tested using a Kruskal-Wallis test,

followed by a Mann-Whitney post-test. Information on number of subjects and experimental replicates are given in the figure legends of the corresponding experiments.

All values are given in the text as mean (±SEM) and a p value < 0.05 was accepted as significant in all analyses, unless otherwise stated.

DATA AND SOFTWARE AVAILABILITY

The accession numbers for the microarray data reported in this paper are ArrayExpress (https://www.ebi.ac.uk/arrayexpress/): E-MTAB-5579 and E-MTAB-5586 (see also Tables S2 and S4).

Cell Stem Cell, Volume *22*

Supplemental Information

Macrophage-Derived Extracellular Succinate Licenses Neural Stem Cells to Suppress Chronic Neuroinflammation

Luca Peruzzotti-Jametti, Joshua D. Bernstock, Nunzio Vicario, Ana S.H. Costa, Chee Keong Kwok, Tommaso Leonardi, Lee M. Booty, Iacopo Bicci, Beatrice Balzarotti, Giulio Volpe, Giulia Mallucci, Giulia Manferrari, Matteo Donegà, Nunzio Iraci, Alice Braga, John M. Hallenbeck, Michael P. Murphy, Frank Edenhofer, Christian Frezza, and Stefano Pluchino

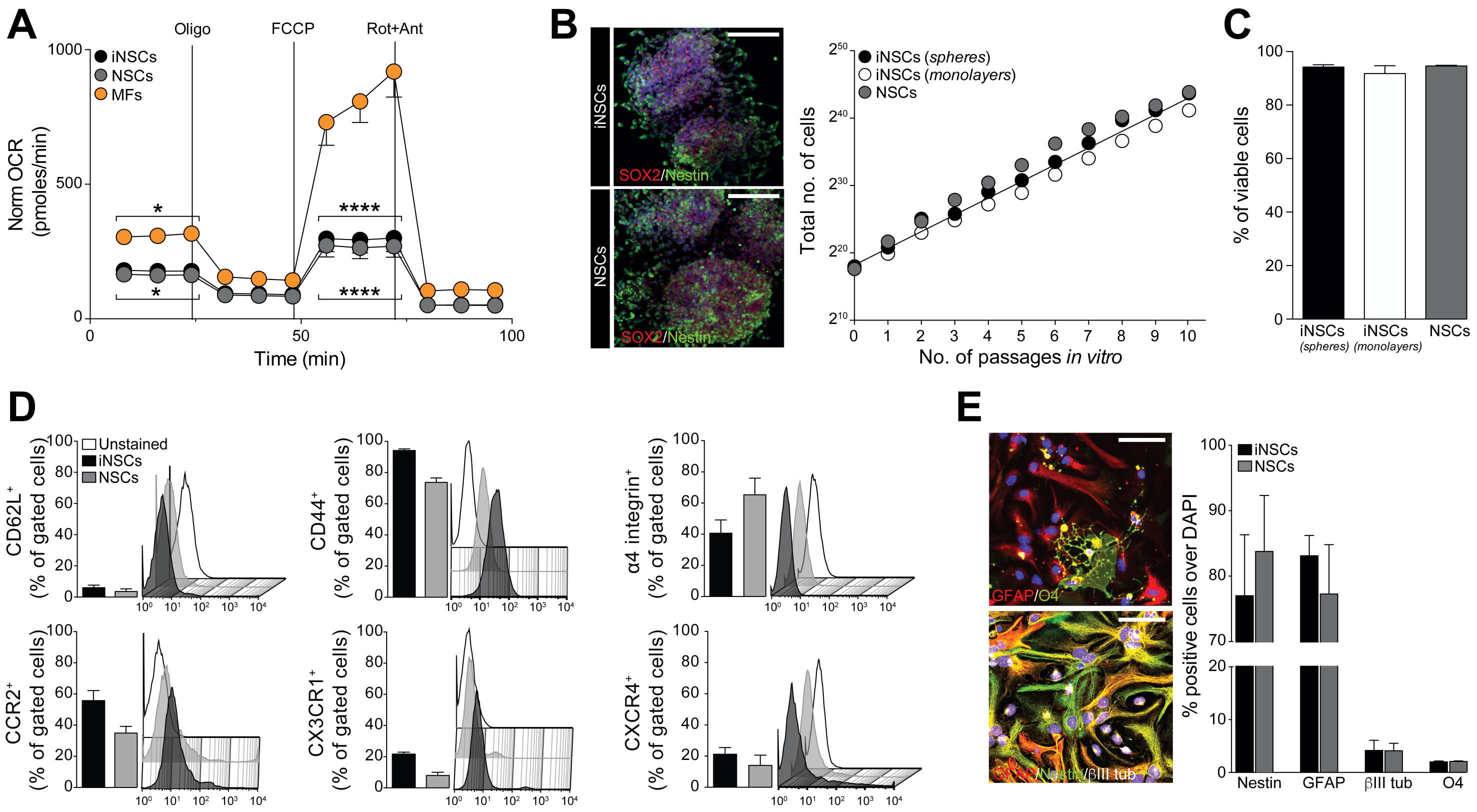

Peruzzotti-Jametti et al. Figure S1

Figure S1. Related to Figure 1. Mouse iNSCs and NSCs *in vitro* features

(A) Extracellular Flux (XF) assay of the Oxygen Consumption Rate (OCR) during a mitochondrial stress protocol. Data are normalized on total protein content and expressed as mean values (± SEM) from n≥ 3 independent experiments.

(B) Representative confocal microscopy images of iNSCs and NSCs growing *in vitro* as $SOX2^+$/$Nestin^+$ neurospheres. Nuclei are stained with DAPI. Linear growth curves of iNSCs and NSCs over 10 passages *in vitro*. Data are mean numbers (± SEM) from n≥ 3 independent experiments per condition.

(C) Cell viability as in B. Data are mean percentages of viable cells (± SEM) from n≥ 3 independent experiments per condition.

(D) Fluorescence-activated cell sorting (FACS) analysis. Data are positive cells over gated cells (mean percentages ± SEM) from n≥ 3 independent experiments per condition.

(E) *In vitro* differentiation properties of iNSCs and NSCs. Representative confocal microscopy images of differentiated iNSCs stained for GFAP, O4, Nestin and βIII tubulin are shown. Nuclei were stained with DAPI. Data are mean percentages of positive cells (± SEM) from n≥ 3 independent experiments per condition.

Scale bars: 100 μm (B), 40 μm (E). *p≤ 0.05 and ****p≤ 0.0001, *vs.* Mouse Fibroblasts (MFs).

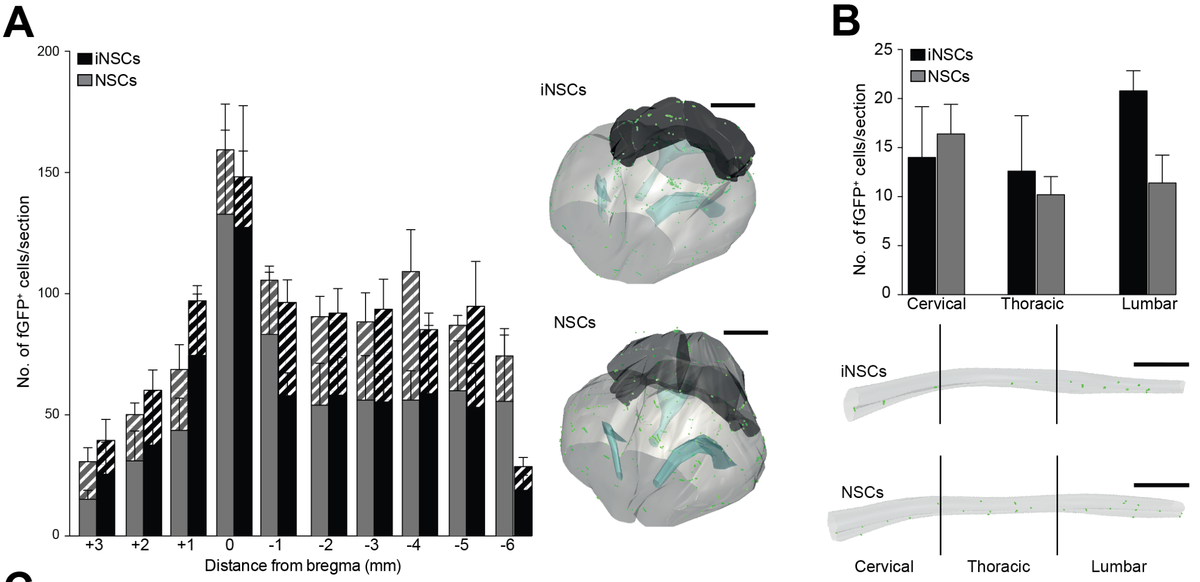

C

iNSCs			NSCs		
**Mean ± SEM**	**Brain**	**Spinal Cord**	**Mean ± SEM**	**Brain**	**Spinal Cord**
**No. of fGFP+ cells**	33,344 ± 7,505	1,896 ± 338	**No. of fGFP+ cells**	29,780 ± 4,551	1,520 ± 237
**% Nestin+/fGFP+**	1.03 ± 1.03	ND	**% Nestin+/fGFP+**	0.90 ± 0.90	1.54 ± 0.81
**% Ki67+/fGFP+**	2.11 ± 0.87	ND	**% Ki67+/fGFP+**	1.73 ± 0.11	ND
**% NeuN+/fGFP+**	2.28 ± 0.65	ND	**% NeuN+/fGFP+**	2.03 ± 0.32	ND
**% GFAP+/fGFP+**	15.04 ± 2.67	23.81 ± 12.40	**% GFAP+/fGFP+**	12.48 ± 2.40	31.34 ± 3.96
**% OLIG2+/fGFP+**	5.12 ± 2.03	ND	**% OLIG2+/fGFP+**	4.89 ± 0.71	ND
**% DCX+/fGFP+**	1.32 ± 0.30	ND	**% DCX+/fGFP+**	1.24 ± 0.20	ND

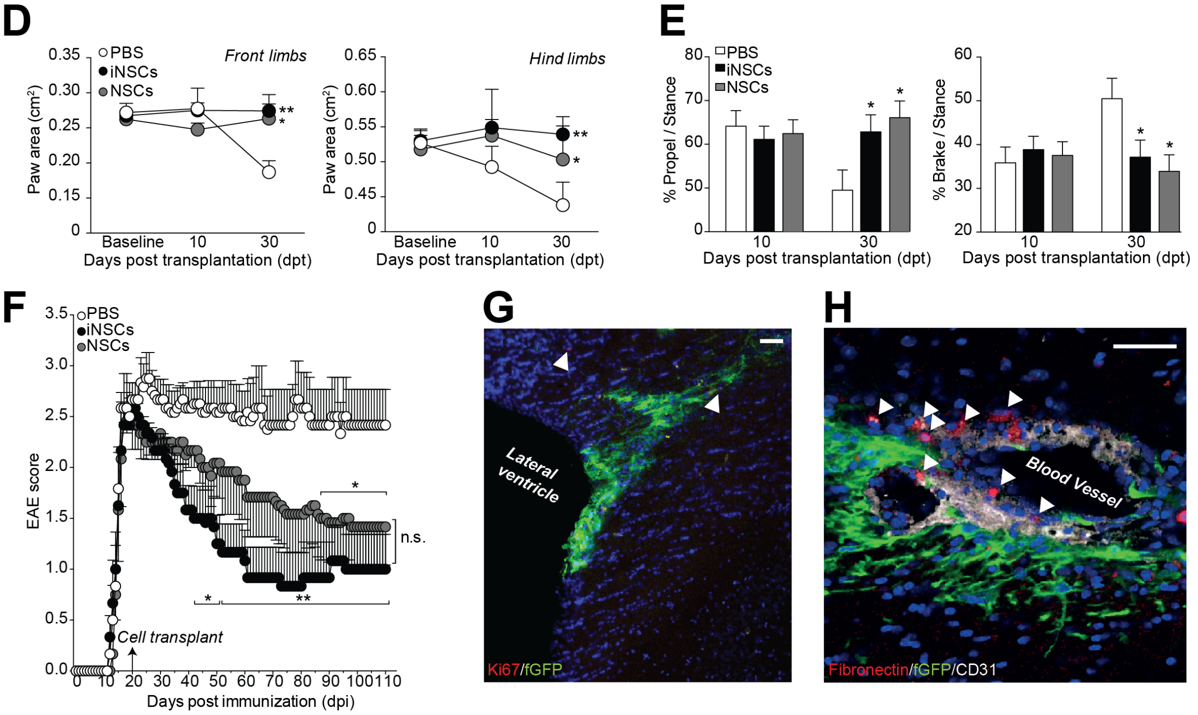

Peruzzotti-Jametti et al. Figure S2

Figure S2. Related to Figure 1. Distribution, efficacy and safety of icv-injected iNSCs and NSCs

(A-B) Distribution in the brain and spinal cord of fGFP$^+$ iNSCs or fGFP$^+$ NSCs injected icv in EAE mice at peak of disease (PD). Stereology-based quantification of whole brain (A) and spinal cord (B) with representative 3D reconstructions (green dots indicate individual fGFP$^+$ cells, contours of the ventricles are in light blue, those of the forebrain or spinal cord are in light grey, and those of the cerebellum are in dark grey). Solid bars in A are data from the left-brain hemisphere, while wide upward diagonal bars are from data of the right brain hemisphere. Data are mean numbers (± SEM) from n≥ 5 mice/group over n= 2 independent experiments. Scale bars: 2 mm.

(C) *In vivo* differentiation profile of transplanted fGFP$^+$ iNSCs or NSCs as in A and B. Data are mean numbers (± SEM) from n≥ 5 mice/group over n= 2 independent experiments.

(D-E) DigiGaitTM ventral plane treadmill videography of EAE mice. (D) Changes in the areas of the paws (front limbs and hind limbs) in iNSC- and NSC-transplanted EAE mice. Data are mean areas (± SEM) from n≥ 5 mice/group. Baseline was assessed before EAE onset. (E) Gait kinematics as in A. Data are percentages of time in which the paw is in the propulsion (propel) or breaking (brake) phase during the stance (± SEM) from n≥ 5 mice/group.

(F) Behavioural outcome of fGFP$^+$ iNSC transplants up to 110 dpi. Data are mean EAE score (± SEM) from n= 6 mice/group.

(G-H) Representative confocal microscopy image of transplanted fGFP$^+$ iNSCs in the brain of a EAE mouse at 110 dpi. Expression of Ki67 by fGFP$^+$ iNSCs is indicated by arrowheads (G). Transplanted fGFP$^+$ iNSCs are negative for fibronectin (arrowheads in H). Scale bars: 50 μm.

*p≤ 0.05 and **p≤ 0.01, *vs.* PBS; n.s.: not significant, ND: not detected.

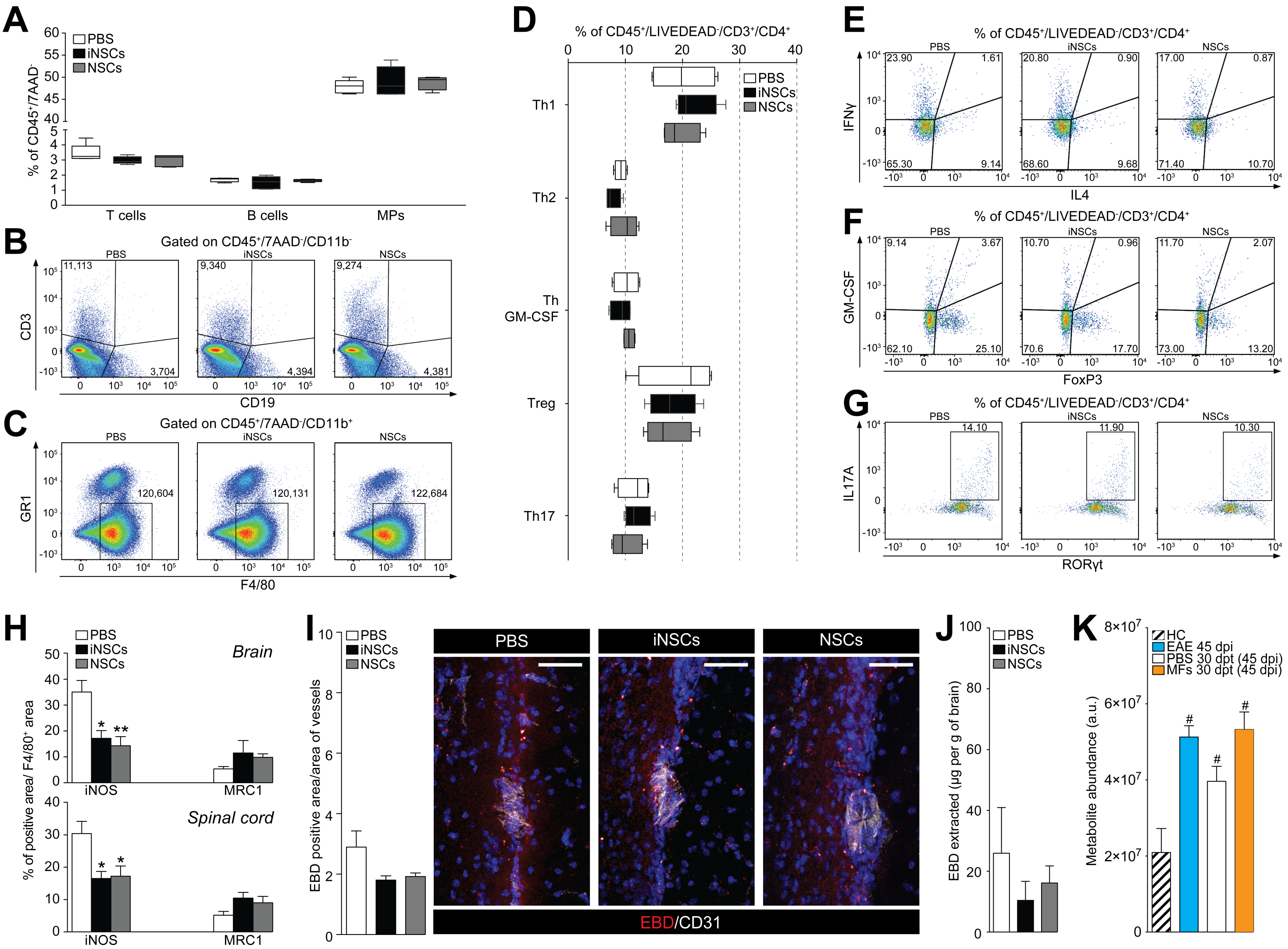

Peruzzotti-Jametti et al. Figure S3

Figure S3. Related to Figure 1. Inflammatory infiltrates and BBB analysis of EAE mice

(A) Flow cytometry *ex vivo* analysis of lymphoid and myeloid cells from the CNS of iNSC- and NSC-transplanted EAE mice at 30 dpt. Cells are gated as $CD45^+/7AAD^-/CD11b^-/CD3^+$ (T cells), $CD45^+/7AAD^-/CD11b^-/CD19^+$ (B cells), $CD45^+/7AAD^-/CD11b^+/GR1^-/F4/80^+$ (MPs). Data are min to max % of marker-positive cells from n≥ 4 pools of mice/group.

(B-C) Representative density plots as in A.

(D) Analysis of the $CD3^+/CD4^+$ T cells subsets as $IFN\gamma^+$ (Th1), $IL4^+$ (Th2), $GM\text{-}CSF^+$ (Th GM-CSF), $FoxP3^+$ (Treg) and $IL17A^+/ROR\gamma t^+$ (Th17). Data are min to max % of marker-positive cells from from n≥ 4 pools of mice/group.

(E-G) Representative density plots as in D.

(H) Quantification of the proportion of $F4/80^+$ cells expressing the type-1 inflammatory MPs marker iNOS, or the anti-inflammatory MPs marker MRC1. Data are mean % of positive-stained area/total section area and % of positive-stained area/$F4/80^+$ area (± SEM), respectively. Data are from n≥ 5 mice/group over n= 2 independent experiments.

(I) EBD-based quantification of peri-vascular BBB leakage/permeability by image analysis. Representative images of $CD31^+$ vessels with EBD staining in the brain of iNSC-/NSC-transplanted and PBS-treated control EAE mice are shown. Nuclei are stained with DAPI. Data are EBD positive area (± SEM) over the corresponding vascular area from n= 3 mice/group.

(J) Quantification of EBD extracted from brain samples as in I. Data are expressed as μg of EBD per g of tissue (± SEM) from n= 3 mice/group.

(K) Quantification of CSF succinate. EAE 45 dpi are EAE mice not subjected to surgery, while MFs are EAE mice injected icv with MFs as control cells. HC, healthy controls. Data are mean arbitrary units (a.u.) (± SEM) from n≥ 3 mice/group.

Scale bars: 50 μm. EBD: Evans blue dye. *p≤ 0.05 and **p≤ 0.01, *vs.* PBS; #p≤ 0.05, *vs.* HC.

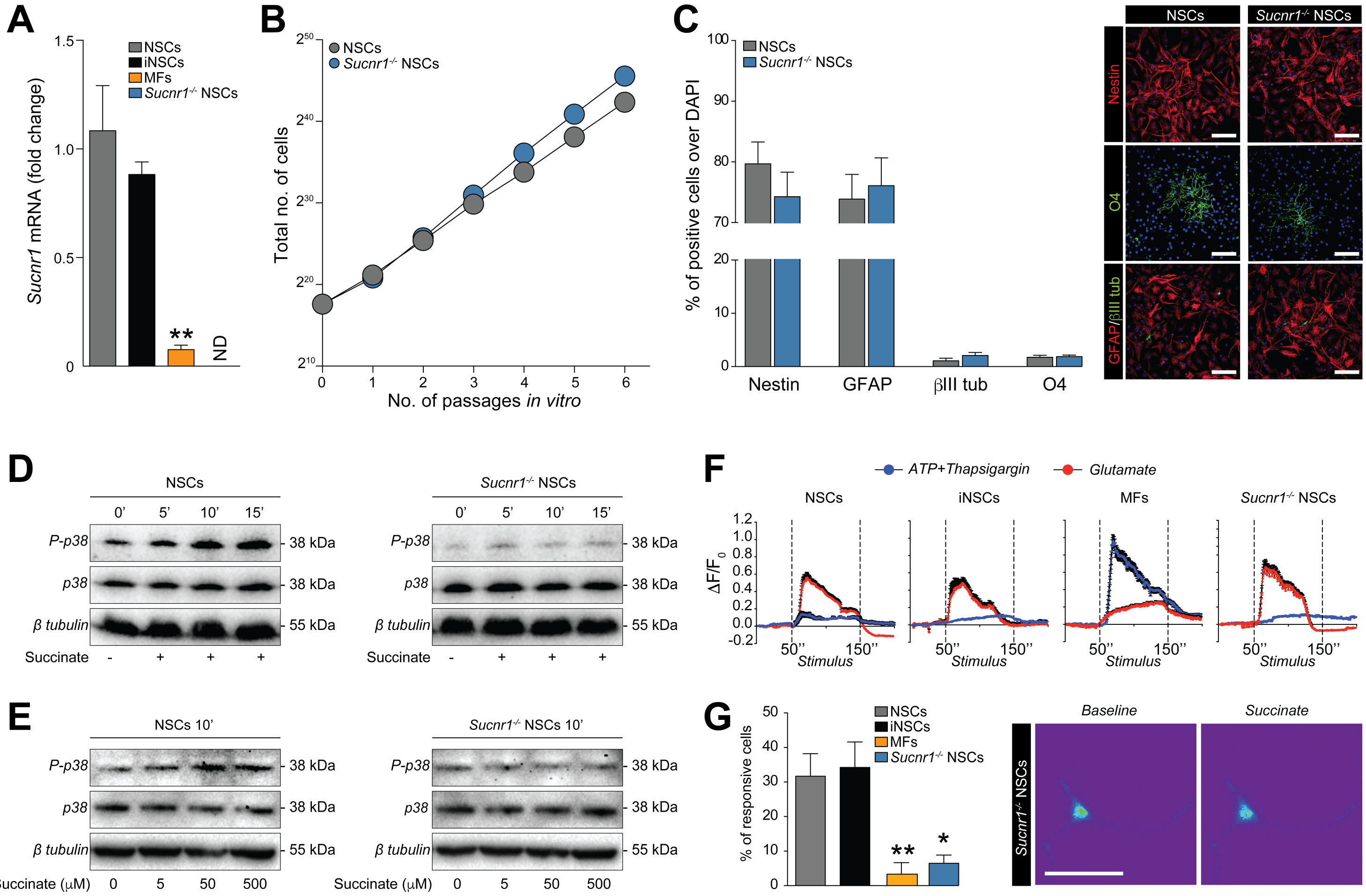

Peruzzotti-Jametti et al. Figure S4

Figure S4. Related to Figure 3. Mouse *Sucnr1-/-* NSCs *in vitro* features

(A) *Sucnr1* mRNA expression relative to *Actb* in mouse cells and *Sucnr1-/-* NSCs. Data are mean fold change (± SEM) *vs.* NSCs from n≥ 3 independent replicates per condition.

(B) Linear growth curves of NSCs and *Sucnr1*$^{-/-}$ NSCs over 6 passages *in vitro*. Data are mean numbers (± SEM) from n≥ 3 independent experiments per condition.

(C) *In vitro* differentiation properties of NSCs and *Sucnr1*$^{-/-}$ NSCs. Representative confocal microscopy images of differentiated cells stained for Nestin, O4, GFAP and βIII tubulin are shown. Nuclei are stained with DAPI. Data are mean percentages of positive cells (± SEM) from n≥ 3 independent experiments per condition.

(D-E) Phospho-p38 MAPK (P-p38) and total p38 MAPK (p38) protein expression in mouse NSCs (as reported in Figure 3G) and *Sucnr1*$^{-/-}$ NSCs after 500 μM succinate at different time points (D), or at 10' under different concentration of succinate (E).

(F) Intracellular Ca^{2+} response after treatment with 500 μM succinate (live staining with Fluo-4AM). Data are % of responsive cells (± SEM) from n≥ 3 experiments. Representative images of *Sucnr1*$^{-/-}$ NSCs (baseline and during stimulation) are pseudocolored with red/blue according to high/low fluorescence intensity.

(G) Intracellular Ca^{2+} response after treatment with 500 μM succinate (live staining with Fluo-4AM). Data are % of responsive cells (± SEM) from n≥ 2 experiments. Representative images of *Sucnr1*$^{-/-}$ NSCs (baseline and during stimulation) are pseudocolored with red/blue according to high/low fluorescence intensity.

Scale bars: 50 μm (C), 25 μm (G). *p≤ 0.05 and **p≤ 0.01, *vs.* NSCs; ND: not detected.

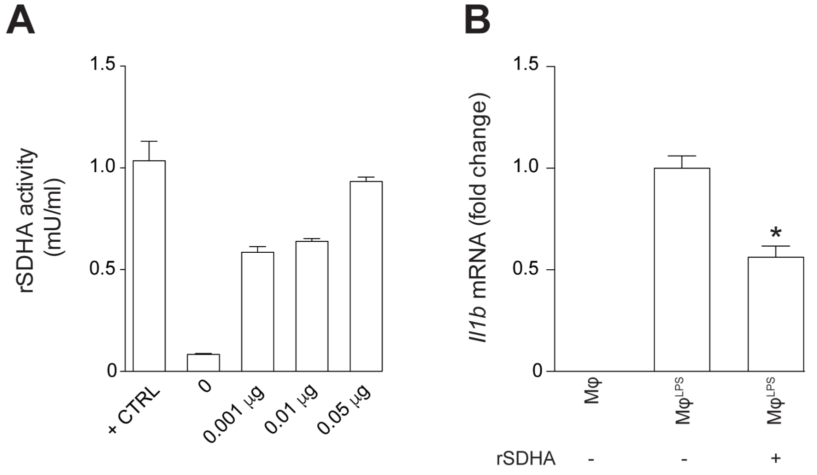

Peruzzotti-Jametti et al. Figure S5

Figure S5. **Related to Figures 4 and 5. Anti-inflammatory effect of recombinant SDHA on type 1 inflammatory Mφ *in vitro***

(A) Activity of increasing concentrations of mouse recombinant complex subunit A (rSDHA) compared to manufacturer's positive control (+ CTRL).

Data are mean numbers (± SEM) from n= 3 independent replicates per condition.

(B) *Il1b* expression relative to *Actb* in Mφ^{LPS} treated with 0.05 μg/ml of mouse rSDHA. Data are mean fold change *vs.* Mφ^{LPS} (± SEM) from n≥ 3 independent experiments per condition.

*p≤ 0.05, *vs.* Mφ^{LPS}.

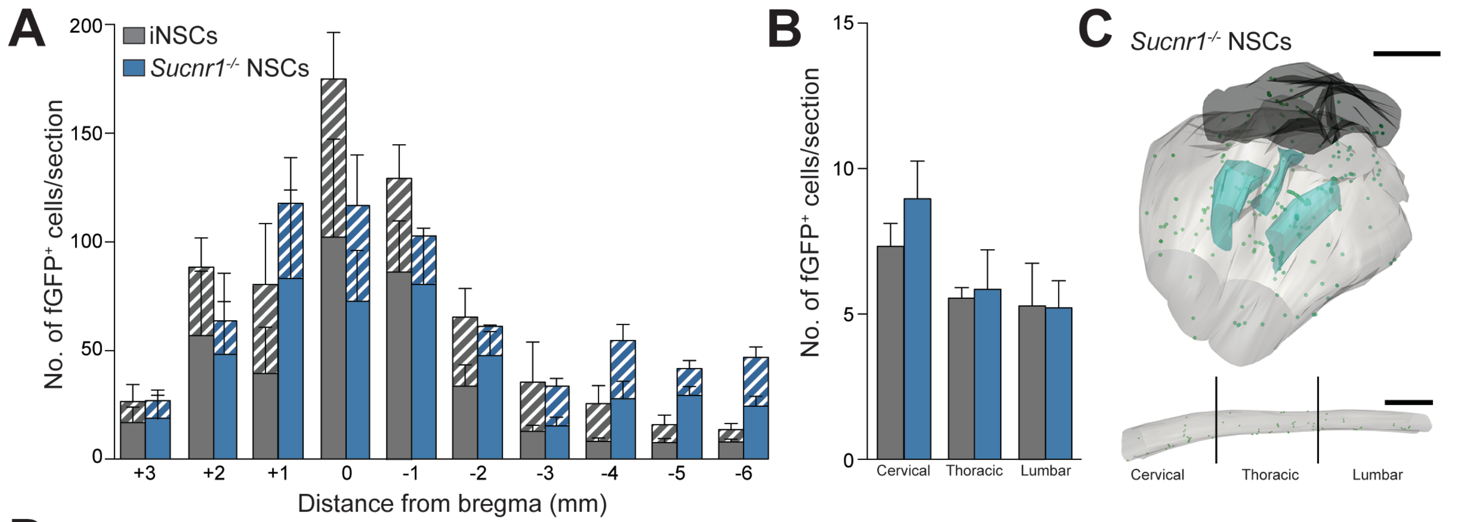

D

NSCs			*Sucnr1*$^{-/-}$ *NSCs*		
**Mean ± SEM**	**Brain**	**Spinal Cord**	**Mean ± SEM**	**Brain**	**Spinal Cord**
**No. of fGFP+ cells**	26,080 ± 7,350	727 ± 71	**No. of fGFP+ cells**	26,740 ± 5,871	801± 92
**% Nestin+/fGFP+**	2.30 ± 0.25	1.42 ± 0.27	**% Nestin+/fGFP+**	3.11 ± 1.19	2.24 ± 0.33
**% Ki67+/fGFP+**	0.62 ± 0.28	0.16 ± 0.16	**% Ki67+/fGFP+**	1.12 ± 0.55	0.46 ± 0.27
**% NeuN+/fGFP+**	0.97 ± 0.40	ND	**% NeuN+/fGFP+**	0.68 ± 0.23	ND
**% GFAP+/fGFP+**	23.64 ± 2.13	31.54 ± 0.49	**% GFAP+/fGFP+**	21.67 ± 2.59	30.17 ± 2.07
**% OLIG2+/fGFP+**	4.54 ± 0.69	1.08 ± 0.45	**% OLIG2+/fGFP+**	6.09 ± 1.69	0.43 ± 0.28
**% DCX+/fGFP+**	0.94 ± 0.20	ND	**% DCX+/fGFP+**	1.94 ± 0.50	ND

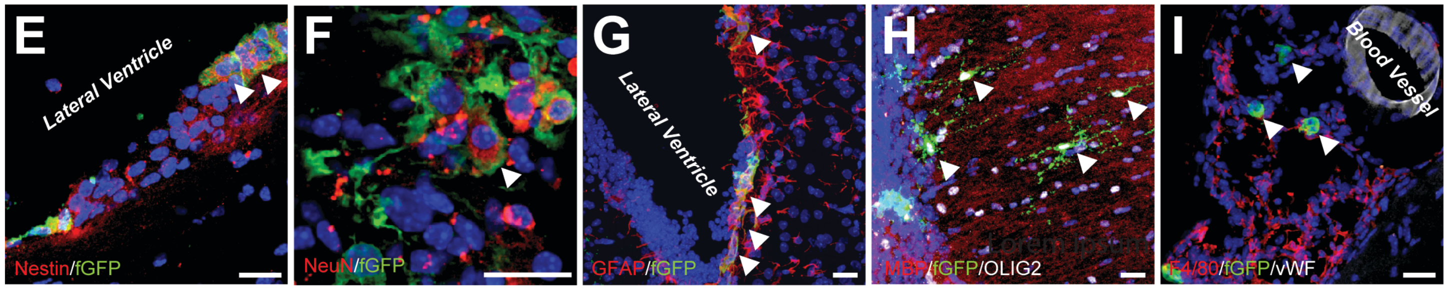

Peruzzotti-Jametti et al. Figure S6

Figure S6. Related to Figure 6. Distribution and differentiation of icv-injected *Sucnr1*$^{-/-}$ NSCs at 30 dpt

(A-B) Stereology-based quantification of whole brain (A) and spinal cord (B) of fGFP^{+} *Sucnr1*$^{-/-}$ NSCs (light blue bars) or fGFP^{+} NSCs (grey bars) injected icv in EAE mice at peak of disease. Solid bars in A are data from the left-brain

hemisphere, while wide upward diagonal bars are from data of the right brain hemisphere. Data are mean numbers (± SEM) from n≥ 4 mice/group.

(C) Representative 3D reconstructions of a brain and spinal cord from a *Sucnr1*$^{-/-}$ NSCs-treated EAE mouse (green dots indicate individual fGFP^{+} cells, contours of the ventricles are in light blue, those of the forebrain or spinal cord are in light grey, and those of the cerebellum are in dark grey).

(D) *In vivo* differentiation profile of transplanted fGFP^{+} *Sucnr1*$^{-/-}$ NSCs or fGFP^{+} NSCs at 30 dpt. Data are mean numbers (± SEM) from n≥ 4 mice/group.

(E-H) Representative images of fGFP^{+} *Sucnr1*$^{-/-}$ NSCs at 30 dpt expressing the neural marker Nestin (E, arrowheads), the mature neuronal marker NeuN (F, arrowhead), the astroglial lineage marker GFAP (G, arrowheads) or the oligodendroglial lineage marker OLIG2 (H, arrowheads).

(I) Confocal microscopy image of a perivascular area with several fGFP^{+} *Sucnr1*$^{-/-}$ NSCs (arrowheads) in juxtaposition with fGFP^{-}/F4/80^{+} MPs.

Nuclei in E-I are stained with DAPI (blue). Scale bars: 2 mm (C), 25 μm (E-I). ND: not detected.
